# Lifestyle-, environmental-, and additional health factors associated with an increased sperm DNA fragmentation: a systematic review and meta-analysis

**DOI:** 10.1186/s12958-023-01054-0

**Published:** 2023-01-18

**Authors:** Anett Szabó, Szilárd Váncsa, Péter Hegyi, Alex Váradi, Attila Forintos, Teodóra Filipov, Júlia Ács, Nándor Ács, Tibor Szarvas, Péter Nyirády, Zsolt Kopa

**Affiliations:** 1 Department of Urology, Semmelweis University, Üllői Ut 78/B, Budapest, H-1082 Hungary; 2 Centre for Translational Medicine, Semmelweis University, Budapest, Hungary; 3 Institute for Translational Medicine, Medical School, University of Pécs, Pécs, Hungary; 4 Institute of Pancreatic Diseases, Semmelweis University, Budapest, Hungary; 5 Department of Interventional Radiology, Semmelweis University, Budapest, Hungary; 6 Department of Obstetrics and Gynecology, Semmelweis University, Budapest, Hungary; 7 Department of Urology, University of Duisburg-Essen and German Cancer Consortium, Essen, Germany

**Keywords:** Male fertility, Infertility, Reproductive medicine, Sperm quality, Prognostic factors, DFI, DNA fragmentation index, SDF, Sperm DNA fragmentation

## Abstract

**Introduction:**

Infertility affects one in every six couples in developed countries, and approximately 50% is of male origin. In 2021, sperm DNA fragmentation (SDF) testing became an evidence-based test for fertility evaluations depicting fertility more clearly than standard semen parameters. Therefore, we aimed to summarize the potential prognostic factors of a higher SDF.

**Methods:**

We conducted a systematic search in three medical databases and included studies investigating any risk factors for SDF values. We calculated mean differences (MD) in SDF with 95% confidence interval (CI) for exposed and non-exposed individuals.

**Results:**

We included 190 studies in our analysis. In the group of associated health conditions, varicocele (MD = 13.62%, CI: 9.39–17.84) and impaired glucose tolerance (MD = 13.75%, CI: 6.99–20.51) had the most significant increase in SDF. Among malignancies, testicular tumors had the highest impact, with a maximum of MD = 11.3% (CI: 7.84–14.76). Among infections, the overall effects of both *Chlamydia* and HPV were negligible. Of lifestyle factors, smoking had the most disruptive effect on SDF – an increase of 9.19% (CI: 4.33–14.06). Different periods of sexual abstinence did not show significant variations in SDF values. Age seemed to have a more drastic effect on SDF from age 50 onwards, with a mean difference of 12.58% (CI: 7.31–17.86). Pollution also had a detrimental effect – 9.68% (CI: 6.85–12.52).

**Conclusion:**

Of the above risk factors, varicocele, impaired glucose tolerance, testicular tumors, smoking, pollution, and paternal age of over 50 were associated with the highest SDF.

**Trial registration:**

CRD42021282533.

**Supplementary Information:**

The online version contains supplementary material available at 10.1186/s12958-023-01054-0.

## Introduction

Fertility rates are declining in western developed countries; currently, approximately 15% of couples are infertile [1]. The World Health Organization (WHO) defines infertility as regular unprotected sexual intercourse without achieving conception within a year [2]. The reason could be multifactorial. However, the male proportion accounts for approximately 50%, and despite a substantial decrease of idiopathic cases in the past years, 30–40% are still of unknown origin [3]. Furthermore, sperm concentration, morphology, and semen volume have all been shown to have deteriorated drastically over the past decades [4].

In recent years, there has been a growing demand for functional, objective parameters reflecting fertility status more clearly than classical parameters. Of these, sperm DNA fragmentation (SDF) and the DNA fragmentation index (DFI) – denoting the percentage of sperm with damaged DNA – seem to be of utmost importance [5]. In 2021, it became the first evidence-based test to be included in the international guideline [6]. However, there is no consensus on how to distinguish between fertile and infertile males based on SDF values [7]. DNA integrity is required for fertilization, and the normal development of the embryo [8]. Accordingly, it has been known that infertile men have more sperm with damaged DNA than fertile men [9]. On the other hand, high SDF is associated with reduced chances of natural conception, increased failure rates of assisted reproduction, and miscarriages [10, 11].

DNA breaks occur both physiologically and pathologically during the development and maturation of sperm cells [12]. Several factors have been shown to be risk factors for a high SDF. However, the results range widely. For example, obesity was associated with an increase of 3.41% in SDF compared to normal body mass index (BMI), whereas the presence of varicocele was associated with a 9.84% increase [9, 13]. Identifying potentially modifiable risk factors for high SDF may ultimately lead to more satisfactory and cost-effective approaches to optimizing fertility, such as lifestyle modifications.

Therefore, we aimed to conduct a systematic review and meta-analysis on all risk factors that have been investigated as potentially increasing SDF.

## Methods

Our systematic review and meta-analysis are reported based on the recommendation of the PRISMA 2020 guideline (see Supplementary Table 1), and we followed the Cochrane Handbook [14, 15]. Furthermore, the study protocol was registered on PROSPERO with the registration number CRD42021282533, and we fully adhered to it.

### Eligibility criteria

We formulated our question using the PICO framework. Eligible studies included all male patients, regardless of their fertility status (P), and compared the SDF values (O) between groups with and without a particular risk factor (I and C). Risk factors included all lifestyle-, environmental-, and additional health factors. For the outcome measures, all sperm DNA fragmentation assays were included (e.g., sperm chromatin structure assay (SCSA), terminal deoxynucleotidyl transferase (dUTP), nick end labeling (TUNEL), sperm chromatin dispersion test (SCD), Comet assay, both neutral and alkaline). Eligible studies reported either the SDF difference between the groups with and without the risk factors in terms of mean difference (MD) or the rate of high SDF based on a specific cut-off value for each group. A change in SDF was considered clinically significant if it was around 10%, but SDF values were interpreted on a consensus basis, as there are no guideline recommendations that we could have followed.

Cohort studies of both prospective and retrospective designs were eligible. No studies were excluded on the basis of language criteria.

We excluded studies (1) with inaccurate data or if data were presented in a way that could not be further processed, (2) conference abstracts, (3) reviews, case series, and case reports.

### Information sources and search strategy

Our systematic search was conducted in Embase, MEDLINE (via PubMed), and Cochrane Central Register of Controlled Trials (CENTRAL) on October 17, 2021. During the systematic search, we used the following search key: (“sperm DNA fragmentation” OR “SDF” OR “DNA fragmentation index” OR “DFI”). We did not use filters or other restrictions.

### Selection process

Endnote v9.0 (Clarivate Analytics, Philadelphia, PA, USA) reference manager software was used for the selection. After automatic and manual duplicate removal, the selection was preformed in pairs by four independent review authors for the two halves of the articles (JÁ-AF and TF-AS) at the level of title, abstract, and full text level of the references. Disagreements were resolved at each level by a third review author for each group (NÁ and TS). The Cohen’s kappa coefficient (κ) was calculated after each step to measure interrater reliability [16].

### Data collection process and data items

Data from the eligible articles were collected by two authors (AS and JÁ) into a predefined data collection table. We extracted the following data: first author, year of publication, study design and period, number of participants and demographical data, fertility status, risk factors and groupings, SDF assay type, cut-off values for dichotomous outcomes, MD values with distribution of risk factor groups or high SDF in the risk factor groups, pregnancy or birth as additional outcomes in terms of either the risk factor or SDF, and information for assessing the risk of bias in the studies.

The original study investigator was contacted when data were missing or insufficient.

Wherever possible, we grouped participants in the selected studies by general population, fertility clinic, fertile, or a combination of these based on the fertility status of patients. Studies reporting similar SDF cut-off values were handled in the same group.

The preferred data format for SDF was the mean with standard deviation (SD). Therefore, data reported as median were approximated by the interquartile range to mean with SD based on the work by Wan et al. [17]. When we had more than one treatment group per study, we pooled these groups following the recommendation of the Cochrane handbook [15].

For sexual abstinence, we used as a reference the number of abstinence days recommended when examining standard semen parameters (2–7 days, 2–5 days, or 3–5 days). As these repeated measurement studies lacked SD of changes from baseline, we used a conservative approach and assumed a correlation of minus one to calculate the SD of changes, which is equivalent to the sum of the SDs.

### Study risk of bias assessment

Two review authors (SV and HP) performed the risk of bias assessment independently using the Quality in Prognostic Studies (QUIPS) tool [18]. Risk assessment categories were predetermined for each aspect (Supplementary Appendix). A third review author resolved disagreements (PN).

### Synthesis methods

All statistical analyses were performed with R (R Core Team 2022, v4.2) using the meta (v5.5.0) and dmetar (v0.0.9) packages [19].

The random-effects model was used with the inverse variance method for weighting and the Mantel–Haenszel method to pool odds ratio (OR) with a 95% confidence interval (CI) from 2 by 2 tables (risk factor yes/ no, SDF above and below cut-off) and models with restricted maximum likelihood method to calculate MD with 95%CI from mean values (mean SDF in groups with and without risk factor) [20].

Forest plots were used to graphically summarize the results irrespective of the number of studies included in the pooled analysis. Forest plots with fewer than three studies were interpreted with limitations.

Where applicable, we reported the prediction intervals (i.e., the expected range of effects of future studies) of results following the recommendations of IntHout et al. [21].

The Cochrane’s Q test was used to assess statistical heterogeneity with a *p*-value < 0.1 as a threshold for a statistically significant difference, and the I2 index was used to quantify between-study heterogeneity. In addition, Egger’s test and funnel plots were applied to report and visualize publication bias when at least ten studies were involved in the analysis.

Besides heterogeneity, a *p*-value < 0.05 was considered statistically significant.

A subgroup analysis was carried out based on the fertility status of the population and the SDF assay used.

## Results

### Search and selection

**Fig. 1:**
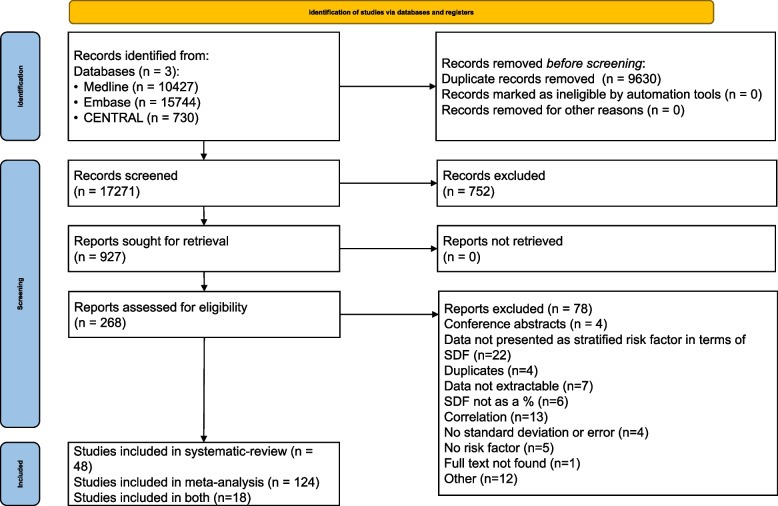
PRISMA 2020 flowchart showing the study selection process

Altogether, we found 26,901 articles using our search key, of which 190 were used for the meta-analysis or the systematic review (Fig. 1).

### Basic characteristics of included studies and summary of results

Baseline characteristics of the included analyses are detailed in Supplementary Table 2. The earliest studies were published in 2003, and the latest in 2021. The most common study location was Europe, closely followed by America and Asia. Fewer were from Africa, and the fewest studies were from Australia. Most articles were retrospective and mainly included male patients in their 30 s attending fertility clinics. The most frequently analyzed risk factor was the presence of varicocele, and the measurement of SDF was performed primarily via SCSA, SCD, or TUNEL assays. The eligibility criteria for each study are summarized in Supplementary Table 3. The studied risk factors of each article, risk factor definitions, and the definitions of fertility statuses are included in Supplementary Table 4. The main findings on pregnancies or births, and their connection to SDF are included in Supplementary Table 6.

### Risk factors – associated health conditions

We summarized risk factors with only one or two sources in Supplementary Figs. 46–48. Subgroups by fertility status and assay type used to measure SDF and results based on SDF cut-off values are summarized in the Supplementary material.

**Fig. 2:**
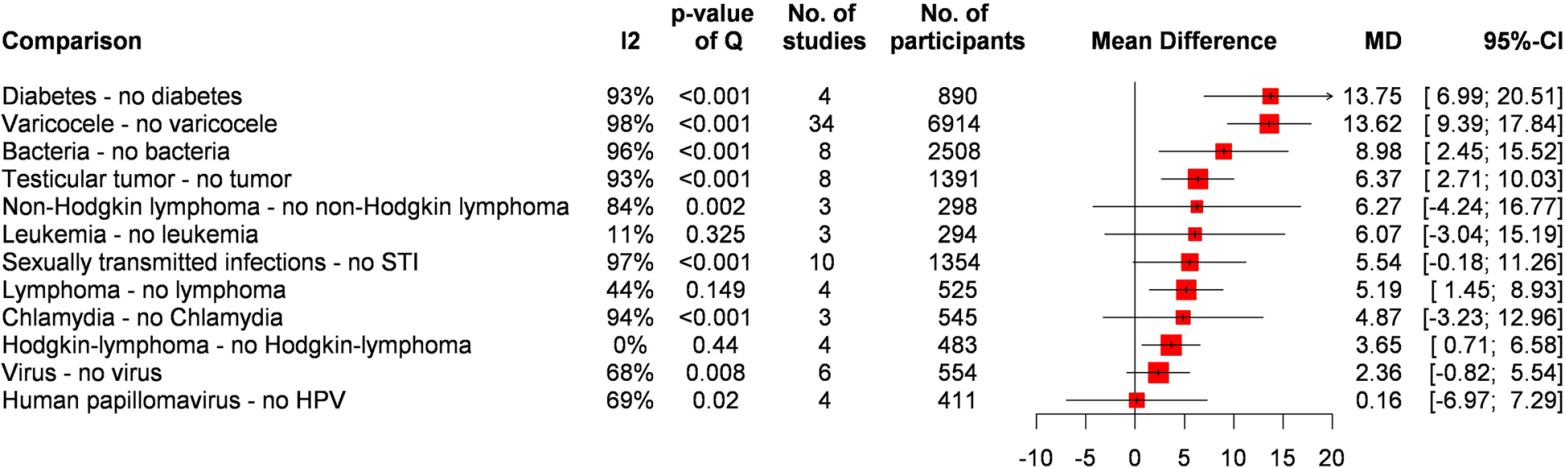
Summary forest plot of associated health conditions

Pooled SDF values for associated health conditions are summarized in Fig. 2 (see individual plots in the Supplementary Material). Regardless of the assay used, the presence of varicocele increased SDF by over 10% (MD = 13.62, CI: 9.39–17.84). The subgroup analysis by palpable and non-palpable varicocele yielded a smaller MD (= 7.95%, CI: 3.93–11.97) with a maximum of MD = 11.32% (CI: 3.47–19.17) when measured via SCSA.

Patients with impaired glucose tolerance showed a higher SDF than those with normal glucose tolerance (MD = 13.75, CI: 6.99–20.51).

For malignancies, testicular tumors had both statistically and clinically significant effects on SDF, with a maximum of MD = 11.3% (CI: 7.84–14.76) when measured by SCD. The presence of Hodgkin’s lymphoma was statistically significant but not clinically (MD = 3.65%, CI: 0.71–6.58). Lymphomas generally resulted in a higher SDF than those without (MD = 5.19%, CI: 1.45–8.93). The presence of non-Hodgkin’s lymphoma and leukemias did not reach statistical significance.

As for infections, the mean difference for *Chlamydia* was not significant, either statistically or clinically. The presence of human papillomavirus (HPV) did not result in a higher SDF either and in general, viral infections had a negligible effect (MD = 2.36, CI: -0.82–5.54). However, the presence of bacterial infections (MD = 8.98, CI: 2.45–15.52) or sexually transmitted infections (STIs) (MD = 5.54%, CI: -0.18–11.26) yielded ambiguous results.

### Risk factors – lifestyle factors

**Fig. 3:**
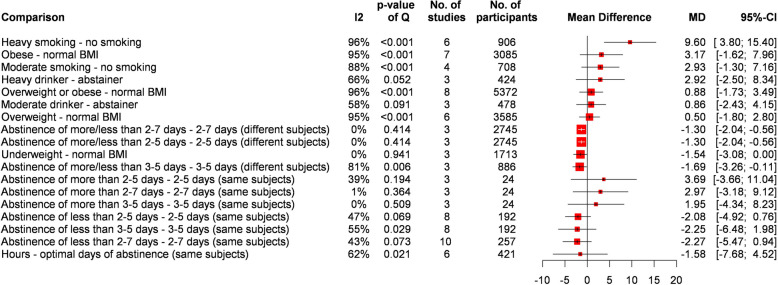
Summary forest plot of lifestyle factors

Results are summarized in Fig. 3. Smoking increased DFI (MD = 9.19%, CI: 4.33—14.06) compared to non-smokers. However, smoking showed a dose-dependency. In comparison to non-smokers, light smokers (MD = 2.93%, CI: -1.30–7.15) had a lower increase than heavy smokers (MD = 9.60%, CI: 3.80–15.40). Alcohol consumers had a higher SDF (MD = 1.88, CI: -1.93–5.69). However, the difference was clinically non-significant. On the other hand, comparing moderate (MD = 0.86%, CI: -2.43–4.15) and heavy drinkers (MD = 2.92%, CI: -2.51–8.34) to abstainers also resulted in a non-significant difference.

Overweight or obese patients had similar SDF values as those with normal BMI (MD = 0.88%, CI: -1.73–3.49). Underweight men showed a slightly lower SDF than patients with normal BMI (MD = -1.54, CI: -3.08–0.01). However, the difference was non-significant.

Abstinence for none of the generally recommended days seemed to result in clinically lower SDF compared to longer or shorter periods when results were compared for the same population or different patients.

### Risk factors – other risk factors

**Fig. 4:**
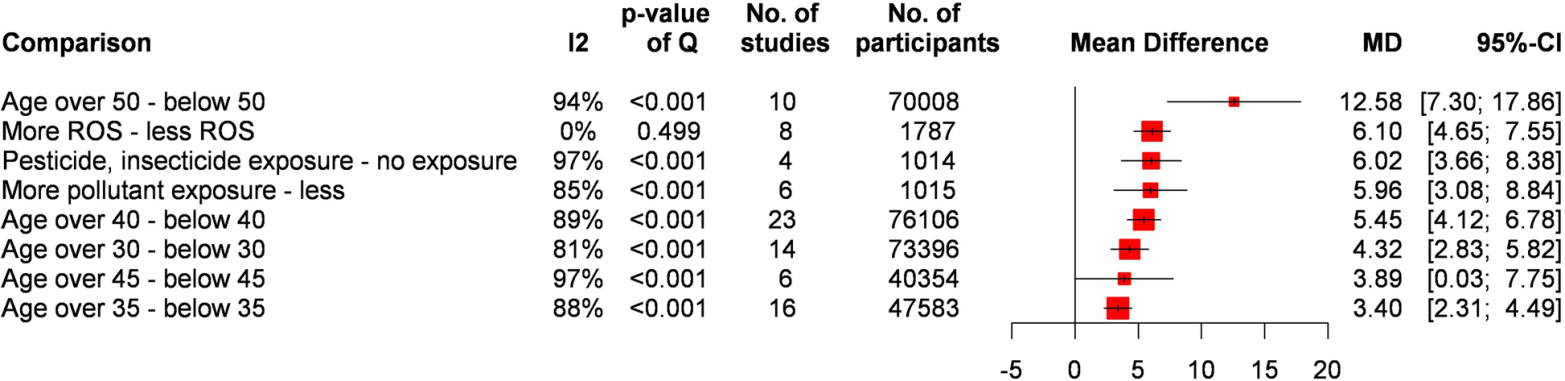
Summary forest plot of other factors

A summary of other risk factors is showing in Fig. 4. We compared multiple age groups to determine the optimal age for the lowest SDF. A clinically significant increase in SDF was seen in men over 50 (MD = 12.58%, CI: 7.31- 17.86) compared to those below.

Exposure to different pollutants significantly increased SDF (MD = 9.68%, CI: 6.85–12.52). However, the type of pollutants was heterogeneous. On the other hand, exposure to pesticides or insecticides significantly increased SDF (MD = 6.02%, CI: 3.66–8.38).

The pooled results of studies measuring reactive oxygen species (ROS) showed a higher SDF for higher ROS values (MD = 6.10%, CI: 4.65–7.55).

Risk factors with a more significant impact on SDF in the systematic review were spinal cord injuries in two studies (MD = 60.8%, CI: 53.94–67.66 and MD = 49.8%, CI: 35.66–63.94) and heroin use (MD = 31.79%, CI: 29.09–34.49). The effects of chronic prostatitis and previous orchidopexy also seem to be significant risk factors for urology, with a MD value of around 10% increase in SDF in the presence of the risk present.

### Risk of bias assessment

The results of the risk of bias assessment are presented in Supplementary Table 5. For study participation, the risk of bias was mainly low, whereas study attrition did not apply in most studies due to their retrospective nature. Next, we evaluated risk factor measurement, which was mainly of low risk of bias, similarly to outcome measurement. Finally, study confounding was of the highest risk of bias.

### Publication bias and heterogeneity

Egger’s test could only be performed for varicocele and age based on SDF cut-off values. Their p-values were 0.548 and 0.405, respectively.

Heterogeneity was high for almost every risk factor examined due to the heterogeneity in risk factor definitions.

## Discussion

This comprehensive meta-analysis provides a thorough summary of all potential risk factors of SDF that have been assessed to date. In addition, wherever possible, we performed subgroup analyses based on the severity or the quantity of exposure to the risk factor, the assay method used for measurement, and fertility status.

Although many studies have focused on the risk factors for standard semen parameters, much fewer data are available on the risk factors for SDF and even less, in turn, for pregnancy and birth outcomes in terms of SDF. Our study showed that several modifiable risk factors affect SDF notably. Amongst health conditions, varicocele and impaired glucose tolerance were found to have the strongest negative effect on SDF. With regard to infections, only a few studies are available that investigate the relationship between a specific pathogen and SDF. One of them was HPV, for which the literature is ambiguous, and the other one was *Chlamydia*, which had a higher effect on SDF [22]. Of malignancies, the presence of testicular tumors had the largest impact on SDF. Regardless of the type of malignancy, fertility counseling and preservation must always be offered prior to the initiation of treatment, as it may further reduce spermatogenesis or impede ejaculation, though it is yet again primarily based on information on standard parameters, as those on SDF are insufficient [23]. For these patients both non-surgical-, and if necessary, surgical options are available, such as testicular sperm extraction or epididymal sperm aspiration [24].

Among lifestyle factors, smoking had the greatest impact on SDF. It is known to affect health, including sexual health. Ramlau-Hansen et al. even showed a dose-dependency when investigating the effect of smoking on semen volume, concentration, and motility [25]. Several risk factors show a dose-dependency in our study, such as smoking, alcohol consumption, and BMI. Contrary to our expectations, based on the recommended, but not evidence-based short ejaculatory abstinence protocol supported by Gupta et al. based on standard semen parameters, different abstinence periods did not seem to alter SDF in our study significantly [26, 27]. Also, the effect of paternal age manifests later than expected based on the article by Matorras et al. at 35–39 years, which is above 50 according to our study [28].

Current guidelines suggest that 30–40% of infertility cases could be idiopathic in males, with a sole discrepancy in seminal parameters [6]. The underlying cause could be high sperm DNA fragmentation, which in turn could be caused by several mechanisms, including apoptosis, chromatin remodeling, the damage inflicted by ROS, endogenous enzymes, or exogenous factors, such as lifestyle and environmental factors exerting their effects through ROS [6, 29]. ROS are naturally produced in the body and are essential for several fertilization steps. However, overproduction and imbalance lead to oxidative stress and alterations of proteins, lipids, and DNA, especially in high concentrations, as sperm cells cannot prevent or repair the damage, although an egg cell can be of some assistance in the repair process [6, 30–33]. Thus, antioxidants have been extensively studied as a potential solution. Unfortunately, there is no evidence to support the use of antioxidants since the balance – both oxidative and reductive stress – should be considered, which cannot currently be measured correctly in clinical practice. Therefore, there is no clinically proven antioxidant regiment that could improve fertility [6]. Although the effect of various antioxidants could potentially be monitored via SDF measurements, more data are required.

Another issue concerning fertility is that there is no clinically defined “normal range” for SDF to distinguish between infertile and fertile men. Moreover, the only certain impact of a higher SDF is on miscarriage rates, no definite results are available on other aspects of pregnancies [34]. Currently, the preferred values of SDF are below 25%, but patients are still eligible for in vitro fertilization treatments with an SDF of up to 50% [35, 36]. One of the main reasons for the lack of reference ranges is the lack of a gold standard assay method. The most common include TUNEL and alkaline Comet assays, which determine DNA fragmentation directly, and SCD and SCSA used for the indirect measurement [6]. Another issue is the lack of inter-laboratory standards, which mean that every laboratory has to establish its own interpretations for its own assay. Thus, it is difficult to compare values between different laboratories [7].

Despite these shortcomings, other meta-analyses of SDF have been conducted, most commonly on varicocele. Varicocele is a common condition in approximately 15% of males and is thought to be a reversible cause of infertility when the correct indications for surgery are applied [37, 38]. It occurs in 19–81% of males, depending on whether infertility is primary or secondary, begging the question of its contribution to DNA fragmentation and, thus, infertility [39]. In a meta-analysis by Wang et al., the mean difference in SDF in males with and without varicocele turned out to be 9.84%, which is in line with our pooled results [9]. Only a handful of meta-analyses have examined the effect of a risk factor on SDF. Sepidarkish et al. compared BMI categories concerning SDF and had results that were supported by ours too. On the other hand, Gonzalez et al. reached the same conclusion as we did: namely that DNA fragmentation also increases with increasing paternal age, although they did not specify the age after which the deterioration is more severe [40, 41]. The review by Durairajanyagam et al. on lifestyle factors found similar factors contributing to infertility, but it did not expand on SDF in detail, instead it rather focused on standard semen parameters [42]. Contrary to our findings, Hanson et al. investigated the effect of different abstinence periods, but only had eight publications dealing with SDF. Half of them concluded that different abstinence period did not alter SDF, whereas others showed that the longer the abstinence period was, the worse the SDF values were. Therefore, confident conclusions could not be drawn from the review by Hanson et al. [27]. If we knew the best abstinence period, patients could single handedly influence their SDF and the goal for every risk factor would be to intervene so as to minimize the DNA fragmentation.

The best studied intervention is varicocelectomy, which, according to the meta-analysis by Qiu et al., reduces SDF by approximately 6% [43]. Another intervention that Maleki et al. found to improve DNA fragmentation, was high-intensity interval training, which improves SDF by approximately 15% in different groups compared to non-exercising controls [44]. Dietary modification is also often recommended after fertility assessment, but there is a lack of data to support such an intervention, especially for SDF [45].

The above examples show that we do not clearly understand several risk factors affecting sperm DNA fragmentation, let alone the possible means of reducing it. However, once we clarify the above, we can move on to the more important questions, i.e., their impact on pregnancy rates and live births.

### Strengths and limitation

Regarding the strengths of our analysis, we followed our protocol, which was registered in advance. A rigorous methodology was applied, and we included a large number of studies and a high number of patients, which resulted in the generalizability of our results. In addition, subgroup analyses led to more precise conclusions.

There are several limitations to our study. First, the included studies have different study designs, data collection methods, inclusion and exclusion criteria, definitions of fertility, and risk factors outcome measures. Many studies did not account for confounding factors. Lastly, the outcome measurements were also different between the studies. All this produced substantial heterogeneity.

## Conclusion

Our results suggest that several lifestyle-, environmental-, and additional health factors are associated with increased sperm DNA fragmentation.

### Implications for practice and research

On the basis of previous evidence, there are clear benefits of rapidly integrating results into clinical practice [46]. For patients with high SDF, specific treatment options and interventions should be sought based on the risk factors present. However, more research would be needed to clarify the direct effect of SDF on pregnancy outcomes. On the other hand, clear measurement protocols should be included in the guidelines.

## Supplementary Information

**Additional file 1: Supplementary Appendix 1.** Risk of bias assessment methodology. **Supplementary Table 1.** PRISMA 2020 checklist. **Supplementary Table 2.** Basic characteristics of the included article. **Supplementary Table 3.** Eligibility criteria in each included article. **Supplementary Table 4.** Risk factor and population definitions in each included article. **Supplementary Table 5.** Risk of bias assessment using the QUIPS tools. **Supplementary Table 6.** Articles also looking at pregnancy or birth as an outcome. **Supplementary Figure 1.** Comparison of patients’ sperm DNA fragmentation values with and without varicocele subdivided based on sperm DNA fragmentation assays used (continuous data). **Supplementary Figure 2.** Comparison of patients’ sperm DNA fragmentation values with and without varicocele subdivided based on different cut-off values. **Supplementary Figure 3.** **Supplementary Figure 4.** Comparison of patients’ sperm DNA fragmentation values with and without varicocele subdivided based on the fertility status of patients (continuous data). **Supplementary Figure 5.** Comparison of patients’ sperm DNA fragmentation values with impaired and normal glucose tolerance (continuous data). **Supplementary Figure 6.** Comparison of patients’ sperm DNA fragmentation values with and without testicular tumors subdivided based on sperm DNA fragmentation assays used (continuous data). **Supplementary Figure 7.** Comparison of patients’ sperm DNA fragmentation values with and without Hodgkin-lymphoma (HL) (continuous data). **Supplementary Figure 8.** Comparison of patients’ sperm DNA fragmentation values with and without non-Hodgkin lymphoma (NHL) (continuous data). **Supplementary Figure 9.** Comparison of patients’ sperm DNA fragmentation values with and without lymphomas (continuous data). **Supplementary Figure 10.** Comparison of patients’ sperm DNA fragmentation values with and without leukemia (continuous data). **Supplementary Figure 11.** Comparison of patients’ sperm DNA fragmentation values with and without human papilloma virus (HPV) infections (continuous data). **Supplementary Figure 12.** **Supplementary Figure 13.** Comparison of patients’ sperm DNA fragmentation values with and without viral infections (continuous data). **Supplementary Figure 14.** **Supplementary Figure 15.** **Supplementary Figure 16.** Comparison of smokers and non-smokers’ sperm DNA fragmentation values subdivided based on sperm DNA fragmentation assays used (continuous data). **Supplementary Figure 17.** Comparison of heavy smokers with non-smokers, and moderate smokers with non-smokers’ sperm DNA fragmentation values (continuous data). **Supplementary Figure 18.** Comparison of smokers and non-smokers’ sperm DNA fragmentation values subdivided based on the fertility status of patients (continuous data). **Supplementary Figure 19.** Comparison of drinkers’ and abstainers’ sperm DNA fragmentation values (continuous data). **Supplementary Figure 20.** Comparison of heavy drinkers with abstainers, and moderate drinkers with abstainers’ sperm DNA fragmentation values (continuous data). **Supplementary Figure 21.** Comparison of sperm DNA fragmentation values of patients with different BMI categories (continuous data). **Supplementary Figure 22.** **Supplementary Figure 23.** **Supplementary Figure 24.** **Supplementary Figure 25.** **Supplementary Figure 26.** **Supplementary Figure 27.** **Supplementary Figure 28.** **Supplementary Figure 29.** **Supplementary Figure 30.** **Supplementary Figure 31.** **Supplementary Figure 32.** **Supplementary Figure 33.** **Supplementary Figure 34.** **Supplementary Figure 35.** **Supplementary Figure 36.** Comparison of patients’ sperm DNA fragmentation values of different age groups (continuous data). **Supplementary Figure 37.** Comparison of patients’ sperm DNA fragmentation values of different age groups (continuous data). **Supplementary Figure 38.** Comparison of patients’ sperm DNA fragmentation values of different age groups with cut-off values being at 30% DNA fragmentation. **Supplementary Figure 39.** Comparison of patients’ sperm DNA fragmentation values of age groups over and below 40 years of age with different cut-off values for DNA fragmentation. **Supplementary Figure 40.** **Supplementary Figure 41.** Comparison of patients’ sperm DNA fragmentation values with exposures to different types of pollutants (continuous data). **Supplementary Figure 42.** **Supplementary Figure 43.** **Supplementary Figure 44.** Comparison of patients’ sperm DNA fragmentation values with more or less reactive oxygen species (ROS) (continuous data). **Supplementary Figure 45.** Comparison of patients’ sperm DNA fragmentation values with more vs. less reactive oxygen species (ROS) – reduced model (continuous data). **Supplementary Figure 46.** Comparison of patients’ sperm DNA fragmentation values within the risk group and control group (continuous data). **Supplementary Figure 47.** Comparison of patients’ sperm DNA fragmentation values within the risk group and control group (continuous data, same subjects in the two groups). **Supplementary Figure 48.** Comparison of patients’ sperm DNA fragmentation values within the risk group and control group (cut-off values).

## Data Availability

This study re-analyzed data published in the databases Embase, MEDLINE or Cochrane. The list of cited articles can be found in the manuscript or in the supplementary material.

## Abbreviations

BMI: Body mass index
CI: Confidence interval
DFI: DNA fragmentation index
dUTP: Deoxyuridine triphosphate
HPV: Human papilloma virus
MD: Mean difference
OR: Odds ratio
PICO: Population, intervention, comparison, outcome
QUIPS: Quality of prognostic studies
ROS: Reactive oxygen species
SCD: Sperm chromatin dispersion
SCSA: Sperm chromatin structure assay
SD: Standard deviation
SDF: Sperm DNA fragmentation
TUNEL: Terminal deoxynucleotidyl transferase dUTP nick end labeling
WHO: World health organization
