# Supplementary material for: Lifestyle-, environmental-, and additional health factors associated with an increased sperm DNA fragmentation: a systematic review and meta-analysis
Source: Reprod Biol Endocrinol. 2023 Jan 18;21:5. doi: 10.1186/s12958-023-01054-0 (PMC9847125; doi:10.1186/s12958-023-01054-0)
Supplement: Supplementary file 1 — Additional file 1: Supplementary Appendix 1. Risk of bias assessment methodology. Supplementary Table 1. PRISMA 2020 checklist. Supplementary Table 2. Basic characteristics of the included article. Supplementary Table 3. Eligibility criteria in each included article. Supplementary Table 4. Risk factor and population definitions in each included article. Supplementary Table 5. Risk of bias assessment using the QUIPS tools. Supplementary Table 6. Articles also looking at pregnancy or birth as an outcome. Supplementary Figure 1. Comparison of patients’ sperm DNA fragmentation values with and without varicocele subdivided based on sperm DNA fragmentation assays used (continuous data). Supplementary Figure 2. Comparison of patients’ sperm DNA fragmentation values with and without varicocele subdivided based on different cut-off values. Supplementary Figure 3. Supplementary Figure 4. Comparison of patients’ sperm DNA fragmentation values with and without varicocele subdivided based on the fertility status of patients (continuous data). Supplementary Figure 5. Comparison of patients’ sperm DNA fragmentation values with impaired and normal glucose tolerance (continuous data). Supplementary Figure 6. Comparison of patients’ sperm DNA fragmentation values with and without testicular tumors subdivided based on sperm DNA fragmentation assays used (continuous data). Supplementary Figure 7. Comparison of patients’ sperm DNA fragmentation values with and without Hodgkin-lymphoma (HL) (continuous data). Supplementary Figure 8. Comparison of patients’ sperm DNA fragmentation values with and without non-Hodgkin lymphoma (NHL) (continuous data). Supplementary Figure 9. Comparison of patients’ sperm DNA fragmentation values with and without lymphomas (continuous data). Supplementary Figure 10. Comparison of patients’ sperm DNA fragmentation values with and without leukemia (continuous data). Supplementary Figure 11. Comparison of patients’ sperm DNA fragmentation values with and withou [file 12958_2023_1054_MOESM1_ESM.zip › ESM1/DFI_risk_factors_supplementary_RB.docx]

**Supplementary Material**

**Title**

Lifestyle-, environmental-, and additional health factors associated with an increased sperm DNA fragmentation: a systematic review and meta-analysis

**Authors**

Anett Szabó^1,2^, Szilárd Váncsa^2,3,4^, Péter Hegyi^2,3,4^, Alex Váradi^2^, Attila Forintos^2^, Teodóra Filipov ^2,5^, Júlia Ács^1,2^, Nándor Ács^2,6^, Tibor Szarvas^1,2,7^, Péter Nyirády^1,2^, Zsolt Kopa^1,2^

**Affiliations:**

1. Department of Urology, Semmelweis University, Budapest, Hungary
2. Centre for Translational Medicine, Semmelweis University, Budapest, Hungary
3. Institute for Translational Medicine, Medical School, University of Pécs, Pécs, Hungary
4. Division of Pancreatic Diseases, Heart and Vascular Center, Semmelweis University, Budapest, Hungary
5. Department of Interventional Radiology, Semmelweis University, Budapest, Hungary
6. Department of Obstetrics and Gynecology, Semmelweis University, Budapest, Hungary
7. Department of Urology, University of Duisburg-Essen and German Cancer Consortium, Essen, Germany

**TABLE OF CONTENT**

**Supplementary Appendix 1.** Risk of bias assessment methodology

**Supplementary Table 1.** PRISMA 2020 checklist

**Supplementary Table 2.** Basic characteristics of the included article

**Supplementary Table 3.** Eligibility criteria in each included article

**Supplementary Table 4.** Risk factor and population definitions in each included article

**Supplementary Table 5.** Risk of bias assessment using the QUIPS tools

**Supplementary Table 6.** Articles also looking at pregnancy or birth as an outcome

**Supplementary Appendix 1.** Risk of bias assessment methodology

Overall ratings for each domain were assigned as carrying ‘low’ (green), ‘moderate’ (yellow) or ‘high’ (red) risk of bias, based on the items included in each domain.

**Study participation measurement:** (1) low risk of bias was attributed if authors adequately described the source population, including methods to identify patients and eligibility criteria. The description of the period and place of recruitment were also added to low risk of bias; (2) moderate risk of bias was attributed if a part of the above listed descriptions were missing; (3) high risk of bias was attributed if baseline characteristics, eligibility criteria, time and place of recruitment were not described. In all cases, the number of participants that could have been recruited was considered according to the risk factor studied.

**Study attrition assessment** was performed in the case of prospective studies: (1) low risk of bias was attributed if the proportion of baseline sample was available, also if the reason for lost to follow-up was detailed; (2) moderate risk of bias was attributed if a part of the above listed criteria were missing; (3) high risk of bias was attributed if data was missing for the abovementioned criteria. We did not assess attrition bias in retrospective studies (NA – not attributable).

**Prognostic factor measurement:** (1) low risk of bias was attributed, if the risk factor was measured/assessed/calculated with the most up-to-date method or via a detailed questionnaire e.g. varicocele determined via physical examination and ultrasonography. For risk factors such as age, we assumed that it was correctly calculated even if not detailed; (2) moderate risk of bias was attributed if the diagnosis was acquired from anamnesis or not the best method was used to assess the risk factor, but still, it was mentioned; (3) high risk of bias was attributed if the presence of a risk factor was only stated without any indication of measurement.

**Outcome measurement:** (1) low risk of bias was attributed if sperm DNA fragmentation was measured via SCSA, TUNEL, SCD or alkaline Comet assay; (2) moderate risk of bias was attributed if the neutral Comet assay or FPG-associated alkaline assay was used, Comet classes were given rather than percentage of DNA fragmentation or only the stain used was indicated, not the method itself; (3) high risk of bias was attributed if neither of the above were specified.

**Study confounding measurement:** (1) low risk of bias was attributed if important potential confounders were described and accounted for in the analysis, preferably in a table; (2) moderate risk of bias was attributed if some of the important confounders were described (at least 1-2 other possible risk factors); (3) high risk of bias was attributed if studies did not provide data on confounding factors.

**Statistical analysis measurement**: (1) low risk of bias was attributed if data was presented as mean and standard deviation; (2) moderate risk of bias was attributed if standard error or median with minimum and maximum values or quartiles were given; (3) high risk of bias was attributed if the data presented in the tables and figures did not match those described in the text.

**Supplementary Table 1.** PRISMA 2020 checklist

| **Section and topic** | **Item #** | **Checklist item** | **Location where item is reported** |  |
| --- | --- | --- | --- | --- |
| **Title** | | | | |
| Title | 1 | Identify the report as a systematic review. |  |  |
| **Abstract** | | | | |
| Abstract | 2 | See the PRISMA 2020 for Abstracts checklist (table 2). |  |  |
| **Introduction** | | | | |
| Rationale | 3 | Describe the rationale for the review in the context of existing knowledge. |  |  |
| Objectives | 4 | Provide an explicit statement of the objective(s) or question(s) the review addresses. |  |  |
| **Methods** | | | | |
| Eligibility criteria | 5 | Specify the inclusion and exclusion criteria for the review and how studies were grouped for the syntheses. |  |  |
| Information sources | 6 | Specify all databases, registers, websites, organizations, reference lists and other sources searched or consulted to identify studies. Specify the date when each source was last searched or consulted. |  |  |
| Search strategy | 7 | Present the full search strategies for all databases, registers, and websites, including any filters and limits used. |  |  |
| Selection process | 8 | Specify the methods used to decide whether a study met the inclusion criteria of the review, including how many reviewers screened each record and each report retrieved, whether they worked independently, and if applicable, details of automation tools used in the process. |  |  |
| Data collection process | 9 | Specify the methods used to collect data from reports, including how many reviewers collected data from each report, whether they worked independently, any processes for obtaining or confirming data from study investigators, and if applicable, details of automation tools used in the process. |  |  |
| Data items | 10a | List and define all outcomes for which data were sought. Specify whether all results that were compatible with each outcome domain in each study were sought (e.g., for all measures, time points, analyses), and if not, the methods used to decide which results to collect. |  |  |
|  | 10b | List and define all other variables for which data were sought (e.g., participant and intervention characteristics, funding sources). Describe any assumptions made about any missing or unclear information. |  |  |
| Study risk of bias assessment | 11 | Specify the methods used to assess risk of bias in the included studies, including details of the tool(s) used, how many reviewers assessed each study and whether they worked independently, and if applicable, details of automation tools used in the process. |  |  |
| Effect measures | 12 | Specify for each outcome the effect measure(s) (e.g., risk ratio, mean difference) used in the synthesis or presentation of results. |  |  |
| Synthesis methods | 13a | Describe the processes used to decide which studies were eligible for each synthesis (e.g., tabulating the study intervention characteristics and comparing against the planned groups for each synthesis (item #5)). |  |  |
|  | 13b | Describe any methods required to prepare the data for presentation or synthesis, such as handling of missing summary statistics, or data conversions. |  |  |
|  | 13c | Describe any methods used to tabulate or visually display results of individual studies and syntheses. |  |  |
|  | 13d | Describe any methods used to synthesize results and provide a rationale for the choice(s). If meta-analysis was performed, describe the model(s), method(s) to identify the presence and extent of statistical heterogeneity, and software package(s) used. |  |  |
|  | 13e | Describe any methods used to explore possible causes of heterogeneity among study results (e.g. subgroup analysis, meta-regression). |  |  |
|  | 13f | Describe any sensitivity analyses conducted to assess robustness of the synthesized results. |  |  |
| Reporting bias assessment | 14 | Describe any methods used to assess risk of bias due to missing results in a synthesis (arising from reporting biases). |  |  |
| Certainty assessment | 15 | Describe any methods used to assess certainty (or confidence) in the body of evidence for an outcome. |  |  |
| **Results** | | | | |
| Study selection | 16a | Describe the results of the search and selection process, from the number of records identified in the search to the number of studies included in the review, ideally using a flow diagram (see fig 1). |  |  |
|  | 16b | Cite studies that might appear to meet the inclusion criteria, but which were excluded, and explain why they were excluded. |  |  |
| Study characteristics | 17 | Cite each included study and present its characteristics. |  |  |
| Risk of bias in studies | 18 | Present assessments of risk of bias for each included study. |  |  |
| Results of individual studies | 19 | For all outcomes, present, for each study: (a) summary statistics for each group (where appropriate) and (b) an effect estimate and its precision (e.g. confidence/credible interval), ideally using structured tables or plots. |  |  |
| Results of syntheses | 20a | For each synthesis, briefly summarize the characteristics and risk of bias among contributing studies. |  |  |
|  | 20b | Present results of all statistical syntheses conducted. If meta-analysis was done, present for each the summary estimate and its precision (e.g. confidence/credible interval) and measures of statistical heterogeneity. If comparing groups, describe the direction of the effect. |  |  |
|  | 20c | Present results of all investigations of possible causes of heterogeneity among study results. |  |  |
|  | 20d | Present results of all sensitivity analyses conducted to assess the robustness of the synthesized results. |  |  |
| Reporting biases | 21 | Present assessments of risk of bias due to missing results (arising from reporting biases) for each synthesis assessed. |  |  |
| Certainty of evidence | 22 | Present assessments of certainty (or confidence) in the body of evidence for each outcome assessed. |  |  |
| **Discussion** | | | | |
| Discussion | 23a | Provide a general interpretation of the results in the context of other evidence. |  |  |
|  | 23b | Discuss any limitations of the evidence included in the review. |  |  |
|  | 23c | Discuss any limitations of the review processes used. |  |  |
|  | 23d | Discuss implications of the results for practice, policy, and future research. |  |  |
| **Other information** | | | | |
| Registration and protocol | 24a | Provide registration information for the review, including register name and registration number, or state that the review was not registered. |  |  |
|  | 24b | Indicate where the review protocol can be accessed, or state that a protocol was not prepared. |  |  |
|  | 24c | Describe and explain any amendments to information provided at registration or in the protocol. |  |  |
| Support | 25 | Describe sources of financial or non-financial support for the review, and the role of the funders or sponsors in the review. |  |  |
| Competing interests | 26 | Declare any competing interests of review authors. |  |  |
| Availability of data, code, and other materials | 27 | Report which of the following are publicly available and where they can be found: template data collection forms; data extracted from included studies; data used for all analyses; analytic code; any other materials used in the review. |  |  |

**Supplementary Table 2.** Basic characteristics of the included articles

| **Author (year)** | **Study site** | **Study type** | **Population** | **Number of analyzed patients** | **Age (year) ‡** | **Risk factors** | **DFI measurement** |
| --- | --- | --- | --- | --- | --- | --- | --- |
| Abdelbaki (2017)(1) | Egypt | p | fertility clinic + fertile controls | 80 | 31.5 (23-49) | varicocele | SCSA |
| Abdullah (2019)(2) | USA | r | varicocele | 141 | 34.2 ± 6.2 | testicular atrophy | SCD |
| Agarwal (2016)(3) | USA | p | fertile | 7 | ND (20-45) | abstinence | TUNEL |
| Agbaje (2008)(4) | UK | p | diabetes type 1 patients + fertile controls | 19 | 32.6 ±1.5 | diabetes type 1 | alkaline Comet |
| Alargkof (2019)(5) | Bulgaria | r | fertility clinic + general population controls | 28 | 33 (26.25-42) | varicocele | SCD |
| Albani (2019)(6) | Italy | r | fertility clinic | 89 | 37.9 ± 3.5 | age | SCSA |
| Alhathal (2016)(7) | Canada | p | fertility clinic + general population controls | 35 | ND | varicocele | SCSA |
| Alshahrani (2014)(8) | USA | r | fertility clinic | 472 | ND | age | TUNEL |
| Ammar (2021)(9) | Tunisia | p | fertility clinic + fertile controls | 80 | ND | varicocele | TUNEL |
| Amor (2019)(10) | Germany | r | fertility clinic | 141 | ND | smoking | TUNEL |
| Andersen (2016)(11) | Norway | r | fertility clinic + general population controls | 112 | 36.5 (22-61) | body mass index | SCSA |
| Anifandis (2014)(12) | Germany | p | fertility clinic | 207 | 37.43 ± 4.3 | smoking, alcohol | SCD |
| Ayad (2018)(13) | South Africa | r | fertile | 20 | ND | abstinence | TUNEL |
| Bandel (2015)(14) | Sweden, Greenland, Ukraine, Poland, Norway | r | general population (mainly fertile) | 1503 | 27.9 ± 10.9 | body mass index | SCSA |
| Banks (2021)(15) | USA | p | fertility clinic | 135 | 35 (32-40) | vitamin-D | SCSA |
| Belloc (2009)(16) | France | r | fertility clinic | 1111 | ND | age | TUNEL |
| Berg (2021)(17) | Germany | p | patients with chronic prostatitis + general population controls | 63 | 35 (20-62) | prostatitis, age | SCSA |
| Bergamo (2016)(18) | Italy | r | general population | 40 | 28 ± 6 | pollution | SCD |
| Bian (2004)(19) | China | r | Fenvalerate (pesticide) exposed workers, office workers of factory, unexposed controls (general population) | 63 | 30.2 ± 8 | fenvalerate (pesticide) | alkaline Comet, TUNEL |
| Boeri (2020)(20) | Italy | r | fertility clinic | 1547 | 37 (18-60) | age, body mass index | SCSA |
| Boeri (2019)(21) | Italy | r | fertility clinic | 189 | 38.1 ± 5.6 | smoking, alcohol | SCSA |
| Boeri (2019)(22) | Italy | r | fertility clinic | 744 | 38 (19-50) | pre-diabetes | SCSA |
| Bojar (2013)(23) | Poland | r | fertility clinic | 185 | ND | age, smoking | SCSA |
| Borges (2019)(24) | Brazil | p | fertility clinic | 463 | 38.28 ± 5.74 | abstinence | SCD |
| Bosco (2018)(25) | Italy | r | general population | 175 | 36.5 ± 4.8 | pollution | SCD |
| Bozhedomov (2021)(26) | Russia | p | fertility clinic | 1502 | 32.5 ± 5.6 | varicocele | SCD |
| Brackett (2008)(27) | Florida | r | spinal cord injury (SCI) & non-SCI controls | 22 | 33.3 ± 9.8 | abstinence, spinal cord injury | SCSA |
| Brahem (2011)(28) | Tunisia | p | fertility clinic | 140 | ND (24-76) | age | TUNEL |
| Chavarro (2010)(29) | USA | r | fertility clinic | 483 | 36.3 ± 5.4 | body mass index | neutral Comet |
| Chigrinets (2019)(30) | Russia | r | fertility clinic | 34 | 30.8 ± 3.9 | smoking, alcohol | SCD |
| Comar (2017)(31) | Brazil | r | fertility clinic | 2458 | 38 ± 6.7 | abstinence | TUNEL |
| Cortés-Gutiérrez (2017)(32) | Mexico | r | human papilloma virus infected + fertile controls + fertility clinic controls | 38 | 29 (19-32) | human papilloma virus | SCD |
| Cui (2016)(33) | China | r | fertility clinic | 1128 | ND | smoking | acridine orange staining |
| Dahan (2020)(34) | Canada | p | fertility clinic | 112 | 41.1 ± 6.3 | abstinence | SCD |
| Darbandi (2019)(35) | Iran | p | fertility clinic | 151 | 34.5 ± 2 | reactive oxygen species | SCD |
| Darbandi (2019)(36) | Iran | r | fertility clinic | 70 | ND | age | SCD |
| Das (2013)(37) | Canada | r | fertile | 148 | ND (20-57) | age | SCSA |
| De Jonge (2004)(38) | Belgium | p | fertility clinic | 11 | 30 ± 2.9 | abstinence | SCSA |
| De Win (2021)(39) | Belgium | p | general population | 89 | 21.7 ± ND | varicocele | TUNEL |
| Dehghan Marvast (2018)(40) | Iran | r | fertility clinic | 80 | 33 (22-49) | Chlamydia trachomatis | aniline blue, chromomycin A3, TUNEL, acridine orange |
| Depuydt (2021)(41) | Belgium | p | fertility clinic | 180 | 34.9 ± ND | human papilloma virus | SCSA |
| Dieamant (2017)(42) | Brazil | r | fertility clinic | 2399 | 37.8 ± 6.5 | varicocele | TUNEL |
| Domes (2012)(43) | Canada | r | fertility clinic | 1806 | 37.7 ± 6.1 | bacteriospermia, elevated seminal leukocytes | "the DNA fragmentation assay" |
| Dupont (2013)(44) | France | r | fertility clinic | 331 | 37.6 ± 6.2 | body mass index | TUNEL |
| Eini (2021)(45) | Iran | r | fertility clinic + fertile controls | 207 | 35 ± 20 | bacteriospermia | SCD |
| Eisenberg (2014)(46) | USA | p | general population | 459 | 31.8 ± 4.8 | body mass index, waist circumference, sports | SCSA |
| Elbardisi (2021)(47) | Qatar | r | fertility clinic | 269 | 34 (ND) | age | SCD |
| Elbardisi (2020)(48) | Qatar | r | fertility clinic | 1068 | 35.98 ± 7.78 | oxidation-reduction potential | SCD |
| Elbardisi (2018)(49) | Qatar | r | fertility clinic | 1050 | 36 ± 0.1 | geography, age | SCD |
| Elshal (2009)(50) | Egypt | r | general population | 86 | ND | smoking | SCSA |
| Esfaahani (2010)(51) | Iran | r | varicocele patients + fertile controls | 122 | ND | varicocele | SCD |
| Esteves (2015)(52) | Brazil | r | fertility clinic +fertile controls | 289 | ND | varicocele, testicular cc, Chlamydia | SCD |
| Evenson (2020)(53) | North America, Europe | r | fertility clinic, general population | 25445 | ND (21-80) | age | SCSA |
| Falahieh (2021)(54) | Iran | p | fertile | 20 | ND (20-50) | COVID | SCD |
| Fernandez-Encinas (2020)(55) | Spain | r | fertility clinic + fertile donors | 24 | ND | varicocele | alkaline Comet, neutral Comet, SCD |
| Finelli (2021)(56) | Italy | r | varicocele patients + fertile controls | 169 | 30.6 ± 8 | varicocele | TUNEL |
| Frainais (2010)(57) | France | r | fertility clinic | 40 | 40 ± 5.85 | human immunodeficiency virus | TUNEL |
| Gallegos (2008)(58) | Mexico | p | fertility clinic + fertile controls | 193 | ND (25-51) | Chlamydia trachomatis + Mycoplasma urealyticum | SCD |
| Gao (2020)(59) | China | r | fertility clinic | 102 | 32 (20-49) | Location of semen collection | TUNEL |
| Gao (2021)(60) | China | r | fertility clinic | 18441 | 34 ± 5.66 | age | SCSA |
| García-Ferreyra (2015)(61) | Peru | r | fertility clinic | 32 | ND (34-72) | age | SCD |
| García-Peiró (2011)(62) | Spain | r | fertility clinic + fertile controls | 19 | ND | varicocele | SCD |
| García-Peiró (2014)(63) | Spain | r | fertility clinic + fertile controls | 52 | ND (25-35) | varicocele | TUNEL, SCD, SCSA |
| Gautam (2015)(64) | India | r | general population | 26 | 29 ± 4.9 | smoking, alcohol | SCSA |
| Ghandehari-Alavijeh (2019)(65) | Iran | r | fertility clinic + fertile controls | 40 | ND | varicocele | TUNEL |
| Ghazavi-Khorasgani (2017)(66) | Iran | r | fertility clinic + fertile controls | 55 | ND | varicocele | SCSA |
| Gill (2019)(67) | Poland | r | general population | 254 | 32.6 ± 5.8 | low activity at work | SCD |
| Gill (2020)(68) | Poland | r | fertility clinic | 675 | 32 (19-54) | age | SCD |
| Gill (2021)(69) | Poland | r | fertility clinic + fertile controls + healthy controls | 335 | ND | varicocele | SCD |
| Giwercman (2007)(70) | Greenland, Sweden, Poland, Ukraine | r | mainly fertile (general population) | 680 | 34 ± 10 | geography | SCSA |
| Gosálvez (2011)(71) | Spain | p | fertile | 21 | ND (25-35) | abstinence | SCD |
| Grosen (2021)(72) | Denmark | p | Methotrexate-treated + general population | 54 | ND (18-45) | Methotrexate, inflammatory bowel disease/rheumatoid arthritis/psoriatic arthritis | SCSA |
| Grosen (2019)(73) | Denmark | p | inflammatory bowel disease patients | 56 | 29 (18-50) | inflammatory bowel disease | SCSA, neutral Comet |
| Grosen (2019)(74) | Denmark | r | inflammatory bowel disease patients + general population | 55 | 28 (18-46) | inflammatory bowel disease, Vendolizumab | SCSA, neutral Comet |
| Grosen (2019)(75) | Denmark | p | inflammatory bowel disease patients + general population | 80 | 25 (18-45) | inflammatory bowel disease, Thiopurines | SCSA, neutral Comet |
| Guo (2020)(76) | China | r | fertility clinic (abnormal sperm parameters), fertility clinic (normozoospermia) | 429 | ND | age | SCD |
| Håkonsen (2012)(77) | Denmark | r | general population | 337 | ND (18-21) | body mass index, smoking, abstinence | SCSA |
| Hammadeh (2010)(78) | Germany | r | fertility clinic | 116 | 37.9 ± 5.7 | smoking | TUNEL |
| Hammiche (2011)(79) | Netherlands | p | fertility clinic | 227 | 36.9 (25.8-59.1) | geography | SCSA |
| Hansen (2012)(80) | Denmark | r | general population | 345 | ND (18-21) | alcohol in last 5 days | SCSA |
| Henkel (2003)(81) | Germany | r | fertility clinic | 44 | ND | reactive oxygen species | TUNEL |
| Homa (2019)(82) | UK | r | fertility clinic | 238 | 38.3 ± ND | seminal oxidative stress | SCSA |
| Horta (2011)(83) | Chile | r | general population | 62 | 35 ± ND | age | TUNEL, SCD |
| Huang (2011)(84) | Taiwan | r | general population (workers of PVC pellet manufacturing plant) | 45 | 35.2 ± 9.2 | di(2-ethylhexyl) phthalate (DEHP) exposure | SCSA |
| Humaidan (2021)(85) | Denmark | p | fertility clinic + general population controls | 86 | 35 (24-55) | seminal oxidative stress | SCSA |
| Iommiello (2015)(86) | Italy | r | fertility clinic | 56 | ND | ejaculate oxidative stress | SCSA |
| Janghorban-Laricheh (2016)(87) | Iran | r | fertility clinic + fertile | 55 | ND | varicocele | SCSA |
| Jeng (2015)(88) | USA | r | general population (coke oven workers + administrators/security) | 177 | 40 ± 10 | Polycylic aromatic hydrocarbons | TUNEL, SCSA |
| Jeremias (2021)(89) | Brazil | r | fertility clinic | 94 | 34 ± 5.5 | varicocele | alkaline Comet, neutral Comet, FPG-associated alkaline assay |
| Ji (2011)(90) | China | r | fertility clinic | 240 | 28.5 ± 3.6 | 3-phenoxybenzoic acid (3-PBA) | TUNEL |
| Ji (2013)(91) | China | r | fertility clinic | 433 | 28.4 ± 3.3 | Polycylic aromatic hydrocarbons | TUNEL |
| Jurewicz (2018)(92) | Poland | r | fertility clinic | 336 | 32 ± ND | diet | SCSA |
| Kabukçu (2021)(93) | Turkey | p | fertility clinic | 106 | 33 ± 4.8 | abstinence | TUNEL |
| Karimi (2012)(94) | Iran | r | diabetes patients + fertile (non-diabetic) | 67 | 33 ± 7 | diabetes | TUNEL |
| Kaspersen (2013)(95) | Denmark | r | general population | 76 | 24 ± ND | Human herpes virus or human papilloma virus | SCSA |
| Kavoussi (2021)(96) | USA | r | patients with varicocele | 141 | 34.2 ± 6.2 | testicular atrophy | SCD |
| Kiwitt-Cárdenas (2021)(97) | Spain | r | general population (healthy university students) | 158 | 20.5 (18-23) | bisphenol A | SCD |
| Krüger (2008)(98) | Denmark | r | fertile | 300 | ND | geography | SCSA |
| Kumar (2013)(99) | India | r | general population (hospital workers: occupationally exposed to ionizing radiation and not) | 134 | ND (21-50) | ionizing radiation | TUNEL, alkaline Comet, SCSA |
| Kumar (2015)(100) | India | r | fertile | 130 | ND | smoking | SCSA |
| La Vignera (2012)(101) | Italy | r | fertility clinic + fertile controls | 60 | 26.5 ± 3.2 | varicocele | TUNEL |
| Laqqan (2021)(102) | Palestine | r | general population | 188 | 34.9 ± 5.8 | smoking | TUNEL |
| Lara-Cerrillo (2020)(103) | Spain | r | fertility clinic + fertile | 32 | ND (17-44) | varicocele | alkaline Comet, neutral Comet |
| Le (2020)(104) | Vietnam | r | fertility clinic | 290 | 35.26 ± 5.87 | body mass index, metabolic syndrome | SCD |
| Le (2021)(105) | Vietnam | r | fertility clinic | 534 | 34.7 ± 6.3 | metabolic syndrome | SCD |
| Lenters (2015)(106) | Greenland, Poland, Ukraine | r | fertile | 602 | 30 ± ND | geography | TUNEL, SCSA |
| Li (2012)(107) | Japan | r | fertility clinic + fertile controls | 38 | 33.1 ± ND | varicocele | SCSA |
| Liu (2021)(108) | China | r | fertility clinic | 253 | 31 ± 2.3 | Chlamydia trachomatis + Ureaplasma urealyticum | SCD |
| Liu (2021)(109) | China | r | fertility clinic | 108 | 33.6 ± 10.1 | oxidative stress | SCD |
| Long (2007)(110) | Greenland, Poland , Ukraine, Sweden (Denmark) | r | fertile | 262 | ND (18-68) | geography | TUNEL |
| Lu (2018)(111) | China | p | fertility clinic | 1010 | 28.89 ± ND | body mass index, waist circumference, waist-to-hip ratio, waist-to-height | SCSA |
| Lu (2020)(112) | China | r | fertility clinic, fertility clinic (normozoospermic) | 1790 | ND (21-58) | age | SCSA |
| Lu (2017)(113) | China | r | diabetic patients + general population | 60 | ND (21-49) | diabetes | acridine orange |
| Ma (2017)(114) | China | r | fertility clinic + fertile | 49 | ND (24-42) | Ureaplasma urealyticum | TUNEL |
| Mahfouz (2010)(115) | USA | p | fertility clinic | 101 | 37 ± 7.5 | seminal reactive oxygen species | TUNEL |
| Mahran (2019)(116) | Egypt | r | fertility clinic + fertile | 110 | 28.5 ± 5.5 | varicocele | acridine orange |
| Malm (2017)(117) | Sweden | r | general population | 198 | 28,5 ± ND | geography, season (melatonin) | SCSA |
| Manna (2020)(118) | Italy | p | fertile, fertility clinic | 30, 35 | 37 ± 3.3 | abstinence | SCD |
| Marchlewska (2016)(119) | Poland | r | fertility clinic + patients with germ cell testicular tumor | 335 | 34 (24-58) | testicular cancer | SCD |
| Martínez (2021)(120) | Argentina | r | fertility clinic | 163 | ND (28-55) | age | TUNEL |
| Mayorgaa-Torres (2015)(121) | Colombia | r | general population | 6 | 26.7 ± 4.8 | abstinence | SCSA |
| Mayorgaa-Torres (2016)(122) | Colombia | r | general population | 3 | 32 ± 3.6 | abstinence | SCSA |
| McDowell (2013)(123) | Australia | r | cancer pts + fertile controls | 124 | 31.6 ± ND | tumors | SCSA |
| Meseguer (2008)(124) | Spain | r | cancer patients + sperm donors + fertile + infertile patients | 291 | ND | tumors | SCD |
| Mohammed (2015)(125) | Egypt | p | fertility clinic + fertile controls | 115 | 30.7 ± ND | varicocele | acridine orange, SCSA |
| Moskovtsev (2009)(126) | Canada | r | fertility clinic | 84 | ND | varicocele, bacteriospermia | acridine orange |
| Moskovtsev (2009)(127) | Canada | r | fertility clinic | 2586 | ND | age | SCSA |
| Moustafa (2004)(128) | USA | r | fertility clinic + general population | 27 | ND | reactive oxygen species | SCSA |
| Nazmara (2021)(129) | Iran | r | general population (but half are heroin users) | 48 | 34.8 ± 1.5 | heroin | SCSA |
| Nguyen (2019)(130) | Vietnam | p | fertility clinic + fertile controls | 358 | ND | varicocele | SCD |
| Ni (2016)(131) | China | r | fertility clinic + fertile controls | 113 | 29 ± 3.2 | varicocele | SCSA |
| Nijs (2011)(132) | Belgium | p | fertility clinic | 278 | 35.1 ± 4.9 | age | SCSA |
| Oliveira (2014)(133) | Brazil | r | fertility clinic | 1500 | 37.7 ± 6.7 | age | TUNEL |
| Oliveira (2018)(134) | Brazil | r | fertility clinic | 1824 | 37.9 ± 6.6 | body mass index | TUNEL |
| Osadchuk (2014)(135) | Russia | r | general population | 44 | 30.5 ± 0.9 | varicocele, prostatitis | SCSA |
| Pant (2014)(136) | India | r | general population | 278 | 28.5 ± 4.2 | lindane, p-p′-DDE | SCSA |
| Pearce (2019)(137) | Australia | r | fertility clinic | 29 | 36.9 ± 5.2 | body mass index | SCD |
| Pelliccione (2011)(138) | Italy | p | sportsmen | 7 | 33 ± 9 | body mass index, waist circumference, % body fat | TUNEL |
| Petersen (2018)(139) | Brazil | p | fertility clinic | 2178 | 37.9 ± 6.4 | age | TUNEL |
| Pons (2013)(140) | Spain | p | fertility clinic (DFI>=30) | 35 | 38 ± 5.5 | abstinence | SCD |
| Rago (2013)(141) | Italy | r | fertile | 63 | 28.5 ± 5 | cell phone use | TUNEL |
| Ranganathan (2019)(142) | India | p | fertile, fertility clinic | 170, 170 | 30 ± 3.5 | smoking | diphenylamine method (colorimetric method, aniline blue) |
| Ribeiro (2008)(143) | Brazil | p | fertility clinic + fertile controls | 98 | 27 ± 5.2 | tumors | TUNEL |
| Romerius (2010)(144) | Sweden, Norway | r | general population, childhood cancer survivors given radiotherapy | 292, 12 | 29 (19-46) | childhood cancer and treatment | SCSA |
| Rosiak-Gill (2019)(145) | Poland | r | fertility clinic + general population, fertile | 336, 160 | 33 (ND) | age | TUNEL |
| Rubes (2010)(146) | Czech Republic | r | general population | 47 | 33.6 ± 5.3 | smoking, pollution | SCSA |
| Rubes (2021)(147) | Czech Republic | r | general population | 150 | 37.8 ± 9.1 | age | SCSA |
| Safarinejad (2008)(148) | Iran | r | fertile | 118 | 34.5 ± ND | selective serotonin reuptake inhibitors | SCSA |
| Safarinejad (2010)(149) | Iran | r | mustard gas injured and not, fertile and infertile | 268 | 47.2 ± ND | mustard gas | SCSA |
| Said (2009)(150) | Canada | r | general population + fertile controls | 109 | 31 ± ND | tumors | SCSA |
| Saleh (2003)(151) | USA | p | fertility clinic + fertile controls | 47 | 32 (ND) | varicocele | SCSA |
| Savasi (2018)(152) | Italy | p | HIV patients | 77 | 41 (25-54) | highly active antiretroviral therapy, age | SCD |
| Sepaniak (2006)(153) | France | p | fertility clinic | 108 | 30.5 ± 6.8 | smoking | TUNEL |
| Smit (2010)(154) | Netherlands | p | general population (cancer patients) + fertile controls | 149 | ND | tumors and treatment | SCSA |
| Smit (2010)(155) | Netherlands | p | vasectomy reversal patients + fertile controls | 92 | 41.6 (ND) | vasectomy reversal | SCSA |
| Smith (2007)(156) | Chile | r | orchidopexy patients + idiopathic oligozoospermic controls + normozoospermic healthy controls | 63 | ND | orchidopexy | SCSA, TUNEL |
| Smith (2006)(157) | Chile | r | fertility clinic + fertile controls | 80 | 28.2 ± 4.5 | varicocele | SCSA, TUNEL |
| Spanò (2005)(158) | Italy | r | general population (mainly fertile) | 707 | 31 (18-67.5) | pollution | SCSA |
| Specht (2012)(159) | Denmark | r | fertile | 548 | ND (18-51) | pollution | SCSA, TUNEL |
| Ståhl (2009)(160) | Sweden | r | general population (cancer patients) + fertile controls | 258 | 29 (16-56) | tumors and treatment | SCSA |
| Ståhl (2004)(161) | Sweden | p | testicular germ cell tumor patients + general population controls | 362 | 29 (ND) | tumor treatment | SCSA |
| Ståhl (2006)(162) | Sweden | r | testicular germ cell tumor patients + general population controls | 355 | 30 (range=49) | tumor treatment | SCSA, TUNEL |
| Stronati (2006)(163) | Italy | r | mainly infertile | 652 | ND | pollution | TUNEL |
| Taha (2012)(164) | Egypt | r | fertile | 160 | ND | smoking | SCSA |
| Taha (2014)(165) | Egypt | r | fertility clinic | 246 | 35 ± 4 | smoking, varicocele | SCSA |
| Taha (2019)(166) | Egypt | r | fertile, hepatitis B surface antigen seropositive fertile men | 103 | ND | hepatitis B virus | SCD |
| Taha (2016)(167) | Egypt | r | fertile | 165 | 36.5 ± 4.9 | body mass index | SCSA |
| Tahamtan (2019)(168) | Iran | r | fertility clinic | 38 | ND | varicocele | TUNEL |
| Talebi (2008)(169) | Iran | p | fertility clinic + fertile controls | 60 | ND | varicocele | anilin blue, chromomycin A3, acridine orange, toluidine blue |
| Tanaka (2020)(170) | Japan | p | fertility clinic + fertile controls | 240 | ND | varicocele | SCSA |
| Tangal (2019)(171) | Turkey | p | fertility clinic | 117 | 36 ± 5.4 | human papilloma virus | TUNEL |
| Tartibian (2012)(172) | Iran | p | general population (athletes and recreationally active) | 108 | ND (18-28) | sports | TUNEL |
| Vagnini (2007)(173) | Brasil | r | fertility clinic | 508 | 37.7 ± 6.2 | age | TUNEL |
| van Brakel (2017)(174) | Netherlands | r | fertility clinic (follow-up on fertility) + fertile controls | 121 | ND | undescended testes | SCSA |
| Vargas-Baquero (2020)(175) | Spain | r | general population (spinal cord injury patients) + fertile controls | 37 | 32.2 ± 9 | spinal cord injury | SCD |
| Vaughan (2020)(176) | Spain | r | fertility clinic | 16 945 | 37.6 ± 6.8 | age | SCSA |
| Vellani (2013)(177) | Italy | r | fertility clinic | 179 | 38.91 ± 4.54 | anxiety from in vitro fertilization | TUNEL |
| Vinnakota (2019)(178) | New Zealand | r | fertility clinic + fertile controls | 1219 | 41 ± 4 | age | SCSA |
| Vivas-Acevedo (2014)(179) | Venezuela | p | fertility clinic + fertile controls | 90 | ND | varicocele | SCD |
| Vujkovic (2009)(180) | Netherlands | p | fertility clinic | 161 v 252 | 36 (28.5-53.9) | diet | SCSA |
| Wang (2018)(181) | China | r | general population | 707 | 20 (ND) | sleep | SCSA |
| Wang (2012)(182) | China | r | fertility clinic + fertile controls | 76 | 30.2 ± 5.6 | varicocele | SCD |
| Wijesekara (2020)(183) | Sri Lanka | r | fertility clinic | 40 | 34.8 ± 5.34 | lead exposure | SCD |
| Winkle (2008)(184) | Germany | r | fertility clinic, fertile | 320, 84 | 36.62 ± ND | age | SCSA |
| Wyrobek (2006)(185) | USA | r | general population | 88 | 41.5 (20-80) | age | SCSA |
| Yang (2016)(186) | China | r | fertility clinic | 104 | 34.4 ± 4 | body mass index | SCD |
| Zequiraj (2019)(187) | Kosovo | r | general population | 169 | ND | age | SCD |
| Zeyad (2018)(188) | Germany | r | fertility clinic | 120 | ND | bacteriospermia | TUNEL |
| Zhang (2021)(189) | China | r | fertility clinic | 5114 | 32 (ND) | age | SCSA |
| Zhu (2021)(190) | China | r | fertility clinic | 54 | ND (22-40) | body mass index | SCSA |

‡ parameters represented as mean with standard deviation, or median with range (minimum and maximum)

SCSA: sperm chromatin structure assay, TUNEL: terminal deoxynucleotidyl transferase (dUTP) nick end labeling, SCD: sperm chromatin dispersion test

ND: not defined

**Supplementary Table 3.** Eligibility criteria in each included article

| **Author (year)** | **Inclusion criteria (“verbatim”)** | **Exclusion criteria (“verbatim”)** |
| --- | --- | --- |
| Abdelbaki (2017)(1) | Minimum 1 year infertility. Abnormal seminal variables (reduced sperm concentration, motility and morphology on two or more semen samples) associated with unilateral or bilateral clinical varicocele (Grade 1–3). A control group, comprised of 20 normozoospermic healthy fertile men with normal standard semen variables according to WHO criteria | Patients with subclinical varicocele, azoospermia, systemic or endocrine disease, male accessory gland infection, cryptorchidism, testicular atrophy, cigarette smoking, alcohol or drug abuse, or recent hormonal treatment for fertility were excluded from the study. Other exclusion criteria were patients with all other factors that could affect sperm DNA fragmentation (SDF) such as obesity, patients with leukocytospermia or exposed to gonadotoxins, radiochemotherapy, and patients with cancer. |
| Abdullah (2019)(2) | Varicocele | Excluded were men who underwent bilateral varicocele repair, with bilateral testicular atrophy, who had testicular atrophy and a varicocele with a history of cryptorchidism, who had undergone varicocele repair for hypogonadism for orchialgia and not for fertility, and who did not obtain a 3-month postoperative semen analysis because they achieved a pregnancy before then or who did not follow up. |
| Agarwal (2016)(3) | The inclusion criteria were semen analysis results within the WHO (2010) reference ranges (sperm concentration >15x106/mL, motility ≥40%, and leukocytospermia ≤1x106/mL) during the initial screening obtained after 3-7 days of abstinence. | None. |
| Agbaje (2008)(4) | Male type 1 diabetics aged between 18 and 60 years attending the Regional Centre for Endocrinology and Diabetes, the Royal Victoria Hospital, Belfast, for routine clinical review of their diabetes were invited to participate in this study (n = 11). Control non-diabetic subjects of proven recent fertility (n = 12) aged between 18 and 60 years were recruited from men attending with their partners for a standard antenatal visit at the Royal Jubilee Maternity Hospital, Belfast, at between 12 and 14 weeks of pregnancy. | None. |
| Alargkof (2019)(5) | Control group of men with unknown fertility, normal genital examination and normal bulk semen parameters (n = 10) and two groups of patients with clinically diagnosed varicocele and infertility. | None. |
| Albani (2019)(6) | None. | None. |
| Alhathal (2016)(7) | Men presenting to our clinic with one year or more of infertility, a clinically palpable varicocele and abnormal semen parameters | Men with azoospermia, severe oligozoospermia (<5 million spermatozoa/mL), complete asthenozoospermia, or evidence of genital tract infection were excluded. |
| Alshahrani (2014)(8) | Minimum 1 year infertility. | None. |
| Ammar (2021)(9) | Minimum 1 year infertility | Patients with evidence of established cause of female partner infertility, azoospermia, oligozoospermia, necrozoospermia, secondary infertility, urogenital infections, hypogonadism (testicular volume < 15 ml), chemotherapy or radiotherapy history, chronic health problem, or medication |
| Amor (2019)(10) | None | None |
| Andersen (2016)(11) | Age 18 and above, general population through advertisements. The others were from couples with known female infertility factors and unknown semen quality, attending a fertility clinic aged ≥35 | None |
| Anifandis (2014)(12) | None | None |
| Ayad (2018)(13) | None | None |
| Bandel (2015)(14) | Cohort A: Men who underwent the examination from May to December 2000 and living within a 60 km radius from the city of Malmo¨ were invited to participate in the study.  Cohort B: All 1681 men who underwent the examination, lived within a 60 km radius of the city of Malmo¨ , and were born of and raised by mothers born and raised in Sweden, were asked to participate, and 241 (14%) accepted. Another 73 men of the same age were recruited through advertisement in schools, or as friends of participants.  Cohort C: Between May 2002 and February 2004, spouses of pregnant women were recruited in different regions of Greenland (n ¼ 200), in Kharkiv, Ukraine (n ¼ 207) and in Warsaw, Poland (n ¼ 143). Recruitment was when the pregnancy was recorded at the participating hospitals. Additionally, fishermen from the west and east coast of Sweden, regardless of fertility status, were included (n ¼ 184), constituting the final 734. The participants’ ages ranged from 18 to 69 years.  Cohort D: Eligible were men aged 19–40 years, who had lived in the area for at least 1 year and who would remain in the area for at least another year. | with serious chronic disease were excluded |
| Banks (2021)(15) | Men (n=154) with sperm concentration between 5 and 15 million/mL, motility<=40%, or normal morphology<=4% were eligible. Female partners were ovulatory, <=40 years old, and had documented tubal patency. | None |
| Belloc (2009)(16) | None | None |
| Berg (2021)(17) | The control group consisted of 22 healthy volunteers above 18 years of age without preexisting urological conditions | Exclusion criteria comprised lower urinary tract symptoms (LUTS) with the benign prostate syndrome (BPS), prostate cancer and chronic epididymitis/orchitis. Bacterial infection was excluded. |
| Bergamo (2016)(18) | Residence for at least 10 years in the study area, no known chronic diseases (diabetes or other systemic diseases), no varicocele, no prostatitis and other factors that could affect semen quality (such as fever, medications, exposure to X rays etc.), no reported history of drug abuse and no known occupational exposures to toxic chemicals. | None |
| Bian (2004)(19) | Controls were selected from the same district and were matched on socioeconomic class. | Past medical history (liver or renal disease, long term psychotropic drug therapy, significant x-ray exposure, or recent exposure to other neurotoxic drugs). |
| Boeri (2020)(20) | ≥18 and ≤60 years old and had male factor infertility only | Men with genetic abnormalities (any type) were excluded from final analysis; likewise, patients who had testicular or pituitary surgery and/or previous vasectomy and men who were on pharmacological agents (any) that could affect total testosterone values (i.e., clomiphene citrate) at the time of investigation were removed from the final analysis. (all white European) |
| Boeri (2019)(21) | Infertile patients were included in the study if they had only male factor infertility (Caucasian-European men). | None |
| Boeri (2019)(22) | White-European men (age range 18–50 years), only male factor infertility | Exclusion criteria were, men with (i) testicular factors previously associated with infertility (e.g., cryptorchidism; Grade III and IV varicocele; disturbance of erection/ ejaculation); (ii) genetic abnormalities (any type); (iii) modifiable hypothalamic and/or pituitary defects; (iv) testicular or pituitary surgery and/or previous vasectomy; (v) previous diagnosis of tumours; and, (vi) either previous or current testosterone therapy. |
| Bojar (2013)(23) | The patients had no history of other diseases which could disturb fertility and semen parameters. | Patients qualified for in vitro fertilization were also excluded from the study. |
| Borges (2019)(24) | couples with isolated male infertility undergoing their first ICSI cycle, with fresh embryo transfer performed on day 5 of development, were included | Couples undergoing ICSI with vitrified/thawed or donated oocytes, surgical sperm retrieval, vitrified/thawed embryo transfer, donated embryos, or preimplantation genetic diagnosis or screening, as well as couples with female infertility, were excluded from the analysis. |
| Bosco (2018)(25) | None | No known chronic diseases (diabetes or other systemic diseases), no varicocele, no prostatitis and other factors that could affect semen quality (such as fever, medications, exposure to X rays etc.), no reported history of drug abuse and no known occupational exposures (except group A) to toxic chemicals. |
| Bozhedomov (2021)(26) | Men aged 18-45, patients with or without and grade of varicocele based on physical examination. Anamnestic data on the duration of infertility, the presence or absence of pregnancy from the male partner in the past, including the past 3 months, regular unprotected sexual intercourses at least once a week. | The lack of required clinical or laboratory parameters, sperm concentration of less than 1 million/ml, high risk of genetic anomalies. |
| Brackett (2008)(27) | injured at least 1 year before the start of the study. Control subjects were age matched, non-SCI, healthy, normospermic men with no history of infertility. No SCI subject or control subject had taken any medications known to affect semen quality within 6 months of the start of the study. | None |
| Brahem (2011)(28) | None | None |
| Chavarro (2010)(29) | None | Men presenting for post-vasectomy semen analysis were not invited to participate. |
| Chigrinets (2019)(30) | Men aged 22-40.  Infertility of unknown origin with patozoospermia  Reproductive losses of the couple  Planned pregnancy with normozoospermia  Sperm donors  Signed informed consent to be included in the study | Varicocele  Genitourinary tract infections  Leukocytospermia  MAP-test (mixed antiglobulin test)>10%  Azoospermia  Hypogonadism and other endocrine diseases  Oncological diseases regardless of localization  Systemic illnesses  Unwilling to sign consent form to be included in the study |
| Comar (2017)(31) | None | Exclusion criteria were azospermia, any known reproductive tract pathology in the last six months, any hormonal therapy in the past six months and chronic medical disorders. |
| Cortés-Gutiérrez (2017)(32) | Unable to conceive naturally for a period of at least 1 year. | Couples with significant female factor infertility (e.g., endometriosis, tubal obstruction or ovulatory anomalies) were excluded from the study. |
| Cui (2016)(33) | Being the male partner of an infertile couple for a duration of at least 1 year, having regular intercourse, and seeking infertility treatment at the Reproductive Medicine Center, Shanxi Women and Infants Hospital over the study period. | abnormalities of the external genitalia and cryptorchidism; ultrasonographic examination was performed to exclude varicoceles; microbiological examination and spermioculture were performed to exclude infections; an immunobead binding test was performed to exclude the presence of anti-sperm antibodies; karyotyping was used to exclude any chromosomal abnormality; and genetic examination was performed to exclude Y chromosome microdeletions and cystic fibrosis gene mutations. |
| Dahan (2020)(34) | None | azoospermia or severe oligospermia preventing the use of the DNA fragmentation test or inability to produce a second semen analysis 3 h after the first ejaculation. |
| Darbandi (2019)(35) | None | None of the subjects had any history of pelvic and genital infections, chronic diseases (diabetes, cancer, etc.), endocrine abnormalities, chromosomal aberrations, azoospermia, and leukocytospermia. Participants were nonsmokers and had not taken any medication with potential gonadotoxic effects within at least 3 months before enrollment. |
| Darbandi (2019)(36) | None | None |
| Das (2013)(37) | non-azoospermic | None |
| De Jonge (2004)(38) | None of the men reported fever or other illness during the 8 weeks before and during the study | None |
| De Win (2021)(39) | Young volunteers between 16 and 26 years, Tanner 5, were recruited. | Participants with inguinoscrotal, urogenital or general diseases other than varicoceles that could affect testicular growth in childhood or puberty were excluded. |
| Dehghan Marvast (2018)(40) | None | one or both of them had (i) abnormal karyotype; (ii) history of chemotherapy or radiotherapy treatment; (iii) previous sterilization; (iv) low semen volume (<1.0 ml) or retrograde ejaculation in the male partner; (v) hypogonadotropic hypogonadism; (vi) a genital tract anomaly. |
| Depuydt (2021)(41) | Included couples failed to conceive for at least 12 months. | None |
| Dieamant (2017)(42) | All men from the couples who underwent infertility investigation between 2010 and 2015 were included | Azoospermia, any known reproductive tract disease or hormonal therapy, chronic medical disorders, and antioxidant intake. |
| Domes (2012)(43) | None | There were no exclusion criteria for patients included in the study, as long as there was an antegrade ejaculate sample that did not demonstrate azoospermia on semen analysis. |
| Dupont (2013)(44) | None | None. |
| Eini (2021)(45) | None | None. |
| Eisenberg (2014)(46) | Female age 18–44 years and male age 18+ years; in a committed relationship; ability to communicate in English or Spanish; menstrual cycles between 21 and 42 days; no hormonal contraception injections during past year; and no sterilization procedures or physician diagnosed infertility. | None |
| Elbardisi (2021)(47) | At least 1 year of infertility. Females with age <36 years. Normal ovarian reserve (AMH ≥15.7 pmol/L). Normal transvaginal US. Patent fallopian tube. Males with normal semen analysis (WHO fifth edition, 2010) done on two separate occasions. ICSI cycles with fresh ejaculated sperm. | H/O chemotherapy, radiotherapy, drug addiction or occupational exposure. Wife age ≥36 years. Decrease ovarian reserve. Presence of hydrosalpinx, endometriosis, PCOD, thin endometrium (<7 mm) or any gynaecological problem. Abnormal semen report. ICSI cycle with frozen or surgically retrieved sperms. |
| Elbardisi (2020)(48) | None | Patients receiving antioxidant, hormonal therapy or antibiotics, as well as patients with testicular malignancy, receiving chemotherapy or radiotherapy were excluded from this study. |
| Elbardisi (2018)(49) | None | Repeated patients who came for follow-up, and patients who received treatment prior to their semen analysis (e.g., antioxidants, empiric medical therapy, and surgical treatments including varicocelectomy or seminal tract reconstruction) were excluded. |
| Elshal (2009)(50) | Inclusion criteria in infertile group included infertility after at least 1 year of unprotected intercourse. Fertile strict non-smoker men (n=16) who have fathered a child within the previous 6 months were taken as a control group. Strict nonsmokers were those men who had never smoked before. | The exclusion criteria include leukocytospermia, frank pyospermia, haemospermia or chronic urinary tract infection; history of intake of non-proprietary medications or tonics; azoospermia; and age above 45 years, to avoid effects of ageing on sperm variables, history of tobacco smoking (for the infertile strict non-smokers group), to avoid any persistence of effects of smoking; alcohol intake, occupational exposure to chemicals or excessive heat; history of injury to the testes, varicocele, hydrocoele, undescended testis or its corrective surgery and vasectomy-reversal surgery; history of any chronic illness, such as tuberculosis, diabetes mellitus, hypertension, thyroid diseases, mumps or any ailment for which long-term medication was being given. |
| Esfaahani (2010)(51) | Grade 2 or 3 for varicocele. Individuals without varicocele, as the control group. Individuals included in this study did not have a history of systemic illnesses, cryptorchidism, orchitis, epididymitis, urethritis or testicular atrophy. Fertile controls' wives were pregnant at the time of study. | None |
| Esteves (2015)(52) | None | None |
| Evenson (2020)(53) | None | None |
| Falahieh (2021)(54) | In all, 63 men aged between 20 and 50 years who were hospitalized due to a moderate COVID-19 infection were eligible for inclusion in the study. All patients included in the study tested positive for SARS-CoV-2 infection following quantitative real-time polymerase chain reaction (PCR) analysis of pharyngeal–nose swabs. Thereafter, the infection was confirmed by chest computed tomography (CT). 20 fertile men with proven fertility (whose wives gave birth to a healthy child within the previous 2 years) were selected for the study. | The exclusion criteria were a history of varicocele, cryptorchidism, congenital disorders, immunological and inflammatory diseases, hormone disruptions, diabetes, alcohol abuse and/or cigarette smoking, a history of any scrotal or inguinal surgery, malignant disease, the consumption of any drug affecting the male reproductive tract and a high-risk job environment (e.g. exposure to certain chemicals, pesticides, herbicides, organic solvents, painting materials, radiation, extreme heat etc.). |
| Fernandez-Encinas (2020)(55) | The inclusion criteria for the study enrollment were to belong to one of the clinical groups defined for the study: Fertile donors, recurrent miscarriage patients with at least 2 miscarriages, and infertile patients without or with varicocele. | No exclusive criteria were defined regarding smoking, drinking, the use of recreational drugs, etc. |
| Finelli (2021)(56) | None | Exclusion criteria for patients and controls were any medical/surgical treatment in the 3 months before the study, previous history of radio/chemotherapy, abnormal male karyotype, and the presence of any other condition that could interfere with semen analyses and cytokines’ assessment (fever, any endocrine disease, leukocytospermia and urogenital infections, neoplasia, etc.) |
| Frainais (2010)(57) | For both groups, alcohol abuse was stopped at least 2 years before inclusion and sperm was bacteria-free. Sperm samples for HIV-1-infected patients were collected after at least 70 days of spermatogenesis (after two cycles of spermatogenesis). | None |
| Gallegos (2008)(58) | None | None |
| Gao (2020)(59) | History of infertility for at least 1 year were recruited. from the andrology clinic | The exclusion criteria were as follows: (a) presence of dysuria, urinary urgency and increased frequency of urination; (b) erectile or ejaculatory dysfunction; (c) inability to follow instructions due to impaired cognition; and (d) being recruited into other research projects. |
| Gao (2021)(60) | None | None |
| García-Ferreyra (2015)(61) | None | None |
| García-Peiró (2011)(62) | None | None |
| García-Peiró (2014)(63) | None | Patients with genitourinary inflammation, leukocytospermia, or altered hormonal profiles were excluded from the study. |
| Gautam (2015)(64) | None | None |
| Ghandehari-Alavijeh (2019)(65) | None | Infertile men with grade I varicocele, recurrent varicocele, leukocytospermia, azoospermia, seminal sperm antibodies, fever within 90 days prior to the seminal analysis, abnormal hormonal profile, anatomical disorders, Klinefelter’s syndrome, cancer, urogenital infections, previous history of scrotal trauma or surgery were excluded from this study. In addition, men with abnormal status of excessive alcohol consumption or special drug consumption and/or occupational exposure to heat were excluded from this study. |
| Ghazavi-Khorasgani (2017)(66) | Group 1: infertile males with group 2 or 3 varicocele. Group 2: fertile males with no varicocele | None |
| Gill (2019)(67) | None | Exclusion criteria included the following: working time less than 35 h per week, a clinical picture suggestive of obstructive azoospermia, a history of testicular torsion, varicocele, maldescent of testis, cryptorchidism, injury or cancer and co-existing systemic disease. |
| Gill (2020)(68) | None | The main exclusion criteria in this study were age <18 years, azoospermia, a history of testicular torsion or injury, and a history of radiotherapy, chemotherapy, or chronic illness, and long-term exposure to medicaments as well as drugs. |
| Gill (2021)(69) | None | None |
| Giwercman (2007)(70) | Partners of pregnant women. Swedish men who were recruited from a previously established population of fishermen, independent of fertility. Seventy-nine percent of the Swedish fishermen, however, had fathered a child. The populations were selected to represent different exposure scenarios of relatively high POP exposure in the populations from Greenland and Sweden, with high consumption of POP-contaminated seafood. The Ukrainian population was expected to be exposed to POPs mainly from previous agricultural use of organochlorine pesticides, whereas the exposure level in Poland was expected to be in the lower range and similar to other European populations without specific exposure sources. 18 years old or above. | None |
| Gosálvez (2011)(71) | Adverse female factor, normozoospermic man.  Selected donors. (other experiment) | None |
| Grosen (2021)(72) | Adult male patients between the ages of 18 and 45 years diagnosed with either Crohn’s disease (CD), ulcerative colitis (UC), rheumatoid arthritis (RA), or psoriatic arthritis (PsA) were recruited from the outpatient clinics. Inclusion criteria were initiation or cessation of methotrexate therapy. Further, patients on maintenance methotrexate therapy for a minimum of 4 months were enrolled in the cross-sectional study. | Exclusion criteria were concurrent infectious disease with fever within the last 3 months, documented infertility, vasectomy, or treatment with cyclophosphamide, sulfasalazine, leflunomide, chloroquines, azathioprine/6-mercaptopurine, abatacept, tocilizumab, rituximab, anakinra, canakinimab, tofacitinib, ustekinumab, secukinumab, or apremilast within the last 3 months. |
| Grosen (2019)(73) | Male patients, 18–50 years of age, with an established diagnosis of CD or ulcerative colitis [UC]. We enrolled three patient groups: patients with severe active disease who required admission to intravenous corticosteroid treatment, patients seen in our outpatient clinic who initiated infliximab or adalimumab, and patients who stopped infliximab or adalimumab therapy. The 20 patients admitted with severe disease were treated with intravenous methylprednisolone 40 mg twice a day. | Exclusion criteria were vasectomy, known infertility, previous chemotherapy, and treatment with drugs known to affect male fertility [e.g., sulphasalazine], treatment with vedolizumab, ustekinumab, or tofacitinib, or the use of recreational drugs [e.g., marijuana]. |
| Grosen (2019)(74) | None | None |
| Grosen (2019)(75) | Inclusion criteria were maintenance therapy with AZA or 6-MP during at least the previous 3 months, corresponding to the length of spermatogenesis.15 Moreover, patients had to be in clinical remission for at least 3 consecutive months before sampling. Concomitant treatments with 5-aminosalicylic acid [5-ASA] or anti-tumour necrosis factor-alpha [TNF-alpha] inhibitors were permitted. | None |
| Guo (2020)(76) | None | None |
| Håkonsen (2012)(77) | None | Men with severe handicaps or syndromes, such as spastic paraplegia or Down’s syndrome, as well as men with metabolic diseases or psychiatric disorders, were not invited. |
| Hammadeh (2010)(78) | None | Patients were also asked about any history of genital or urinary tract infection or if they had such a history of other conditions such as injury to testes, varicocele, hydrocele, undescended testis or its corrective surgery, vasectomy-reversal surgery or history of any chronic illness such as tuberculosis and mumps or pyospermia, haemospermia or chronic urinary tract infection. These individuals and those with a history of prolonged medication, intake of indigenous medications, consumption of alcohol and occupational exposures to known reproductive toxins or excessive heat were excluded. In addition, samples with a sperm concentration of less than 10 × 106 ml were excluded because they offered insufficient material. |
| Hammiche (2011)(79) | For this study men were included only if sperm was cryopreserved or obtained by microsurgical or percutaneous epididymal sperm aspiration. For the present analyses we included only men with information on ethnic background and availability of 1 sperm analysis (n=175). | None |
| Hansen (2012)(80) | None | Men with severe handicaps or congenital syndromes as well as men with a psychiatric or metabolic disorder were not invited to participate. The regional research ethic committee approved the study (registration number 20040174 and 20090203) and participation was made conditional on written informed consent. |
| Henkel (2003)(81) | None | None |
| Homa (2019)(82) | None | Exclusion criteria were incomplete sample collection, febrile illness during the previous 12 weeks, both of which may have affected the reliability of the results, and samples containing less than 1 million/mL sperm as ROS and sORP measurement are inaccurate and unreliable when the sperm concentration falls below this value. |
| Horta (2011)(83) | None | All subjects with concentration <=5 million sperm per ml (severe oligozoospermia) and a seminal volume less than 0,5 ml were excluded. |
| Huang (2011)(84) | None | Above 60 years of age, three non–on-site workers were excluded as well. |
| Humaidan (2021)(85) | Participants were non-smokers, including no intake of cannabis, and were not taking medication with known gonadotoxic effects when entering the IVF program. Moreover, at the first consultation, all patients were advised by the treating physician to reduce exposure to pesticides, irradiation, and excess heat in daily life and work. Furthermore, patients with sperm concentrations below 5 million/mL in raw semen had a testicular ultrasound to exclude malignancies. Only patients who had at least two failed IVF/ICSI trials for no apparent reasons, resulting in either embryo developmental arrest, implantation failure, biochemical pregnancy, or miscarriage were invited to participate. | None |
| Iommiello (2015)(86) | None | Samples with severe dispermy (sperm concentration minor of 2×106/mL) were not included; a minimum amount of sperm to reliably perform the various sperm assays is required. |
| Janghorban-Laricheh (2016)(87) | Male factor infertility presenting primary infertility with grade II and III unilateral varicocele. | Leukocytospermia, azoospermia, seminal sperm antibodies, fever within 90 days prior to the seminal analysis, abnormal hormonal profile, anatomical disorders, testicular size discrepancy, Klinefelter’s syndrome, cancer, grade I varicocele, recurrent varicocele, urogenital infections, previous history of scrotal trauma or surgery, excessive alcohol, drug consumption, and occupational exposure to heat were excluded from this study. |
| Jeng (2015)(88) | Criteria for human subject selection included being a male between 25 and 50 years old, having no reproductive dysfunction, and being employed at the plant more than one year. We recruited non-smokers only because tobacco smoke contains PAHs and other chemicals that could induce oxidative damage. | None |
| Jeremias (2021)(89) | Men aged between 18 and 50 years old, whose semen presented volume ≥1.5mL, concentration ≥15x106 sperm/mL, sperm progressive motility ≥32% and sperm morphology ≥4% of normal cells. | Obesity (body mass index - BMI ≥30.0), smoking habits or use of psychotropic substances, report of fever within 90 days prior to semen collection, medical history of alterations associated with the urogenital tract, of systemic diseases such as cancer, or of chemotherapy or radiotherapy treatments. For the control group (n=39), only individuals without varicocele were included, whereas for the varicocele group (n=55), only men with varicocele grades II or III, uni- or bilaterally, were included. |
| Ji (2011)(90) | Men with wives not diagnosed as infertile were recruited. | Men with abnormal sexual and ejaculatory functions, immune infertility, semen non-liquefaction (a liquefaction process taking more than 30 min), medical history of risk factors for infertility (e.g. varicocele, post-vasectomy or orchidopexy) and receiving treatment for infertility (e.g. hormonal treatments) were excluded. Men with other known factors related to male infertility, such as genetic disease, infection, occupational exposure to polyaromatic hydrocarbons (PAHs) or other agents suspected to be associated with male reproduction, were also excluded. We also excluded subjects with Y-chromosome microdeletions of the azoospermia factor region. |
| Ji (2013)(91) | None | Men with abnormal sexual and ejaculatory functions, immune infertility, semen non-liquefaction, medical history of risk factors for infertility (e.g., varicocele, post-vasectomy or orchidopexy) and receiving treatment for infertility (e.g., hormonal treatments) were excluded from the study. Men with other known factors related to male infertility, such as genetic disease, infection, occupational exposure to PAHs or other agents suspected to be associated with male reproduction were also excluded. Furthermore, to avoid spermatogenesis impairment caused by Y chromosome microdeletions, we excluded subjects with Y chromosome microdeletions of azoospermia factor region. |
| Jurewicz (2018)(92) | Normal semen concentration of 20 to 300 mln/ml or slight oligozoospermia (semen concentration of 15-20 mln/ml). | None |
| Kabukçu (2021)(93) | Infertility for more than 12 months, female age between 20 and 40 years, regular menstrual cycles, midluteal progesterone levels of > 3 ng/ml, basal FSH < 12 mIU/ ml, AMH > 1 ng/ml, body mass index (BMI) 19–35 kg/m2, no pelvic pathology documented by transvaginal ultrasound and bilateral tubal patency diagnosed by hysterosalpingography, normal semen parameters according to WHO criteria [18]. Patients were excluded from the study if they had any endocrine and pelvic pathology, PCOS, known endometriosis history, prior pelvic surgery, and persistent ovarian cysts. Male partners with clinically apparent varicocele and any accessory gland infection were not included in the study. | None |
| Karimi (2012)(94) | Diabetic men (type 1&2) diagnosed 5 years prior. Thirty-five age-matched normospermic and healthy nondiabetic men were participated voluntarily in this study as control group. | The exclusion criteria included leucocyte concentration >106 per ml of ejaculate, medication or trauma, infection, history of smoking, varicocele, occupational exposure to chemicals or excessive heat and specimens with hyperviscosity. |
| Kaspersen (2013)(95) | None | The only exclusion criterion for the first-time donors was no sperm. Approved donors had been examined and excluded if they had a predisposition to certain inheritable diseases, previous known genital infections, current infection of C. trachomatis or N. gonorrhea (swab), or sero-positivity towards human immunodeficiency virus type1 and 2, human T-cell lymphotropic virus type 1 and 2, hepatitis B and C, and T. pallidum. |
| Kavoussi (2021)(96) | None | Excluded were men who underwent bilateral varicocele repair, with bilateral testicular atrophy, who had testicular atrophy and a varicocele with a history of cryptorchidism, who had undergone varicocele repair for hypogonadism for orchialgia and not for fertility, and who did not obtain a 3-month postoperative semen analysis because they achieved a pregnancy before then or who did not follow up. |
| Kiwitt-Cárdenas (2021)(97) | None | None |
| Krüger (2008)(98) | Pregnant women and their male spouses. | None |
| Kumar (2013)(99) | None | Subjects suffering from chronic diseases, endocrine illnesses and history of fever during previous three months were excluded from both the groups. |
| Kumar (2015)(100) | Fathers of children with NFSHRb: non-familial sporadic heritable retinoblastoma. | Mutations of RB gene in fathers' peripheral blood. Varicocele, vasectomy. Recent fever/infection/drug intake or change in lifestyle in past 1 year. |
| La Vignera (2012)(101) | Oligoasthenoteratozoospermia and grade 3 left varicocele were selected (without other causes of male infertility). The control group comprised healthy, normozoospermic (same exclusion criteria) and presumptively fertile men (fatherhood in the 12 months before the study) without varicocele and with similar age and body mass index (25.2 6 4.4 years old, range 20–30 years; body mass index range: 19.0–24.0 kg/m2). | To exclude subjects with the concomitant presence of an andrological disease known to alter conventional and nonconventional semen parameters. Men (patients and controls) with systemic or endocrine disease; male accessory gland infection; past or present cryptorchidism, microorchidism, cigarette smoking habit, alcohol, or drug abuse; or recent hormonal treatment were excluded. Moreover, patients with less than 50% of viable spermatozoa were excluded from the study. |
| Laqqan (2021)(102) | Males from the same ethnicity and nationality, males have the same food supplementation or a good nutritional status, and non-smokers who have had one child at least. | Diabetes mellitus, alcohol intake, Varicocele, Y chromosome microdeletions, abnormal body mass index, abnormality in hormonal levels, and occupational exposures to known reproductive toxins or excessive heat. |
| Lara-Cerrillo (2020)(103) | Patients presented with clinical unilateral or bilateral grade II or higher varicocele detected by ultrasonography with a venous dilatation greater than 3mm and presence of blood flow reflux, had a diagnosis of infertility and were candidates for MV (microsurgical varicocelectomy). | Exclusion criteria included: (i) evidence of urogenital infections; (ii) absence of varicocele after physical examination and ultrasound; (iii) regular consumers of alcohol, drugs, and tobacco; (iv) recurrent varicocele after surgery; or (v) presenting with late complications after surgery. |
| Le (2020)(104) | The study included men from couples with at least one year of infertility, who were subjected to semen analysis and SDF assay (Halosperm). The selection criteria in this study include men who were diagnosed with infertility according to WHO standards, eligible for the halosperm test and having enough required information regarding anthropometry and biochemical assays. | Patients with acute systemic diseases, acute urinary tract infection, hepatic function disorders, malignant diseases, retrograde ejaculation, or azoospermia were excluded from the study. |
| Le (2021)(105) | The inclusion criteria were as follows: (1) diagnosed with infertility according to the WHO standards as having regular sex without any contraception but still unable to get pregnant after 12 months, (2) having full clinically relevant information, blood biochemistry (including fasting blood glucose and lipidemia test, semen analysis (SA), sperm DNA fragmentation test), and (3) consenting to participate in the study. | The exclusion criteria were as follows: (1) acute urogenital tract infections or being treated for a malignancy affecting spermatogenesis, (2) retrograde ejaculation, inability to ejaculate, and (3) no testis detected through clinical examination and scrotum ultrasound. |
| Lenters (2015)(106) | Partners of pregnant women. | None |
| Li (2012)(107) | All men had grade 2 or 3 varicocele. Those selected as controls had normal seminal parameters, no evidence of endocrine or systemic disease, and no past history of cryptorchidism, varicocele, or orchitis. | Men with azoospermia and evidence of genital tract infection were excluded. |
| Liu (2021)(108) | None | Excluded were those with trauma, family diseases, urological and reproductive diseases (like varicocele, cryptorchidism, prostatitis, epididymitis) sexual dysfunction, medication history (hormones or cytotoxic drugs) and occupational exposure (to zinc, high-density radiation, chemicals, high temperature). |
| Liu (2021)(109) | All patients had no colds and history of special medications in the past 3 months (such as taking antioxidants and drugs affecting the immune systems), excluding patients with abnormal testicular, epididymis, and vas deferens, azoospermia cases were also excluded. | None |
| Long (2007)(110) | Participating men should be over 18 years of age, born in the country where the study was performed and demonstrated fertility by having a pregnant or recently pregnant wife, except for the Swedish study group where fishermen were eligible if the first two criteria were fulfilled. | None |
| Lu (2018)(111) | Partners had not conceived within 12 months after stopping use of contraception. | Stringent exclusion criteria were employed to exclude regular alcohol drinkers, heavy smokers, and men with chronic diseases, urogenital infections, varicocele, azoospermia, and other diseases which might lead to dysspermia, and incomplete data. 32 men with 100% of spermatia or 100% of teratospermia were excluded. |
| Lu (2020)(112) | None | Exclusion criteria for this study included azoospermia and any medical history of tumors, testicular torsion, cryptorchidism, testicular injuries, varicocele, mumps orchitis, and other relevant systemic diseases. |
| Lu (2017)(113) | None | None |
| Ma (2017)(114) | Controls: healthy men were between 24 and 40 years of age. They had fathered children and had shown normal spermiogram values as defined by the World Health Organization. | None |
| Mahfouz (2010)(115) | None | None |
| Mahran (2019)(116) | None | Exclusion criteria included azoospermia, leukocytospermia, recurrent varicocele, previous pelvic surgery and medical treatment for infertility. |
| Malm (2017)(117) | Criteria for being included in the study were as follows: 19–40-year-old men who had lived in respective area for a minimum of 1 year before the study start and were to remain in the area, except a maximum of 5 weeks holiday, during the year of the study. | None |
| Manna (2020)(118) | None | One patient with OAT was not able to provide a second semen sample after 1 h from the first one and, hence, he was excluded from the study. |
| Marchlewska (2016)(119) | None | None |
| Martínez (2021)(120) | Men meeting the following WHO sperm parameters (WHO, 2010): Volume: ≥1.5 mL, Concentration: ≥15 x 106 sperm/mL, Progressive motility: ≥32%,Viability: ≥58%, Morphology: ≥4% | Individuals with azoospermia, cryptozoospermia, retrograde ejaculation, leukocytospermia, or varicocele; subjects submitted to chemotherapy and radiation therapy; patients exposed to pesticides and other toxic agents; and men with a history of infection or fever in the three months prior to treatment were excluded. |
| Mayorgaa-Torres (2015)(121) | None | Exclusion criteria for study participation were any history of urogenital surgery, leukocytospermia (white blood cells > 1 × 106 cells/mL semen), and azoospermia. In addition, self-reported illnesses or use of medication in the three months immediately preceding the study. |
| Mayorgaa-Torres (2016)(122) | None | Exclusion criteria for study participation were any history of urogenital surgery, leukocytospermia (white blood cells >1×106 cells/ml semen), and azoospermia. In addition, self-reported illnesses, or use of medication in the three months immediately preceding the study were recorded. |
| McDowell (2013)(123) | None | None |
| Meseguer (2008)(124) | None | None |
| Mohammed (2015)(125) | None | None |
| Moskovtsev (2009)(126) | None | None |
| Moskovtsev (2009)(127) | 75 men with at least 1-year history of infertility, a palpable varicocele, oligo, astheno, or teratozoospermia were selected from our andrology clinic. At least 1 scrotal vein had a maximum diameter of at least 3mmand retrograde flow was observed at rest or after Valsalva maneuver. Forty healthy fertile volunteers (control group) were also included in this prospective study. | None |
| Moustafa (2004)(128) | Initial DFI over 30%. | None |
| Nazmara (2021)(129) | Non-azoospermic | None |
| Nguyen (2019)(130) | The study included a randomly selected group of patients (n = 31) attending the male infertility clinic with a history of infertility of at least 1 year's duration. | None |
| Ni (2016)(131) | Consume only the drug heroin for more than last four months. Age & BMI-matched controls. The control group contained men with normal semen analysis (based on 2010 WHO criteria) and the heroin-user group consisted of heroin-user men, as determined by a diagnosis by a psychiatrist and supported by laboratory evidence. The following inclusion criteria included the following: the lack of drug use in the control group and heroin consumption for no less than the last four months without concurrent use of other substances or simultaneous use of heroin and other illicit drugs over the study period in the heroin-user group. Furthermore, in every part of the research, participants were excluded from the study if they were suffering from fertility-related diseases, AIDS, or hepatitis. | None |
| Nijs (2011)(132) | 179 male subjects with more than 1‐year history of infertility and clinical varicocele. The second group (control) included healthy subjects (n = 179), who had initiated a natural pregnancy within the previous 12 months. | Men with cryptorchidism, azoospermia, systemic or endocrine disease, genital infection, alcohol or drug abuse, hypogonadism, cigarette smoking habit and patients under hormonal treatment were excluded from the research. |
| Oliveira (2014)(133) | No other infertility related diseases, such as genital infection, hypogonadism, etc, (iii) no occupational agents and factors deteriorating male fertility, such as pesticides, solvents, heat, radiation, etc (Sheiner et al., 2003; Jensen et al., 2006), (iv) no other factors affecting seminal ROS level such as leukocytospermia (Saleh et al., 2002), age>40 years (Cocuzza et al., 2008), antioxidant therapy (Vitamin C, Vitamin E, Coenzyme Q, etc), and (v) no obvious causes of infertility in the female partner. The control group without varicocele comprised of 25 normozoospermic healthy donors with at least one child. | None |
| Oliveira (2018)(134) | None | None |
| Osadchuk (2014)(135) | None | None |
| Pant (2014)(136) | None | None |
| Pearce (2019)(137) | Live in Novosibirsk, clinically healthy for the time of the investigation, not under medical treatment, abstain from sexual activities and alcohol consumption 2-3 days prior to the initiation of the investigation.  The control group included 22 men without genitourinary diseases in their medical history or fertility issues, with normal semen parameters (concentration, motility, morphology) according to the WHO. | Exclusion: azoospermia, cryptorchidism, hypospadiasis, adnexal and testicular cysts. |
| Pelliccione (2011)(138) | None | The exclusion criteria for the subject included a past medical history of testicular dysfunction/urogenital abnormality/mumps, tuberculosis, thyroid dysfunction, or surgical operation, using drugs known to affect gonadal function, or a history of pesticide exposure. |
| Petersen (2018)(139) | Inclusion criteria was being in an infertile relationship. | Documented inflammatory or infectious disease, primary hypogonadism (Klinefelter’s Syndrome, cryptorchidism or testicular injury), the consumption of immunosuppressive medication (e.g., nonsteroidal anti-inflammatory drugs (NSAID), corticosteroids or fish oil), supplements that may alter intestinal function (e.g., probiotics, antibiotics in the last 1 month) or any male hormonal therapy (i.e., aromatase inhibitors, clomiphene citrate, human chorionic gonadotropin (hCG) or testosterone). |
| Pons (2013)(140) | All men attending our clinic (URH-García del Real) for infertility counselling, with one or more of the following characteristics, were offered a DNA fragmentation test in their semen sample (obtained after 3 to 7 days of abstinence): men older than 44, smoking more than 10 cigarettes per day, with a body mass index (BMI) above 25, diabetes mellitus, varicocele, previous chemotherapy treatment, severe oligozoospermia (<10 M/ml in two semen samples), prostatitis, cryptorchidism, and/or a partner with recurrent miscarriage (2 or more miscarriages), implantation failure (2 cycles with embryo transfer or a total 4 embryos transferred in different IVF cycles with no pregnancy), poor embryo morphology and/or fertilization failure.  DFI>=30% | None |
| Rago (2013)(141) | Sixty-three healthy and fertile men (all studied subjects induced pregnancy in the last year), with normal weight [body mass index (BMI) range 19.0-24.5 kg/m2], aged between 18 and 35 years, and non-smoking were carefully selected for enrollment in this study. | Men with systemic (21) and endocrine diseases (22), male accessory gland infection (23), past or present cryptorchidism (24) or varicocele (25), microrchidism (26), cigarette smoking (27), alcohol (28) and/or drug abuse, and recent hormonal treatment were excluded. Overweight or obese subjects and smokers were excluded. |
| Ranganathan (2019)(142) | Male infertile partners with fertile female partners (aged 21‐39 years) were only targeted for the study. In the current study, subjects who have had a history of smoking for at least 8 to 11 years (before enrolment in the study) were categorized based on the smoking index (SI). SI = number of cigarettes smoked/day × number of smoking years. Also, subjects who have habits like drug abuse and regular junk food eaters were all included in the modernized lifestyle factors for negative control. Also, idiopathic reasoned infertile subjects were also selected for the negative control group. | Subjects with a history of genital examination (testis and scrotum), family inheritance, medication allergy, toxins, radiotherapy, and chemotherapy were excluded from the study. In addition to this, the subject with aged (>40), sexually transmitted disease, and occupational inheritance (stress, thermal allergy) were also excluded in this study. Hormonal imbalance and sedentary lifestyle subjects were avoided. Autoimmune disorders like hepatitis B and type I diabetic patients affected subjects were all excluded for this study. |
| Ribeiro (2008)(143) | None | Patients from either group with leukocytospermia were excluded from the study to avoid a false-positive increase in DNA fragmentation due to excessive reactive oxygen species production (from activated leukocytes), not testicular cancer. |
| Romerius (2010)(144) | The inclusion criteria were as follows: (a) male gender, (b) diagnosed before 18 years of age, (c) still alive in September 2004, (d) no active oncological treatment for the last 4 years, and (e) between 18 and 45 years of age at the start of the study in 2004. Ten men were excluded, one as he previously had undergone vasectomy and nine because their disease was considered nonmalignant (e.g., carcinoid in the appendix or spinal hemangioma). Controls: not selected according to fertility (general population) | Azoospermia |
| Rosiak-Gill (2019)(145) | None | For all participants, the exclusion criteria included the following: azoospermia; ethics a testicular torsion, cryptorchidism, testicular injury or cancer; varicocele; co-existing systemic disease; and a history of mumps. |
| Rubes (2010)(146) | Policemen | None |
| Rubes (2021)(147) | Healthy nonsmoking men exposed to traffic air pollution. | Policemen with chronic or andrological diseases and long-term treatment were excluded from the study. |
| Safarinejad (2008)(148) | Patients were included in the study if they were diagnosed with a primary major depression disorder, were younger than 50 years and had been receiving SSRIs 6 months or greater for depression. | Participants in each group with a history of epididymo-orchitis, venereal disease, alcohol consumption, tobacco or illicit drug use, any toxin exposure or concomitant medical problems known to be associated with decreased fertility, such as varicocele, were excluded from study. |
| Safarinejad (2010)(149) | Infertile participants were included in the study after fulfilling the following criteria: absence of constitutional karyotypic abnormalities or Y chromosome deletions, absence of factors in their history with a possible influence for male factor infertility as suggested by WHO Task Force on the Diagnosis and Treatment of Infertility, no female factor, total testicular volume (measured by ultrasonography) ≥ 12 ml, and unwilling childlessness of at least 24 months with a female partner. | Participants in each group with a history of epididymo-orchitis, relevant infectious and genital diseases, concomitant medical problems or use of any medication known to be associated with decreased fertility, severe general diseases such as hepatobiliary disease, and significant renal insufficiency, endocrinopathies such as diabetes mellitus, and thyroid disease, alcohol consumption, tobacco or illicit drug use, or any toxin exposure, were excluded from the study. Other exclusion criteria were as follow: a history of cancer chemotherapy, testosterone, anti-androgens, or antioxidants; and occupational and environmental exposures to potential reproductive toxins. |
| Said (2009)(150) | None | None |
| Saleh (2003)(151) | None | None |
| Savasi (2018)(152) | No clinical signs of HIV. For both groups, smoking and alcohol abuse was stopped at least 2 years before the inclusion in the study and body mass index was ⩽ 25 kg/m2. Only patients with a number of spermatozoa > 5.000.000/ml were included in the study. | Patients with HBV infection or HCV infection coinfections and genital tract infections wre excluded. |
| Sepaniak (2006)(153) | Infertile patients undergoing standard sperm analysis, prior to ART (intra-uterine inseminations (IIU), in vitro fertilization (IVF) with or without intracytoplasmic sperm injection (ICSI)). | None |
| Smit (2010)(154) | None | None |
| Smit (2010)(155) | SCSA was performed in all non-azoospermic postoperative semen samples of men who underwent vasectomy reversal at our tertiary referral outpatient clinic. | None |
| Smith (2007)(156) | None | Retracted testes, leukocytospermia, varicocele, exposure to heat, toxic substances, alcoholism, chronic smoking. |
| Smith (2006)(157) | All patients exhibited grade II or grade III clinical varicocele (Hargreave and Liakatas, 1991) that was diagnosed by palpation as well as by Doppler ultrasound examination. The control group included 25 normozoospermic healthy donors, with unknown fertility, who had a normal genital examination and normal standard semen parameters according to World Health Organization (WHO) criteria (WHO, 2001). | All subjects with evidence of urogenital infections, leukocytospermia, hypogonadism (testicular volume <15 ml), a history of smoking and excessive alcohol and drug consumption were excluded from the study. |
| Spanò (2005)(158) | In all regions, except Sweden, consecutive pregnant women were approached at the entry point for the study and their male spouses were enrolled in the male study. In all countries it was required that both the man and his partner should be >18 years of age and that they should both be born in the country of the study. | None |
| Specht (2012)(159) | Pregnant women and their male partners were encouraged to participate. It was required that both partners were 18 years or more of age and born in the country of the study. | None |
| Ståhl (2009)(160) | All patients who had semen cryopreserved prior to treatment for any malignant disease at the Fertility Centre in the Malmo¨ University Hospital were potentially eligible for the study. Furthermore, we included patients already participating in a longitudinal study on testicular cancer and reproductive function, the TGCC study. Only those having 12 or more straws stored were asked to participate. | None |
| Ståhl (2004)(161) | All men with TGCC who were aged <50 years and who were treated <5 years before inclusion were eligible for the study. | Excluded due to various reasons (bilateral disease, psychologic and psychiatric reasons, hepatitis C virus infection, or linguistic problems). Excluded from further analysis due to azoospermia (n=11), retrograde ejaculation(n=11), or too low sperm counts to allow the analysis of sperm integrity (n=2). |
| Ståhl (2006)(162) | All men with TGCC, below the age of 50, diagnosed <5 years before inclusion, were eligible for the study. | azoospermia (n = 14), retrograde ejaculation (n = 11) and development of contralateral disease after inclusion (n = 2) |
| Stronati (2006)(163) | Over 18, born in given country (Greenland or Poland or Ukraine). | None |
| Taha (2012)(164) | Inclusion criteria were fertile men fathering a child within the prior 2 years with normozoospermic semen analysis. Nonsmokers were all chosen as never having smoked before. | Varicocele, leukocytospermia, undescended testis, testicular atrophy, chronic systemic diseases, autoimmune disorders, occupational exposure to DNA toxins, and antioxidant and/or vitamin intake. |
| Taha (2014)(165) | None | leukocytospermia, undescended testis, testicular atrophy, chronic systemic diseases, autoimmune disorders, female factor infertility and occupational exposure to DNA toxins. |
| Taha (2019)(166) | CHB men (HBs Ag seropositive fertile men >6 months | Patients on anti-HBV treatment or presenting with acute phase of HBV infection were excluded. Also, patients who were smokers, with co-morbid chronic illness (such as diabetes mellitus, liver cirrhosis, systemic hypertension, chronic chest disease, or on chronic use of medications, etc.), with chronic liver disease (such as co-infection with HCV, abnormally raised liver enzymes), or with varicocele, leukocytospermia were excluded. |
| Taha (2016)(167) | fertile men (men who fathered a child within the previous 12 months). | Exclusion criteria were smoking, varicocele, leukocytospermia, chronic medical disorders and men taking antioxidants. |
| Tahamtan (2019)(168) | semen samples and blood were collected from 20 fertile individuals referred for family balancing and 18 men with varicocele (II & III grade) | Infertile men with varicocele that (1) had other infertility related diseases, such as genital infection, hypogonadism, Klinefelter’s syndrome, testicular size discrepancy, anatomical disorders, abnormal hormonal profile, grade I varicocele, recurrent varicocele, azoospermia, previous history of scrotal trauma or surgery, and/or (2) were exposed to occupational agents and factors degrading male fertility, such as pesticides, solvents, heat, radiation, excessive alcohol, drug consumption, and/or (3) had other factors affecting seminal ROS level such as leukocytospermia, age >40 years, were excluded from this study. |
| Talebi (2008)(169) | Patients who had no child after a period of unprotected intercourse for more than 1 year with one or various abnormal semen parameters according to the recommendations of the World Health Organization (1999) were considered as infertile. Varicocele dg made, by the same urologist, for the patients in standing position and via scrotal palpation in a temperature-controlled room (23 C). | None |
| Tanaka (2020)(170) | Over a period of 2 years (2017–2019), 138 infertile men with varicocele (Grade 1, 24 cases; Grade 2, 47 cases; Grade 3, 67 cases) | None |
| Tangal (2019)(171) | (1) at least two unsuccessful ICSI attempts performed with ejaculated spermatozoa; (2) women between 18 and 40 years old; (3) male subjects with no obvious abnormalities noted in the medical history, and physical examination, (4) no evidence of subclinical genital infections, leukocytospermia, cryptorchidism, cancer, or varicocele. | (1) azoospermia; (2) women with history of poor response to ovarian stimulation or fulfilling the Bologna criteria for expected poor responders (25); (3) preimplantation genetic screening (PGS), cryopreserved/thawed embryo transfer cycles (4) uterine or tubal pathology; (5) subjects with genetic disorders; (6) male subjects defined as heavy smokers (>20 cigarettes/day). |
| Tartibian (2012)(172) | (1) unmarried men 18 to 28 years of age; (2) in good health, as determined by a normal physical examination and routine laboratory tests within the previous year; (3) no history of chronic disease, including reproductive disorders; (4) no history of use of medications that could alter the hypothalamic–pituitary– gonadal axis, such as anabolic steroids; (5) regular eating patterns and no history of depressive illness; (6) normal physical and sexual development; (7) not working in professions where the activity might influence reproductive capacity; (8) no relevant previous surgery (e.g., vasectomy reversal or varicocele removal); and (9) appropriate history of physical activity for the different groups described above | Patients with varicocele grade I were excluded from the study. |
| Vagnini (2007)(173) | unselected group of couples undergoing infertility investigation and treatment | None |
| van Brakel (2017)(174) | The acquired group (N = 65) consisted of men diagnosed with acquired UDT as a child for which they were annually monitored until puberty by either the Erasmus Medical Center-Sophia Children’s Hospital or Medical Center Alkmaar (van Brakel et al., 2014). Spontaneous descent was awaited until at least Tanner stage P2G2 and followed by orchiopexy in case of non-descent. Acquired UDT was defined as an UDT for which youth health care physicians at least twice had documented a previous scrotal position. Twenty-two healthy proven fertile men served as controls and donated a semen sample for sperm DNA fragmentation prior to vasectomy. | None |
| Vargas-Baquero (2020)(175) | Spinal cord injury | No spinal cord injury |
| Vaughan (2020)(176) | All males were undergoing an infertility evaluation. | None |
| Vellani (2013)(177) | Male first-attempt IVF patients | "Incomplete responses", diagnosis of varicocele, diagnosis of urogenital tract infection, orchitis, or retractile testes; 18 who had undergone urogenital surgery of any kind, including scleroembolization or tying of sperm ducts; and 8 who had a genetic condition (Klinefelter syndrome) or chronic disease (diabetes mellitus) |
| Vinnakota (2019)(178) | None | None |
| Vivas-Acevedo (2014)(179) | Men with varicocele grades II and III. | Exclusion criteria were the presence of urogenital infections; a positive assay for anti-Chlamydia trachomatis antibodies (ImmunoComb II® Monovalent IgA kit; Orgenics, Yavne, Israel); azoospermia or hypogonadism; and systemic diseases such as cancer and endocrine pathology (and their treatments), which would lead to testicular alterations. |
| Vujkovic (2009)(180) | Fertile and subfertile men were eligible for enrollment. At the IVF intake visit, the couples were invited to participate in the study, and only the men were included in the current analysis. European origin. | If semen was cryopreserved or obtained by microsurgical or percutaneous epididymal sperm aspiration. |
| Wang (2018)(181) | None | Volunteers with any abnormal situation in their urogenital system and volunteers with abstinence time less than 2 days or longer than 7 days were excluded. |
| Wang (2012)(182) | Varicocele, age 20-50, asthenozoospermia, negative anti-sperm AB | Retrograde/no ejaculation, anti-epileptic drugs, anti-tumor or other drugs affecting spermatogenesis and sperm motility, congenital malformation, insemination road obstruction, testicular atrophy (including epididymis), chromosomal anomalies, abnormal sex hormone secretion, serious primary disease (CV, liver, kidney, hematopoietic system, mental illness), do not meet inclusion criteria/incomplete data, do not agree to participate. |
| Wijesekara (2020)(183) | couples investigated for infertility | A history of diabetes mellitus, mumps, tuberculosis, high blood pressure, urinary tract infection, sexually transmitted diseases and testicular injury as known causes of infertility, small testes, varicoceles or any other genital abnormalities, previous genitourinary surgery, long-term medication for systemic illnesses, hormonal treatment or vitamin supplementation, cigarette smokers, azoospermics. |
| Winkle (2008)(184) | 320 unselected patients consulting our IVF and Urological Center were included in this study. These patients neither had any infections or antibiotic treatment during the past 3 months nor did they undergo x- ray or chemotherapy during the past 6 months. | "Unselected patients" |
| Wyrobek (2006)(185) | 22–80 years of age, employed or retired from a government research laboratory. | Exclusion criteria were current cigarette smokers (last 6 months), current fertility or reproductive problems, previous semen analysis with zero sperm count, vasectomy, history of prostate cancer or undescended testicle, or cancer chemo- or radiotherapy. Single specimens from each man were stored at⫺80°C before genomic analyses (see Supporting Text). |
| Yang (2016)(186) | All patients were undergoing treatment at our reproductive medical center for first fresh IVF cycles. All patients had normal chromosome karyotypes. None of the men had Y chromosome micro-deletions or any evident causes of spermatogenic impairment. sperm concentration >0.5×10^6 /mL | None |
| Zequiraj (2019)(187) | None | None |
| Zeyad (2018)(188) | Patients should not have taken any antibiotic 1 week before collection of semen sample. Before collection, patients were advised to wash their hands and genital area with soap and water. | None |
| Zhang (2021)(189) | Couples suffering from infertility were examined for fertility evaluation by a consultant at the center. In the present study, unexplained miscarriage refers to at least one pregnancy loss within 20 gestation weeks but without definite cause(s). | excluding the history of unexplained miscarriage of the female, male infertility refers to the inability of a fertile female to become pregnant (primary infertility) or pregnant again (secondary infertility) after ≥ 12 months of regular unprotected intercourse. We excluded male infertile patient whose spouse was identified with female infertile, such as anatomical abnormalities, endocrine diseases, genetic factors, infection, and immune disorders, etc. At the same time, the patients whose sperm concentration is < 2 million/ml were also excluded for the cell number is too low to count and detect with sperm chromatin structure assay (SCSA) |
| Zhu (2021)(190) | Inclusion criteria is the age from 22 to 40 years, the BMI≥18.5 and they had normal sexual life without contraception and had not given birth for at least 1 year. | Exclusion criteria included regular alcohol drinkers, heavy smokers, chronic diseases, azoospermia, and any other diseases which might lead to dysspermia. |

**Supplementary Table 4.** Risk factor and population definitions in each included article

| **Author (year)** | **Risk factor** | **Risk factor definition** | **Fertility status definition** |
| --- | --- | --- | --- |
| Abdelbaki (2017)(1) | varicocele | not defined | Fertile: normozoospermic |
| Abdullah (2019)(2) | testicular atrophy | not defined | not defined |
| Agarwal (2016)(3) | abstinence | not defined | Fertile: normozoospermic. |
| Agbaje (2008)(4) | diabetes type 1 | not defined | Fertile: partner currently pregnant. |
| Alargkof (2019)(5) | varicocele | not defined | not defined |
| Albani (2019)(6) | age | not defined | not defined |
| Alhathal (2016)(7) | varicocele | not defined | not defined |
| Alshahrani (2014)(8) | age | not defined | not defined |
| Ammar (2021)(9) | varicocele | not defined | not defined |
| Amor (2019)(10) | smoking | participant who smokes more than one packet/day for 10 years or 2 packets/day for 5 years was considered to be a heavy-smoker, and the participant who did not smoke was considered to be a non-smoker | not defined |
| Andersen (2016)(11) | body mass index | not defined | not defined |
| Anifandis (2014)(12) | smoking, alcohol | no smokers (n = 98), moderate smokers (0–1 pack-year history, n = 76), heavy smokers (>1 packs-year history, n = 33). (Alcohol intake) was divided into three groups: no alcohol users (n = 112), moderate alcohol users or alcohol users drinking >0 to <7 units/week (n = 58) and heavy alcohol users or alcohol users drinking >7 units/week (n = 25) | not defined |
| Ayad (2018)(13) | abstinence | not defined | Fertile: normozoospermic. |
| Bandel (2015)(14) | body mass index | not defined | not defined |
| Banks (2021)(15) | vitamin D | not defined | not defined |
| Belloc (2009)(16) | age | not defined | not defined |
| Berg (2021)(17) | prostatitis, age | not defined | not defined |
| Bergamo (2016)(18) | pollution | The semen from the “high impact” group showed higher zinc, copper, chromium and reduced iron levels, as well as reduced sperm motility and higher sperm DNA Fragmentation Index (DFI). Redox biomarkers (total antioxidant capacity, TAC, and glutathione, GSH) and the activity of antioxidant enzymes in semen were lower in the “high impact” group. High environmental impact group: resided in 9 (Acerra, Caivano, Afragola, Casalnuovo, Pomigliano,Brusciano, Giugliano, Cardito and Marigliano; belonging to theProvinces of Naples and Caserta) out of 88 municipalities of the so-called “Land of fires”: this area is officially recognized as a high environmental impact area on the basis of the Campania Region Environmental Protection Agency report, that identified the “Land of Fires” as the Campania area with the highest con-centration of illegal disposal sites of toxic waste. Low environmental impact: resided in 7 municipalities (Oliveto Citra, Contursi Terme, San Gre-gorio Magno, Buccino, Ricigliano, Valva and Colliano) of a SouthernCampania area, in Salerno province, known as “Alto-medio Sele”: this area has a low environmental impact and its economy based mainly on low-to-medium scale farming and without known illegal disposal of toxic wastes | not defined |
| Bian (2004)(19) | fenvalerate (pesticide) | not defined | not defined |
| Boeri (2020)(20) | age, body mass index | not defined | not defined |
| Boeri (2019)(21) | smoking, alcohol | Moderate smokers: 0–1 pack‑year history; heavy smokers: >1 pack‑year history.  Moderate drinkers: up to 2 drinks per day; heavy drinkers: >2 drinks per day. | not defined |
| Boeri (2019)(22) | pre-diabetes | Pre-diabetes was defined according to the clinical criteria detailed by the American Diabetes Association (Diabetes Care 2014; 37 (Suppl. 1): S81). | not defined |
| Bojar (2013)(23) | age, smoking | not defined | not defined |
| Borges (2019)(24) | abstinence | not defined | not defined |
| Bosco (2018)(25) | pollution | not defined | not defined |
| Bozhedomov (2021)(26) | varicocele | not defined | Fertile: partner got pregnant in past 3 months. |
| Brackett (2008)(27) | abstinence, spinal cord injury | not defined | not defined |
| Brahem (2011)(28) | age | not defined | not defined |
| Chavarro (2010)(29) | body mass index | not defined | not defined |
| Chigrinets (2019)(30) | smoking, alcohol | not defined | not defined |
| Comar (2017)(31) | abstinence | not defined | not defined |
| Cortés-Gutiérrez (2017)(32) | human papilloma virus | not defined | Fertile: men with normal genitalia and normal standard semen parameters, who had initiated a normal pregnancy within the past 12 months |
| Cui (2016)(33) | smoking | Mild smoking group (256 cases; ≤9 cigarettes/day), moderate smoking group (365 cases; 10-19 cigarettes/day), heavy smoking group (299 cases; ≥20 cigarettes/day). Short-term smoker group (268 cases; ≤5 years), medium‑term smoker group (282 cases; 5-10 years), long-term smoker group (370 cases; ≥10 years). | not defined |
| Dahan (2020)(34) | abstinence | not defined | not defined |
| Darbandi (2019)(35) | reactive oxygen species | group1(n =39): low (ROS < 20 RLU/s/106 sperm), group 2 (n = 38): mild (20 RLU/s/106 sperm≤ROS < 40 RLU/s/106 sperm), group 3 (n = 31): moderate (40 RLU/s/106 sperm ≤ ROS < 60 RLU/s/ 106 sperm), and group 4 (n =43): high (ROS ≥ 60 RLU/s/106 sperm). | not defined |
| Darbandi (2019)(36) | age | not defined | not defined |
| Das (2013)(37) | age | not defined | Fertile: normozoospermic. Fertility clinic: OAT. |
| De Jonge (2004)(38) | abstinence | not defined | not defined |
| De Win (2021)(39) | varicocele | not defined | not defined |
| Dehghan Marvast (2018)(40) | Chlamydia trachomatis | not defined | not defined |
| Depuydt (2021)(41) | human papilloma virus | not defined | not defined |
| Dieamant (2017)(42) | varicocele | not defined | not defined |
| Domes (2012)(43) | bateriospermia, elevated seminal leukocytes | The four most common species accounted for 90% of bacterial isolates: Enterococcus fecalis (E. fecalis) (56%), E. coli (16%), group B Streptococcus (GBS) (13%), and Staphylococcus aureus (S. aureus) (5%; Fig. 2). Four other bacteria accounted for at least 1% of all bacterial isolates: Klebsiella pneumoniae (2.2%), Proteus mirabilis (1.7%), Citrobacter koseri (1.5%), and Morganella morganii (1.3%). | not defined |
| Dupont (2013)(44) | body mass index | Height and weight were recorded on the day of semen collection and the BMI was calculated (kg m-2). | not defined |
| Eini (2021)(45) | bacteriospermia | Among positive cultures, E. faecalis (E. faecalis) was the most frequent with an occurrence of 25% (15/ 60). Other frequently recognized bacterial species were S. agalactiae (S. agalactiae) (16.66%), E. coli (E. coli) (16.66%), S. aureus (S. aureus) (8.33%), Staphylococcus (S. haemolyticus) (11.66%), Proteus spp. (6.66%), (K. pneumoniae) K. pneumoniae (5%) and multi bacterial (10%). | A subfertile couple was defined as a couple who failed to achieve a clinical pregnancy after 1 year of regular and unprotected sexual intercourse. |
| Eisenberg (2014)(46) | body mass index, waist circumference, sports | By vigorous exercise, I mean a leisure time physical activity that made you sweat and your heart beat faster, such as tennis, running, bicycling,  aerobics, basketball, swimming, or brisk walking. | not defined |
| Elbardisi (2021)(47) | age | not defined | not defined |
| Elbardisi (2020)(48) | oxidation-reduction potential | not defined | not defined |
| Elbardisi (2018)(49) | geography, age | not defined | not defined |
| Elshal (2009)(50) | smoking | not defined | not defined |
| Esfaahani (2010)(51) | varicocele | not defined | not defined |
| Esteves (2015)(52) | varicocele, testicular cc, Chlamydia | not defined | Infertile: min 2 unsuccessful ART. |
| Evenson (2020)(53) | age | not defined | not defined |
| Falahieh (2021)(54) | COVID | not defined | Fertile: partner gave birth within 2 years. |
| Fernandez-Encinas (2020)(55) | varicocele | Asthenoteratozoospermic infertile patients without varicocele (ATZ), including samples from six ATZ patients, with low DDS, high SDF by alkaline and neutral Comet assay. Asthenoteratozoospermic infertile patients with varicocele (ATZ-VAR), including samples from six ATZ patients with varicocele, with high values of DDS and SDF for both Comet assays. | Infertility has been defined by the WHO as the inability to achieve a clinical pregnancy in 12 months of unprotected relationships. Fertile donors: presented normal semen analysis, low DNA degraded sperm (DDS), low SDF by alkaline and neutral Comet assay. |
| Finelli (2021)(56) | varicocele | Varicocele was classified as ‘low’ (subclinical, I-II grades, n = 39) and ‘high’ (III grade, n = 45) grade, according to the severity of the vascular disease (Dubin & Amelar, 1970). Controls included normozoospermic patients with a normal hormone profile. | Fertile: normozoospermic. |
| Frainais (2010)(57) | human immunodeficiency virus | not defined | not defined |
| Gallegos (2008)(58) | Chlamydia trachomatis + Mycoplasma urealyticum | not defined | Fertile: sperm donors. |
| Gao (2020)(59) | Location of semen collection | not defined | not defined |
| Gao (2021)(60) | age | not defined | not defined |
| García-Ferreyra (2015)(61) | age | not defined | not defined |
| García-Peiró (2011)(62) | varicocele | not defined | Fertile: donors of proven fertility. |
| García-Peiró (2014)(63) | varicocele | not defined | not defined |
| Gautam (2015)(64) | smoking, alcohol | Nicotine consumers: smokers and tobacco consumers. Alcohol users, included the subjects who consumed alcohol over 10 years before giving the sample. | not defined |
| Ghandehari-Alavijeh (2019)(65) | varicocele | not defined | Fertile: partner currently pregnant. |
| Ghazavi-Khorasgani (2017)(66) | varicocele | not defined | Fertile: partner currently pregnant. |
| Gill (2019)(67) | low activity at work | ≥ 50% of their time at work (≥ 17.5 h per week) in a sedentary position | not defined |
| Gill (2020)(68) | age | not defined | not defined |
| Gill (2021)(69) | varicocele | not defined | Fertile group consisted of men who had naturally produced offspring in the last 3 years or whose partners were pregnant during the recruitment of participants. Infertile men were selected from couples who were diagnosed/treated for infertility. These subjects had not initiated a pregnancy within the past 12 months of regular, unprotected sexual intercourse. |
| Giwercman (2007)(70) | geography | not defined |  |
| Gosálvez (2011)(71) | abstinence | not defined | Fertile: normozoospermic. Fertile (second line): donors. |
| Grosen (2021)(72) | Methotrexate, inflammatory bowel disease/rheumatic arthritis/psoriatic arthritis | Patients who initiated methotrexate therapy delivered a sample before initiation and a follow-up sample after at least 4 months of clinical remission and maintenance therapy to ensure drug exposure during 1 spermatogenic cycle. | not defined |
| Grosen (2019)(73) | inflammatory bowel disease | For those who stopped anti-TNF-α therapy, samples on and off treatment were collected while the patients were in clinical remission according to the PGA for at least 3 months. Further, duration of therapy was a minimum of 3 months before sampling, and patients were off anti-TNF-α therapy for at least 3 months before follow-up sampling. Remission meant that patients were without symptoms [i.e., normal stool frequency and no abdominal pain for CD patients; stool frequency less than four times a day and no rectal bleeding or urgency for UC patients] | not defined |
| Grosen (2019)(74) | inflammatory bowel disease, Vendolizumab | not defined | not defined |
| Grosen (2019)(75) | inflammatory bowel disease, Thiopurines | IBD patients, we collected semen and blood samples either before initiation or cessation of AZA/6-MP treatment, for paired analysis of semen samples while off and on thiopurines. | not defined |
| Guo (2020)(76) | age | not defined | not defined |
| Håkonsen (2012)(77) | body mass index, smoking, abstinence | Non-smoker: 0 cigarettes | not defined |
| Hammadeh (2010)(78) | smoking | Smokers=heavy smokers 20/day | not defined |
| Hammiche (2011)(79) | geography | not defined | not defined |
| Hansen (2012)(80) | alcohol in last 5 days | not defined | Male factor subfertility was defined as a sperm concentration of ,20 6 106 cells/mL, failure to conceive after 1 year of regular unprotected intercourse with the same partner, duration of child wish of more than 1 year with the same partner, and no prior conception. Fertile men were defined by a sperm concentration of $206106 cells/mL and a prior conception with the current or previous partner. Ethnic background was classified according to the definitions of Statistics Netherlands (2007). |
| Henkel (2003)(81) | reactive oxygen species | not defined | not defined |
| Homa (2019)(82) | seminal oxidative stress | not defined | not defined |
| Horta (2011)(83) | age | not defined | not defined |
| Huang (2011)(84) | di(2-ethylhexyl) phthalate (DEHP) exposure | Workers were categorized into low- and high-DEHP-exposed groups in accordance with the median levels (23.7 mg/m3) of DEHP in ambient air for the 45 participants | not defined |
| Humaidan (2021)(85) | seminal oxidative stress | not defined (MiOXSYS measurement) | Infertile: failed IVF/ICSI.  General population: healthy |
| Iommiello (2015)(86) | ejaculate oxidative stress | Low OS samples (LOS) including levels N1 and N2 and high OS samples (HOS) including levels N3 and N4. LOS samples have optimal/low levels of ROS considered not able to damage cells. HOS samples have levels of ROS so high that may cause pathological effects on sperms such as DNA fragmentation. | Male factor infertility is defined as the inability of a couple to conceive a child after one year of unprotected sexual intercourse with a female that has a normal reproductive history, normal ovulation, and tubal patency. |
| Janghorban-Laricheh (2016)(87) | varicocele | not defined | Fertile: partner currently pregnant. |
| Jeng (2015)(88) | Polycylic aromatic hydrocarbons | not defined | not defined |
| Jeremias (2021)(89) | varicocele | Varicocele was evaluated by scrotal palpation in a temperature-controlled room with adequate illumination, and graded according to Dubin and Amelar (28): (i) Varicocele grade I - dilation of spermatic cord palpable only with Valsalva maneuver; (ii) Varicocele grade II - dilation of spermatic cord easily palpable, with the patient standing, demonstrating marked venous dilation during Valsalva maneuver; (iii) Varicocele grade III - massive dilation of spermatic cord easily visualized with patient standing and intensified ectasia during Valsalva maneuver. | not defined |
| Ji (2011)(90) | 3-phenoxybenzoic acid (3-PBA) | not defined | not defined |
| Ji (2013)(91) | Polycylic aromatic hydrocarbons | not defined | not defined |
| Jurewicz (2018)(92) | diet | food frequency questionnaire (FFQ) | not defined |
| Kabukçu (2021)(93) | abstinence | not defined | not defined |
| Karimi (2012)(94) | diabetes | RAGE (receptor for advanced glycation end products) levels were determined using ELISA and western blot analysis in sperm samples. | Fertile: normozoospermic. |
| Kaspersen (2013)(95) | human herpes virus or human papilloma virus | PCR-based hybridization array that identifies all HHVs and 35 of the most common HPVs. | not defined |
| Kavoussi (2021)(96) | testicular atrophy | To be included in the TA group, the ipsilateral testicle to the varicocele had to measure less than 18 ml and have a volume of at least 3 ml less than the contralateral testicle. | not defined |
| Kiwitt-Cárdenas (2021)(97) | bisphenol A | Creatinine-corrected urinary BPA | not defined |
| Krüger (2008)(98) | geography | not defined | Fertile: partners of pregnant women. |
| Kumar (2013)(99) | ionizing radiation | not defined |  |
| Kumar (2015)(100) | smoking | not defined | Fertile: have child/ren. |
| La Vignera (2012)(101) | varicocele | not defined | Fertile: normozoospermic. |
| Laqqan (2021)(102) | smoking | Heavy smokers’ group (n = 98), those who smoke ≥ 25 cigarettes/day, smoke duration at least 10 years and they still smoking, and (II) non-smokers group, who have never smoked cigarettes in any form during his life (n = 90). | not defined |
| Lara-Cerrillo (2020)(103) | varicocele | not defined | Fertile: sperm donors. |
| Le (2020)(104) | body mass index, metabolic syndrome | We measured the height and weight of all patients. Body mass index (BMI) was calculated as weight (kilograms) divided by the square of height (in meters). According to the Asian classifications for BMI, patients were categorized as obese (>=25 kg/m2), overweight (23.0-24.9 kg/m2), normal (18.5-22.9 kg/m2), and underweight (<18.5 kg/m2). Metabolic syndrome (MetS) was defined based on the NHLBI/AHA-ATP III guidelines. | not defined |
| Le (2021)(105) | metabolic syndrome | not defined | not defined |
| Lenters (2015)(106) | geography | not defined | not defined |
| Li (2012)(107) | varicocele | not defined | Fertile: normozoospermic |
| Liu (2021)(108) | Chlamydia trachomatis + Ureaplasma urealyticum | not defined | Failing to fertilize the wife after more than 12 months’ cohabitation and normal sex without any contraceptive intervention. |
| Liu (2021)(109) | oxidative stress | 8-hydroxydeoxyguanosine (8-OHdG) is the most suitable biomarker for detecting oxidative damage of DNA caused by ROS. For low 8-OHdG the IQR is 1.1-3.8, for high 11.5-25.9. | not defined |
| Long (2007)(110) | geography | not defined | not defined |
| Lu (2018)(111) | body mass index, waist circumference, waist-to-hip-ratio, waist-to-height | not defined | not defined |
| Lu (2020)(112) | age | not defined | not defined |
| Lu (2017)(113) | diabetes | DM diagnosis was confirmed by examining the patients’ detailed medical history and by the results of fasting blood glucose examination. | not defined |
| Ma (2017)(114) | Ureaplasma urealyticum | In U.urealyticum infection group, the U.urealyticum cultures were positive in twice. In control group, the U.urealyticum cultures were negative in twice. | Infertility was defined by a period of at least 24 months of unprotected sexual intercourse without pregnancy. |
| Mahfouz (2010)(115) | seminal reactive oxygen species | Chemiluminescence assay. ROS units: relative light units/sec/106 sperm | not defined |
| Mahran (2019)(116) | varicocele | not defined | not defined |
| Malm (2017)(117) | geography, season (melatonin) | not defined | not defined |
| Manna (2020)(118) | abstinence | not defined | Fertile: normozoospermic. |
| Marchlewska (2016)(119) | testicular cc | not defined | not defined |
| Martínez (2021)(120) | age | not defined | not defined |
| Mayorgaa-Torres (2015)(121) | abstinence | not defined | not defined |
| Mayorgaa-Torres (2016)(122) | abstinence | not defined | not defined |
| McDowell (2013)(123) | tumors | not defined | Fertile: sperm donors. |
| Meseguer (2008)(124) | tumors | not defined | not defined |
| Mohammed (2015)(125) | varicocele | Grade 1 varicocele was diagnosed when reflux was measured at less than 1second, grade II was diagnosed when reflux lasted 1-2 seconds, and grade III was diagnosed when reflux was noted at more than 2 seconds. SDF was measured 3 months after varicocelectomy. | not defined |
| Moskovtsev (2009)(126) | varicocele, bacteriospermia | not defined | not defined |
| Moskovtsev (2009)(127) | age | not defined | not defined |
| Moustafa (2004)(128) | ROS | A cut-off value of 1 3 106 counted photons per minute (c.p.m.) per 20 3 106 sperm/ml was used to differentiate between ROS-negative and -positive patients. (ROS: chemiluminescence assay.) | Healthy controls: Controls consisted of samples obtained from 19 donors of unproven fertility. |
| Nazmara (2021)(129) | heroin | not defined | not defined |
| Nguyen (2019)(130) | varicocele | not defined | not defined |
| Ni (2016)(131) | varicocele | Clinical varicocele severity was graded according to the criteria of Dubin and Amelar (Dubin & Amelar, 1970), while subclinical varicocele was demonstrable by Doppler ultrasound examination without palpable or visible at rest or during Valsalva maneuver. | Definition of infertility, an infertility history of longer than 12 months despite regular unprotected intercourse. |
| Nijs (2011)(132) | age | not defined | not defined |
| Oliveira (2014)(133) | age | not defined | not defined |
| Oliveira (2018)(134) | body mass index | As the number of men with BMI <18.5 kg/m2 (underweight, n = 1) and with BMI≥40 kg/m2 (very severely obese, n = 35) was very low, they were included in the healthy weight and obese groups, respectively. | not defined |
| Osadchuk (2014)(135) | varicocele, prostatitis | 2 men were simultaneously diagnosed with prostatitis and varicocele. One of them was included in the varicocele group because of the mild clinical symptoms of congestive prostatitis, the other was included in the prostatitis group due to the combined manifestation of prostatitis and inflammation of accessory glands. | not defined |
| Pant (2014)(136) | lindane, p-p′-DDE | not defined | Trying to conceive (TTC) group includes those whose female partners failed to achieve pregnancy after >1 year of regular unprotected intercourse and had no diagnosed fertility disorder. Proven fertility group, includes those whose partners had conceived spontaneously within 1 year. |
| Pearce (2019)(137) | body mass index | not defined | not defined |
| Pelliccione (2011)(138) | body mass index, waist circumference, % body fat | not defined | not defined |
| Petersen (2018)(139) | age | not defined | not defined |
| Pons (2013)(140) | abstinence | “One abstinence day protocol”. This protocol required producing up to three semen samples with 1 day of abstinence and measuring sperm DNA fragmentation. | not defined |
| Rago (2013)(141) | cell phone use | not defined | Fertile: pregnancy within previous year |
| Ranganathan (2019)(142) | smoking | Smoker: minimum 1 cigarette/day for over 10 years | not defined |
| Ribeiro (2008)(143) | tumors | Patients with both tumors were classified as non-seminomas due to its more aggressive nature. | Fertile: fathered a child in the 2 years preceding the study. |
| Romerius (2010)(144) | childhood cancer and therapies | Included tumors: brain tumor, lymphoma, leukemia, Wilms, testicular cancer, other | not defined |
| Rosiak-Gill (2019)(145) | age | not defined | Fertile: normozoospermic. Fertility clinic: abnormal semen parameters. |
| Rubes (2010)(146) | smoking, pollution | c-PAHs were detected using high performance liquid chromatography (HPLC) with fluorimetric detection & personal monitoring of traffic-related VOC (benzene, toluene, ethylbenzene, m-, p- and o-xylene). Urinary cotinine to assess smoking. Benzo[a]pyrene (ng/m3) - winter: 1.03 ± 0.77, spring: 0.16 ± 0.05, cPAH (ng/m3) - winter: 6.03 ± 4.34, spring: 3.78 ± 0.65, Benzene (g/m3) - winter: 6.00 ± 2.69, spring: 5.39 ± 7.60. (All above values are totals for both smokers and non-smokers combined). | not defined |
| Rubes (2021)(147) | age | not defined | not defined |
| Safarinejad (2008)(148) | selective serotonin reuptake inhibitor | not defined | Fertile: normozoospermic. |
| Safarinejad (2010)(149) | mustard gas | The estimation of the individual exposure to mustard gas was qualitatively coded (mild, moderate, and severe) based on the history of the intensity of exposure, the injuries recorded on the  medical records and the remaining complications from previous mustard gas exposure. | Fertile cases had fathered a child within the last 5 years and had above reference values on all 3 semen parameters according to WHO criteria. |
| Said (2009)(150) | tumors | not defined | Fertile: not defined (proven fertility). |
| Saleh (2003)(151) | varicocele | not defined | Infertile: min 1 year. A group of healthy fertile volunteers (n=16) who had initiated a natural pregnancy within the past 12 months and had a normal genital examination was included as a control group. |
| Savasi (2018)(152) | highly active antiretroviral therapy, age | not defined | not defined |
| Sepaniak (2006)(153) | smoking | The smoking intoxication was assessed by questionnaire and measured with the CO-Tester®. Men who had never smoked as well as men who had stopped  smoking more than 6 months prior to the test were considered non-smokers. Every man who had smoked cigarettes for more than 6 months and was still smoking during the test was considered a smoker. Smokers were categorized in three groups: mild (from 1 to 9 cigarettes per day), moderate (from 10 to 19 cigarettes per day) and heavy (up to 20 cigarettes per day). | not defined |
| Smit (2010)(154) | tumors and treatment | Tumor therapy: BEP+/-radiotherapy | not defined |
| Smit (2010)(155) | vasectomy reversal | Median time from vasectomy to vasectomy reversal was 7.0+/-5.0 years. (median + sd). Semen analysis was done a median of 5.5+/-3.7 months postoperatively (range 1.9 to 20.5). | not defined |
| Smith (2007)(156) | orchidopexy | not defined | not defined |
| Smith (2006)(157) | varicocele | Clinical varicocele: gr 2 or 3. | Fertile: normozoospermic. |
| Spanò (2005)(158) | pollution | p,p'-DDE were extracted from serum by solid phase extraction using on-column degradation of the lipids and analysis by gas chromatography mass spectrometry | Fertile: partners of pregnant women |
| Specht (2012)(159) | pollution | Analysis for PFHxS, PFOS, PFOA, PFNA, PFDA, perfluoroundecanoic acid (PFUnDA) and perfluorododecanoic acid (PFDoDA) was performed using liquid chromatography tandem mass spectrometry (LC–MS/MS). | Fertile: partner currently pregnant |
| Ståhl (2009)(160) | tumors and treatment | not defined | not defined |
| Ståhl (2004)(161) | tumor treatment | not defined | not defined |
| Ståhl (2006)(162) | tumor treatment | not defined | not defined |
| Stronati (2006)(163) | pollution | The levels of CB-153 and p,p0-DDE were determined  by the Department of Occupational and Environmental Medicine of Lund University Hospital applying solidphase extraction using on-column degradation of the lipids and analysis by gas chromatography mass spectrometry | Fertile: partners of pregnant women |
| Taha (2012)(164) | smoking | not defined | Fertile: child within 2 years |
| Taha (2014)(165) | smoking, varicocele | not defined | not defined |
| Taha (2019)(166) | hepatitis B virus | HBs Ag seropositive men. | Fertile: fathered a child within a year. |
| Taha (2016)(167) | body mass index | Obese men (BMI >30 kg/m2). | Fertile: fathered a child within a year. |
| Tahamtan (2019)(168) | varicocele | not defined | not defined |
| Talebi (2008)(169) | varicocele | not defined | Fertile: sperm donors. Infertile: no pregnancy achieved within 1 year. |
| Tanaka (2020)(170) | varicocele | not defined | Fertile: normozoospermic. |
| Tangal (2019)(171) | human papilloma virus | Human papilloma virus genotyping was performed using an IVD validated ready to use commercial kit (PapilloCheck, Greiner Bio-One, GmbH, Germany) according to manufacturer’s instructions. Microarray hybridization methodology is used by the procedure and 24 types of HPV (6, 11, 16, 18, 31, 33, 35, 39, 40, 42, 43, 44/55, 45, 51, 52, 53, 56, 58, 59, 66, 68, 70, 73, and 82) were detected. | not defined |
| Tartibian (2012)(172) | sports | Recreationally active men= 2 to 3 days per week for a minimum of 4 to 5 hours per week and a maximum oxygen consumption of 47 to  53 mL/min−1/kg−1 | not defined |
| Vagnini (2007)(173) | age | not defined | not defined |
| van Brakel (2017)(174) | undescended testes | Acquired UDT was defined as an UDT for which youth health care physicians at least twice had documented a previous scrotal position. | Fertile men: men before vasectomy. |
| Vargas-Baquero (2020)(175) | spinal cord injury | not defined | Fertile: normozoospermic. |
| Vaughan (2020)(176) | age | not defined | not defined |
| Vellani (2013)(177) | anxiety from in vitro fertilization | First-attempt IVF patients (anxiety from IVF). | not defined |
| Vinnakota (2019)(178) | age | not defined | Fertile: sperm donors  Fertility clinic: presents for infertility treatment |
| Vivas-Acevedo (2014)(179) | varicocele | Varicocele was classified as grade II (palpable without Valsalva maneuver) or grade III (visible through the scrotal skin). | Fertile: normozoospermic. |
| Vujkovic (2009)(180) | diet | FFQ. 'Traditional Dutch’ diet HIGH = characterized by high intakes of meat, potatoes and whole grains and low intakes of beverages and sweets. The ‘Health Conscious’ dietary pattern shows high intakes of fruits, vegetables, fish and whole grains. | not defined |
| Wang (2018)(181) | sleep | Munich Chronotype Questionnaire (MCTQ) for the measurement of sleep duration | not defined |
| Wang (2012)(182) | varicocele | Varicocele grade 1: only with Valsalva, grade 2: reflux when taking a deep breath, grade 3: even when breathes calmly. | Fertile controls: within half a year of pregnancy. |
| Wijesekara (2020)(183) | Lead exposure | The minimum detection limit for Pb was 0.32 μg/L.= Pb positive patients. Seminal plasma Pb was estimated by atomic absorption spectrophotometry after digestion with nitric acid. | not defined |
| Winkle (2008)(184) | age | not defined | Fertile: normozoospermic, no ART history |
| Wyrobek (2006)(185) | age | not defined | not defined |
| Yang (2016)(186) | body mass index | overweight BMI group: >28 kg/m2 | not defined |
| Zequiraj (2019)(187) | age | not defined | not defined |
| Zeyad (2018)(188) | bacteriospermia | Nine species of bacteria belonging to five genera, Staphylococcus, Escherichia, Streptococcus, Enterococcus and Klebsiella, were identified, and it was clear that Staphylococcus was the highest incidence rate matching 15% | not defined |
| Zhang (2021)(189) | age | not defined | not defined |
| Zhu (2021)(190) | body mass index | overweight: BMI≥28 | not defined |

**Supplementary Table 5.** Risk of bias assessment using the QUIPS tool

| **Author (year)** | **Study participation** | **Study attrition** | **Prognostic factor measurement** | **Outcome measurement** | **Study confounding** | **Statistical analysis and reporting** |
| --- | --- | --- | --- | --- | --- | --- |
| Abdelbaki (2017)(1) | Low | Moderate | Low | Low | Low | Low |
| Abdullah (2019)(2) | Low | NA | Low | Low | Moderate | Low |
| Agarwal (2016)(3) | Moderate | Low | Low | Low | Moderate | Moderate |
| Agbaje (2008)(4) | Moderate | NA | Low | Low | Moderate | Low |
| Alargkof (2019)(5) | Moderate | NA | Low | Low | Moderate | Low |
| Albani (2019)(6) | Moderate | NA | Low | Low | High | Moderate |
| Alhathal (2016)(7) | Low | Low | Low | Low | Moderate | Low |
| Alshahrani (2014)(8) | Low | NA | Low | Low | Low | Low |
| Ammar (2021)(9) | Low | Low | Low | Low | Moderate | Low |
| Amor (2019)(10) | Low | NA | Low | Low | Moderate | Moderate |
| Andersen (2016)(11) | Low | NA | Low | Low | Low | Moderate |
| Anifandis (2014)(12) | Low | Low | Low | Low | Low | Moderate |
| Ayad (2018)(13) | Moderate | NA | Low | Low | Moderate | Moderate |
| Bandel (2015)(14) | Low | NA | Low | Low | Low | Low |
| Banks (2021)(15) | Low | Moderate | Low | Low | Low | Moderate |
| Belloc (2009)(16) | Low | NA | Low | Low | High | Low |
| Berg (2021)(17) | Moderate | Low | Low | Low | Low | Moderate |
| Bergamo (2016)(18) | Moderate | NA | Low | Low | Low | Low |
| Bian (2004)(19) | Low | NA | Low | Low | Low | Moderate |
| Boeri (2020)(20) | Low | NA | Low | Low | Low | Moderate |
| Boeri (2019)(21) | Low | NA | Low | Low | Low | Low |
| Boeri (2019)(22) | Low | NA | Low | Low | Low | Moderate |
| Bojar (2013)(23) | Low | NA | Moderate | Low | Moderate | Low |
| Borges (2019)(24) | Low | High | Low | Low | Moderate | Low |
| Bosco (2018)(25) | Low | NA | Low | Low | Moderate | Low |
| Bozhedomov (2021)(26) | Low | High | Moderate | Low | Moderate | Low |
| Brackett (2008)(27) | Low | NA | Low | Low | Low | Moderate |
| Brahem (2011)(28) | Moderate | Low | Low | Low | Moderate | Low |
| Chavarro (2010)(29) | Low | NA | Low | Moderate | Low | Moderate |
| Chigrinets (2019)(30) | Moderate | NA | Moderate | Low | Low | Low |
| Comar (2017)(31) | Low | NA | Low | Low | Low | Low |
| Cortés-Gutiérrez (2017)(32) | Low | NA | Low | Low | Moderate | Low |
| Cui (2016)(33) | Low | NA | Low | Moderate | High | Low |
| Dahan (2020)(34) | Low | Low | Low | Low | Moderate | Low |
| Darbandi (2019)(35) | Low | Low | Low | Low | Moderate | Moderate |
| Darbandi (2019)(36) | Moderate | NA | Low | Low | High | Low |
| Das (2013)(37) | Low | NA | Low | Low | High | Low |
| De Jonge (2004)(38) | Low | Moderate | Low | Low | High | Low |
| De Win (2021)(39) | Low | Low | Low | Low | Low | Moderate |
| Dehghan Marvast (2018)(40) | Low | NA | Low | Low | Moderate | Low |
| Depuydt (2021)(41) | Low | Low | Low | Low | Moderate | Moderate |
| Dieamant (2017)(42) | Low | NA | Moderate | Low | Low | Low |
| Domes (2012)(43) | Low | NA | Low | High | High | Moderate |
| Dupont (2013)(44) | Low | NA | Low | Low | High | Moderate |
| Eini (2021)(45) | Low | NA | Low | Low | Moderate | Moderate |
| Eisenberg (2014)(46) | Low | Moderate | Low | Low | Low | Moderate |
| Elbardisi (2021)(47) | Low | NA | Low | Low | Low | Moderate |
| Elbardisi (2020)(48) | Low | NA | Low | Low | Moderate | Moderate |
| Elbardisi (2018)(49) | Low | NA | Low | Low | Moderate | Low |
| Elshal (2009)(50) | Low | NA | Low | Low | High | Low |
| Esfaahani (2010)(51) | Low | NA | High | Low | High | Low |
| Esteves (2015)(52) | Low | NA | Low | Low | High | Moderate |
| Evenson (2020)(53) | Low | NA | Low | Low | High | Moderate |
| Falahieh (2021)(54) | Moderate | Low | Low | Low | High | High |
| Fernandez-Encinas (2020)(55) | Moderate | NA | High | Moderate | High | Low |
| Finelli (2021)(56) | Low | NA | Low | Low | Low | Low |
| Frainais (2010)(57) | Low | NA | Low | Low | Low | Low |
| Gallegos (2008)(58) | Low | Low | Moderate | Low | High | Low |
| Gao (2020)(59) | Low | NA | Low | Low | Low | Moderate |
| Gao (2021)(60) | Low | NA | Low | Low | Low | High |
| García-Ferreyra (2015)(61) | Moderate | NA | Low | Low | High | Low |
| García-Peiró (2011)(62) | Moderate | NA | Low | Low | High | Low |
| García-Peiró (2014)(63) | Low | NA | Low | Low | High | Moderate |
| Gautam (2015)(64) | Moderate | NA | Low | Low | Moderate | Moderate |
| Ghandehari-Alavijeh (2019)(65) | Moderate | NA | Low | Low | High | Moderate |
| Ghazavi-Khorasgani (2017)(66) | Low | NA | High | Low | High | Moderate |
| Gill (2019)(67) | Low | NA | Low | Low | Moderate | Low |
| Gill (2020)(68) | Low | NA | Low | Low | High | Low |
| Gill (2021)(69) | Low | NA | Low | Low | Moderate | Low |
| Giwercman (2007)(70) | Low | NA | Low | Low | High | Low |
| Gosálvez (2011)(71) | Moderate | Low | Low | Low | High | Low |
| Grosen (2021)(72) | Low | Low | Moderate | Low | Low | Moderate |
| Grosen (2019)(73) | Low | Low | Low | Low | Low | Moderate |
| Grosen (2019)(74) | Moderate | NA | Low | Low | Low | Moderate |
| Grosen (2019)(75) | Low | Low | Low | Low | Low | Moderate |
| Guo (2020)(76) | Low | NA | Low | Low | High | Low |
| Håkonsen (2012)(77) | Low | NA | Low | Low | Low | Low |
| Hammadeh (2010)(78) | Low | NA | Moderate | Low | Moderate | Low |
| Hammiche (2011)(79) | Low | Moderate | Low | Low | Low | Moderate |
| Hansen (2012)(80) | Low | NA | Low | Low | Low | Moderate |
| Henkel (2003)(81) | Moderate | NA | Low | Low | Low | Low |
| Homa (2019)(82) | Low | NA | Low | Low | High | Moderate |
| Horta (2011)(83) | Moderate | NA | Low | Low | High | Moderate |
| Huang (2011)(84) | Low | NA | Low | Low | Low | Low |
| Humaidan (2021)(85) | Moderate | Low | Low | Low | Moderate | Low |
| Iommiello (2015)(86) | Low | NA | Low | Low | High | Moderate |
| Janghorban-Laricheh (2016)(87) | Low | NA | Low | Low | High | Moderate |
| Jeng (2015)(88) | Low | NA | Low | Low | Low | Low |
| Jeremias (2021)(89) | Low | NA | Low | Low | Moderate | Low |
| Ji (2011)(90) | Low | NA | Low | Low | Low | Low |
| Ji (2013)(91) | Low | NA | Low | Low | Low | Low |
| Jurewicz (2018)(92) | Low | NA | Low | Low | Low | Low |
| Kabukçu (2021)(93) | Low | Low | Low | Low | Low | Low |
| Karimi (2012)(94) | Low | NA | Moderate | Low | High | Low |
| Kaspersen (2013)(95) | Low | NA | Low | Low | High | Low |
| Kavoussi (2021)(96) | Low | NA | Low | Low | Moderate | Low |
| Kiwitt-Cárdenas (2021)(97) | Low | NA | Low | Low | Low | Low |
| Krüger (2008)(98) | Low | NA | Low | Low | High | Moderate |
| Kumar (2013)(99) | Low | NA | Low | Low | Low | Moderate |
| Kumar (2015)(100) | Low | NA | Low | Low | High | Low |
| La Vignera (2012)(101) | Low | NA | Moderate | Low | Moderate | Moderate |
| Laqqan (2021)(102) | Low | NA | Low | Low | Moderate | Low |
| Lara-Cerrillo (2020)(103) | Moderate | NA | Low | Low | Low | Moderate |
| Le (2020)(104) | Low | NA | Low | Low | Low | Low |
| Le (2021)(105) | Low | NA | Low | Low | Low | Low |
| Lenters (2015)(106) | Low | NA | Low | Low | Low | Moderate |
| Li (2012)(107) | Moderate | NA | Low | Low | High | Low |
| Liu (2021)(108) | Low | NA | Low | Low | High | Low |
| Liu (2021)(109) | Low | NA | Low | Low | Low | Moderate |
| Long (2007)(110) | Low | NA | Low | Low | Moderate | Moderate |
| Lu (2018)(111) | Low | Low | Low | Low | Low | Low |
| Lu (2020)(112) | Low | NA | Low | Low | High | Low |
| Lu (2017)(113) | Low | NA | Low | Moderate | High | Low |
| Ma (2017)(114) | Low | NA | Low | Low | High | Moderate |
| Mahfouz (2010)(115) | Low | Low | Low | Low | Low | Low |
| Mahran (2019)(116) | Low | NA | Low | Moderate | Moderate | Low |
| Malm (2017)(117) | Low | NA | Low | Low | Moderate | Low |
| Manna (2020)(118) | Low | Low | Low | Low | High | Low |
| Marchlewska (2016)(119) | Low | NA | High | Low | Moderate | Low |
| Martínez (2021)(120) | Low | NA | Low | Low | High | Low |
| Mayorgaa-Torres (2015)(121) | High | NA | Low | Low | High | Moderate |
| Mayorgaa-Torres (2016)(122) | High | NA | Low | Low | High | Moderate |
| McDowell (2013)(123) | Low | NA | Moderate | Low | Moderate | Moderate |
| Meseguer (2008)(124) | Low | NA | Moderate | Low | High | Moderate |
| Mohammed (2015)(125) | Low | Low | Low | Low | Moderate | Low |
| Moskovtsev (2009)(126) | Low | NA | Moderate | Moderate | High | Low |
| Moskovtsev (2009)(127) | Low | NA | Low | Low | High | Low |
| Moustafa (2004)(128) | Moderate | NA | Low | Low | High | Moderate |
| Nazmara (2021)(129) | Low | NA | Low | Low | Low | Low |
| Nguyen (2019)(130) | Low | Low | High | Low | High | Low |
| Ni (2016)(131) | Low | NA | Low | Low | Low | Low |
| Nijs (2011)(132) | Low | Low | Low | Low | High | Low |
| Oliveira (2014)(133) | Low | NA | Low | Low | Low | Low |
| Oliveira (2018)(134) | Low | NA | Low | Low | Low | Low |
| Osadchuk (2014)(135) | Low | NA | Moderate | Low | Low | Low |
| Pant (2014)(136) | Low | NA | Low | Low | High | Moderate |
| Pearce (2019)(137) | Moderate | NA | Low | Low | Low | Low |
| Pelliccione (2011)(138) | High | Low | Low | Low | Moderate | Low |
| Petersen (2018)(139) | Low | Low | Low | Low | Low | Low |
| Pons (2013)(140) | High | Low | Low | Low | Low | Low |
| Rago (2013)(141) | Moderate | NA | Low | Low | Low | Moderate |
| Ranganathan (2019)(142) | Low | Low | Low | Moderate | Moderate | Moderate |
| Ribeiro (2008)(143) | Low | Low | Low | Low | Moderate | Low |
| Romerius (2010)(144) | Low | NA | Low | Low | Low | Low |
| Rosiak-Gill (2019)(145) | Low | NA | Low | Low | High | Low |
| Rubes (2010)(146) | Low | NA | Low | Low | High | Low |
| Rubes (2021)(147) | Low | NA | Low | Low | High | Low |
| Safarinejad (2008)(148) | Low | NA | Low | Low | High | Moderate |
| Safarinejad (2010)(149) | Low | NA | Low | Low | Low | Low |
| Said (2009)(150) | Low | NA | Moderate | Low | High | Low |
| Saleh (2003)(151) | Moderate | Low | Low | Low | High | Moderate |
| Savasi (2018)(152) | Low | Low | Low | Low | Low | Low |
| Sepaniak (2006)(153) | Low | Low | Low | Low | High | Moderate |
| Smit (2010)(154) | Low | Low | Low | Low | High | Low |
| Smit (2010)(155) | Low | Low | Low | Low | Moderate | Low |
| Smith (2007)(156) | Low | NA | Low | Low | Moderate | Low |
| Smith (2006)(157) | Low | NA | Low | Low | High | Low |
| Spanò (2005)(158) | Low | NA | Low | Low | Low | Moderate |
| Specht (2012)(159) | Low | NA | Low | Low | Low | Moderate |
| Ståhl (2009)(160) | Low | NA | Low | Low | Moderate | Moderate |
| Ståhl (2004)(161) | Low | Low | Low | Low | High | Moderate |
| Ståhl (2006)(162) | Low | NA | Low | Low | Moderate | Moderate |
| Stronati (2006)(163) | Low | NA | Low | Low | Low | Moderate |
| Taha (2012)(164) | Low | NA | Low | Low | Moderate | Low |
| Taha (2014)(165) | Low | NA | Moderate | Low | High | Low |
| Taha (2019)(166) | Low | NA | Low | Low | Moderate | Low |
| Taha (2016)(167) | Low | NA | Moderate | Low | Moderate | Low |
| Tahamtan (2019)(168) | Moderate | NA | Low | Low | Moderate | Moderate |
| Talebi (2008)(169) | Moderate | Low | Moderate | Moderate | High | Low |
| Tanaka (2020)(170) | Low | Low | High | Low | Low | Low |
| Tangal (2019)(171) | High | Low | Low | Low | Moderate | Moderate |
| Tartibian (2012)(172) | Low | Low | Low | Low | Low | Low |
| Vagnini (2007)(173) | Low | NA | Low | Low | Low | Low |
| van Brakel (2017)(174) | Low | NA | Low | Low | Moderate | Moderate |
| Vargas-Baquero (2020)(175) | Low | NA | Low | Low | Low | Moderate |
| Vaughan (2020)(176) | Low | NA | Low | Low | High | Low |
| Vellani (2013)(177) | Low | NA | Low | Low | Low | Low |
| Vinnakota (2019)(178) | Low | NA | Low | Low | Low | Low |
| Vivas-Acevedo (2014)(179) | Low | Low | Low | Low | Moderate | Low |
| Vujkovic (2009)(180) | Low | High | Low | Low | Low | Moderate |
| Wang (2018)(181) | Low | NA | Low | Low | Low | Moderate |
| Wang (2012)(182) | Low | NA | Low | Low | High | Low |
| Wijesekara (2020)(183) | Moderate | NA | Low | Low | Low | Low |
| Winkle (2008)(184) | Low | NA | Low | Low | High | Low |
| Wyrobek (2006)(185) | Moderate | NA | Low | Low | High | Low |
| Yang (2016)(186) | Moderate | NA | High | Low | Moderate | Low |
| Zequiraj (2019)(187) | Low | NA | Low | Low | High | Low |
| Zeyad (2018)(188) | Moderate | NA | Low | Low | Moderate | Low |
| Zhang (2021)(189) | Low | NA | Low | Low | Moderate | Low |
| Zhu (2021)(190) | Moderate | NA | Moderate | Low | Low | Low |

**NA:** not applicable

**Supplementary Table 6.** Articles also looking at pregnancy or birth as an outcome

| **Author (year)** | **Pregnancy or birth as an outcome in terms of SDF (yes/no)** | **Main findings in terms of pregnancy or birth** |
| --- | --- | --- |
| Amor (2019)(10) | No | Pregnancy rate, which was significantly higher in the group of non-smokers than in that of the heavy-smokers (0.60 ± 0.49% vs. 0.38 ± 0.48%; *P* = 0.013) |
| Banks (2021)(15) | No | There were no significant  differences in pregnancy or live birth rates according to  vitamin D status.  Male 25(OH)D level  <20 ng/mL was associated with a higher rate of pregnancy  loss (5/8 vs. 4/31, P Ľ .009).  All nine pregnancy losses occurred in couples  with a male 25(OH)D level <30 ng/mL (9/25 vs. 0/14,  P Ľ .015). |
| Bojar (2013)(23) | Yes | A statistically significant relationship was confirmed between sperm DNA fragmentation, and the percentage of pregnancies obtained during the first year of (infertility) treatment; patients with a lower DFI more frequently became fathers during the first year of trying, compared to the remainder (t=2.51; P=0.013). |
| Borges (2019)(24) | Yes | Negative correlations were  observed between increasing EA length and fertilization rate (B:  _0.983, CI: _1.954 to _0.011, p = 0.047), blastocyst formation  rate on day 5 (B: _2.384, CI: _4.552 to _0.216, p = 0.031),  implantation rate (B: _3.299, CI: _5.388 to _1.260, p = 0.002)  and pregnancy rate (Exp(B): 0.506, CI: 0.290–0.882, p = 0.016).  EA length was not associated with high-quality embryos rate on  day 3 or miscarriage rate.  In this model, mean EA length in the positive pregnancy group  was 3.14 _ 1.64 days and 4.83 _ 3.66 days in the negative pregnancy  group (p = 0.043).  Regarding ICSI outcomes,  higher rates of fertilization (85.5 _ 2.2% vs. 77.3 _ 2.7%,  p = 0.021), high-quality embryos on day 3 (56.8% vs. 41.6%,  p = 0.022), blastocyst formation on day 5 (50.2 _ 4.7% vs.  35.6 _ 4.9%, p = 0.046), implantation (24.8 _ 4.1% vs. 7.3 _ 4.3%,  p = 0.005) and pregnancy (40.0% vs. 10.0%, p = 0.016) were  observed in EA ≤ 4 days comparedtoEA > 4 days group (Table 6).  Pregnancy rate tended to be higher in  Group 1 (EA  length of one day) compared to the other groups (Group 2, EA length of 2 days; Group 3, EA  length of 3 days; and Group 4, EA length of 4 days) ; however, statistical significance  was not reached, probably due to a small sample size  (Group 1: 69.0% vs. Group 2: 24.0% vs. Group 3: 27.0% vs. Group 4:  35.0%, p = 0.062). |
| Chavarro (2010)(29) | No | There were no apparent differences in hormone levels (LH, FSH) in subgroups defined by history of previous pregnancy or history of previous infertility examination. |
| Depuydt (2021)(41) | Yes | None of the 31 inseminations in which the sperm tested positive for HPV led to pregnancy, (100.0% sensitivity and 17.0% specificity, ROC analysis area under the curve 0.59, (95% CI 0.52 to 0.65 p > 0.05), even when DFI% was below 26% (Figure 1). |
| Elbardisi (2021)(47) | No | When comparing the two groups, normal fertilisation was negatively affected by increased paternal age (P= 0.01). |
| Gallegos (2008)(58) | No | Fertility outcome could be preliminarily determined in a group of 30 patients. Pregnancy was achieved in only 12.5% of couples who tried it during the course of antibiotic (macrolide, a tetracycline, or quinolone) treatment (6 weeks minimum; n=16). Otherwise, it was successful in 85.7% of couples that attempted pregnancy 3–6 months after therapy (n=14; r =0.73, P<.0001; c2 test, P<.0001). |
| García-Ferreyra (2015)(61) | No | There was no difference in the normal fertilization (2PN) between the studied groups (≤39 years: 82.8%; 40–49 years: 75.8%; and ≥50 years: 82.4%). Percentages of zygotes that underwent cleavage (100%, 92.2%, and 95.4%), mean cell number (7.3 ± 1.01, 7.3 ± 0.93, and 6.5 ± 1.22), and good quality embryos on day 3 (83.3%, 85.2%, and 70.4%) were similar from the groups of ≤39 years, 40–49 years, and ≥50 years, respectively. Blastocyst formation rate was significantly lower in the group of men ≥50 years compared to the other two evaluated groups (P<0.05), but the percentages of good quality blastocysts were not associated with advancing paternal age (P = no significant). According to the chromosomal status of embryos, the advanced paternal age was significantly associated with high aneuploidy rates in embryos; thus, 73.9% embryos from the group of ≥50 years were aneuploidies compared to 59.1% in the group of ≤39 years and 61.1% in the group of 40–49 years (P<0.05) |
| Håkonsen (2012)(77) | No | The adjusted back-transformed median DFI was highest in the high exposure groups of maternal age, maternal pre-pregnancy BMI, maternal alcohol consumption, maternal binge drinking, maternal coffee and tea consumption, paternal alcohol intake as well as parental TTP (time to pregnancy) and in the group of women examined or treated for infertility, however, when tested in the regression models, these were not statistically significantly different from the reference groups. |
| Henkel (2003)(81) | Yes | There was no direct correlation between the percentage of TUNEL-positive spermatozoa and fertilization rate (r = 0.0113; P= 0.8718), embryo fragmentation rate (r = 0.0406; P= 0.5855) and pregnancy (r = –0.0889; P= 0.2016) for IVF data (Table 1). After performing ROC analysis (Table 2) and using the calculated cut-off value of 36.5% for the percentage of TUNEL-positive spermatozoa for the distinction of groups, a significant difference between TUNEL-positive (>36.5% TUNEL-positive spermatozoa) and TUNEL-negative (<36.5% TUNEL-positive spermatozoa) ejaculates could be found for pregnancy (P= 0.0218), but not for the fertilization rate (P= 0.4484; Figure 1). While the mean pregnancy rate for TUNEL-negative ejaculates was 34.7%, only 18.7% of the patients fell pregnant if the percentage of TUNEL-positive spermatozoa in the ejaculate was higher than 36.5%. The incidence of TUNEL-positive ejaculates (>36.5% TUNELpositive spermatozoa) amongst the IVF patients was 31.3%.  As observed for IVF, there were also no direct correlations between DNA fragmentation and fertilization rate, embryo fragmentation rate or pregnancy for ICSI. |
| Kabukçu (2021)(93) | Yes | Comparison of total sperm count, total progressive sperm count, progressive sperm motility, inseminated sperm count, and post-gradient sperm DNA fragmentation percentages did not show a significant difference between cycles with and without pregnancy. The percentage of sperm DNA fragmentation percentage was 24.89 ± 12.89 in pregnant couples and 21.71 ± 11.69 in non-pregnant couples (p = 0.288). |
| Li (2012)(107) | No | Of the 19 patients recruited for this study, 7 couples achieved pregnancy after surgical treatment and 3 couples failed to conceive. Of the couples that achieved pregnancy, 5 conceived spontaneously and 2 achieved pregnancy by IVF or ICSI. |
| Martínez (2021)(120) | Yes | Results also showed that the miscarriage rate in the group with males aged ≥40 years and altered DNA fragmentation levels (Group 4) was significantly higher; nevertheless, the ongoing pregnancy rate was not different between groups (Table 3). |
| Meseguer (2008)(124) | Yes | We have performed 30 ICSI cycles from frozen samples, with 15 pregnancies; however, this is obviously a low number of patients to obtain adequate conclusions about the relevance of DNA fragmentation to assisted reproduction success in cancer patients. |
| Mohammed (2015)(125) | Yes | In 15 out of 75 varicocele patients (20%), clinical pregnancy (confirmed by ultrasound detection of fetal pulse) was achieved. Those with positive pregnancy outcome showed significant improvement in sperm count and sperm motility compared with negative pregnancy group (𝑃 < 0.05). Also, those with positive pregnancy outcome had significantly lower DNA fragmentation % by acridine orange, but there is no significant difference in sperm DNA condensation by flow cytometry on comparing them to others who failed to make their partners conceive. |
| Nijs (2011)(132) | No | Logistic regression analysis of male age and pregnancy rate (PR) could not identify any correlation in the overall patient population (45.5% PR, B = )0.202, P = 0.81), or in each male age class (Ł34 years: 51.1% PR, B = )0.24, P = 0.77, 35–39 years: 41% PR, B = 0.004, P = 0.98; ‡40 years: 38.3% PR, B = 0.065, P = 0.45). |
| Smit (2010)(155) | Yes | No significant differences  in DFI, sperm concentration or vitality, male age and  obstructive interval were noted between the 2 groups (vasectomy reversal vs. proven fertile). All patients were dichotomized as having low or high DFI based on a 30% threshold, as frequently described in the literature.12,13 DFI was less and greater than  30% in 11 (42%) and 15 (58%) of 26 patients with spontaneous pregnancy, in 5 (42%) and 7 (58%) of 12 with ART pregnancy, and in 9 (60%) and 6 (40%) of 15  with no pregnancy, respectively. |
| Tangal (2019)(171) | No | The cycle characteristics and outcomes of the couples with infected HPV (Group I) and non-infected male partners (Group II). Values are presented as median (minimum-maximum):  Group I (n=9): Fertilization rate (%):82.5 (25-100), Implantation rate (%): 29, Positive HCG, n (%): 4 (44), Clinical pregnancy rate, n (%): 1 (11), Pregnancy loss, n (%): 3 (33).  Group II (n=108): Fertilization rate (%): 76.5 (33-100), Implantation rate (%): 33, Positive HCG, n (%): 44 (40), Clinical pregnancy rate, n (%): 31 (28), Pregnancy loss, n (%): 11 (10). |
| Yang (2016)(186) | No | When we compared the high-quality embryo (grade I and grade II) rate between the two groups (BMI:20-25, BMI>28), we found that it was significantly lower in the overweight BMI group (Fig. 1b). Furthermore, the clinical pregnancy rate was also significantly lower for this group than for the normal BMI group (Fig. 1c). To confirm the relationship between male BMI and pregnancy rate in IVF, we performed a multivariate logistic regression analysis. After adjusting for female BMI, female age, basal FSH level, male age, number of embryos transferred and sperm count, the data showed that males with a BMI > 28 kg/m2 were associated with lower clinical pregnancy rates. |

**Detailed results – associated health conditions (varicocele, impaired glucose tolerance, tumors, infections):**

**

**

**Supplementary Figure 1.:** Comparison of patients’ sperm DNA fragmentation values with and without varicocele subdivided based on sperm DNA fragmentation assays used (continuous data)


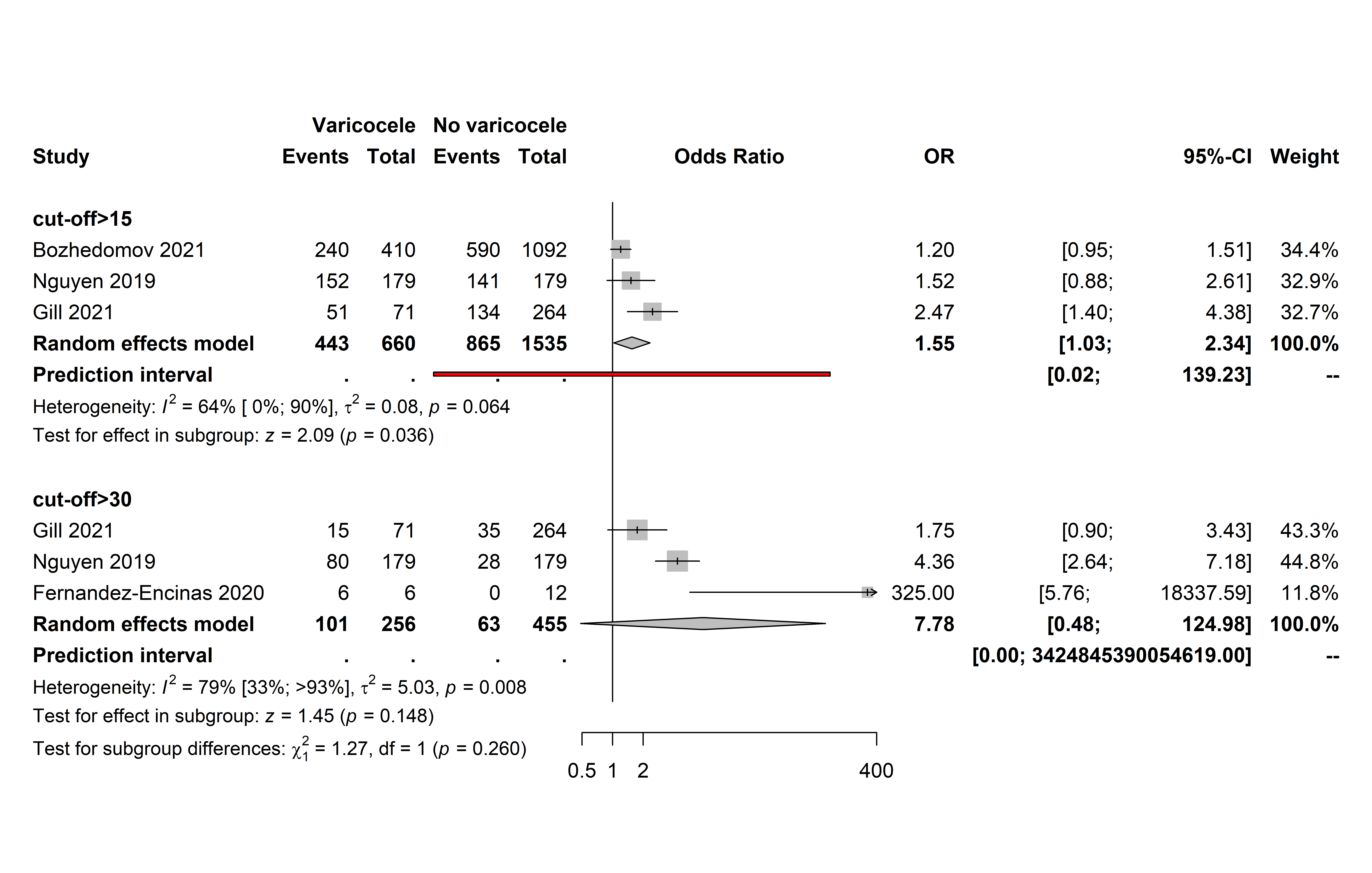
**Supplementary Figure 2.:** Comparison of patients’ sperm DNA fragmentation values with and without varicocele subdivided based on different cut-off values





**Supplementary Figure 3.:** Comparison of patients’ sperm DNA fragmentation values with palpable varicocele (grade 2 or 3) and the lack of palpable varicocele (grade 1 or no varicocele) subdivided based on sperm DNA fragmentation assays used (continuous data)

**

**

**Supplementary Figure 4.:** Comparison of patients’ sperm DNA fragmentation values with and without varicocele subdivided based on the fertility status of patients (continuous data)

**
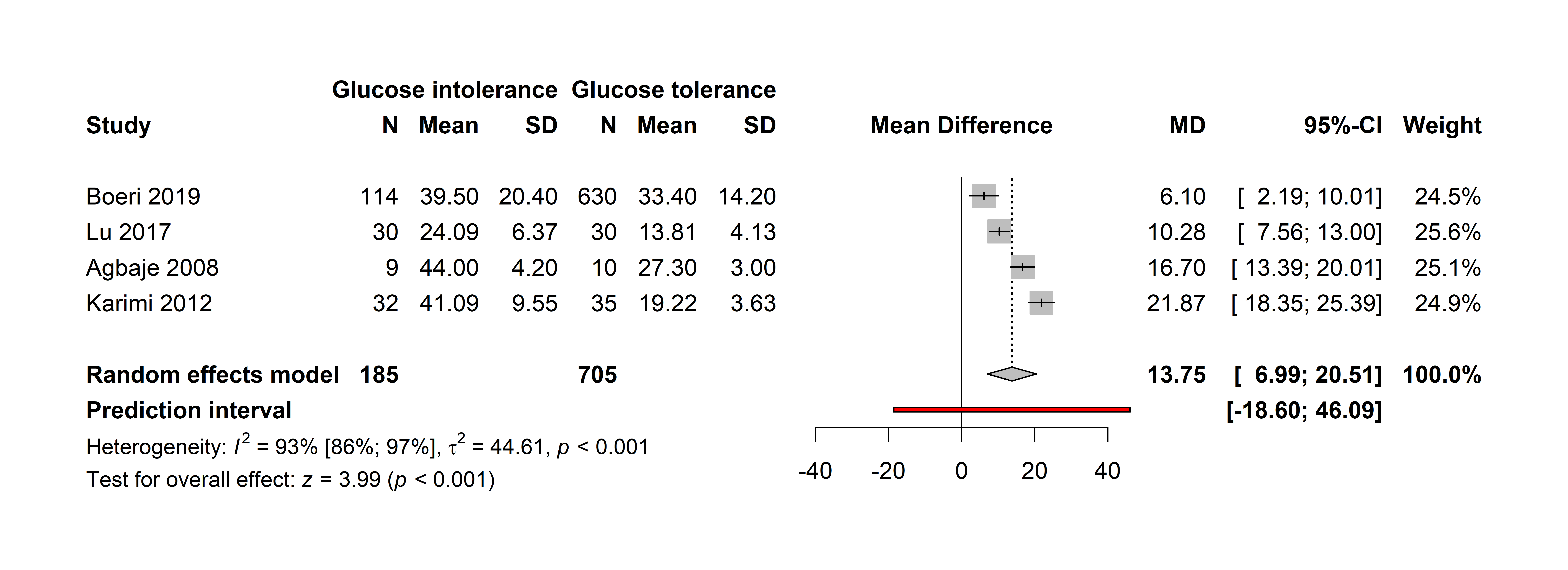
**

**Supplementary Figure 5.:** Comparison of patients’ sperm DNA fragmentation values with impaired and normal glucose tolerance (continuous data)

**
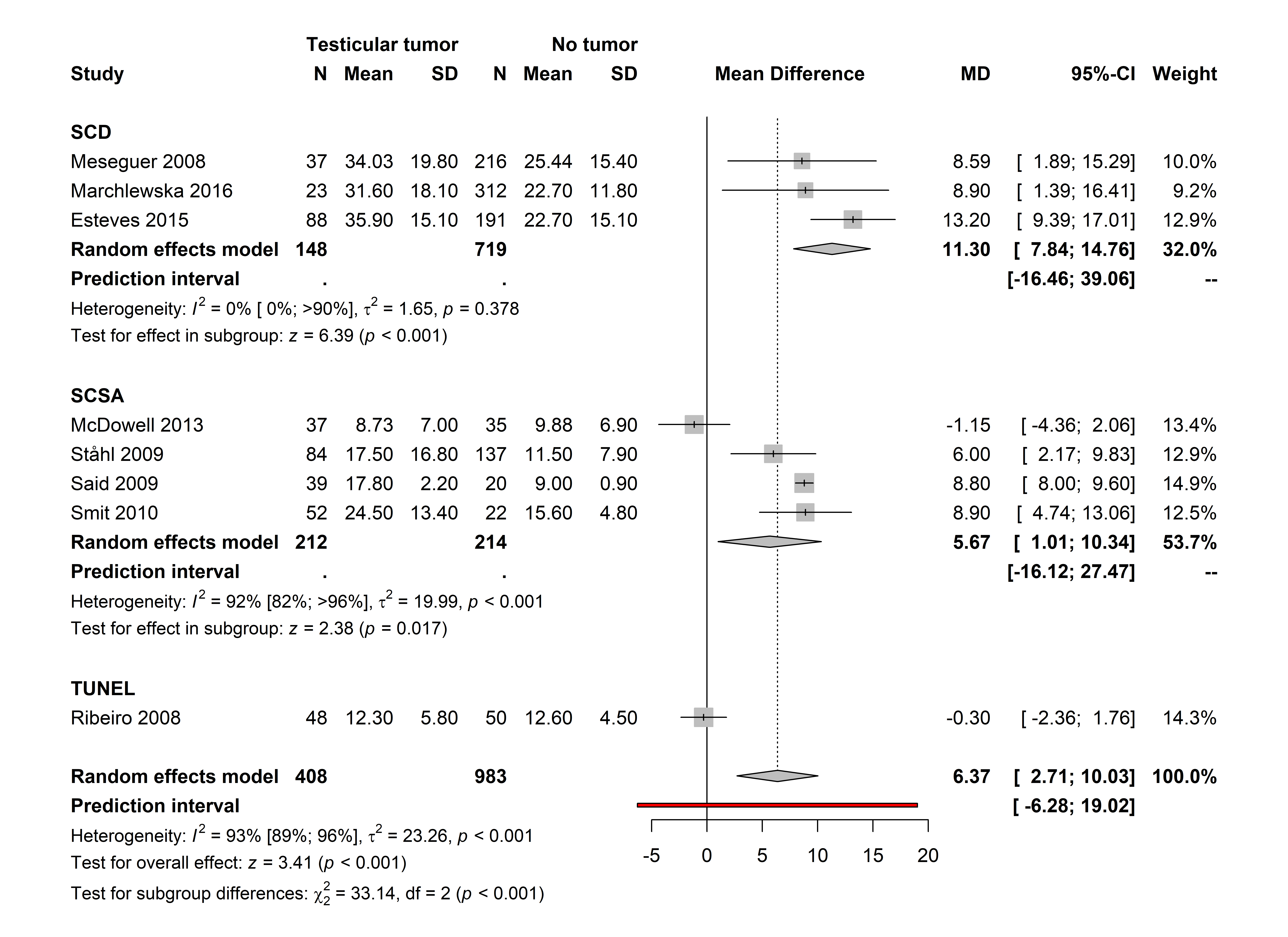
**

**Supplementary Figure 6.:** Comparison of patients’ sperm DNA fragmentation values with and without testicular tumors subdivided based on sperm DNA fragmentation assays used (continuous data)

**
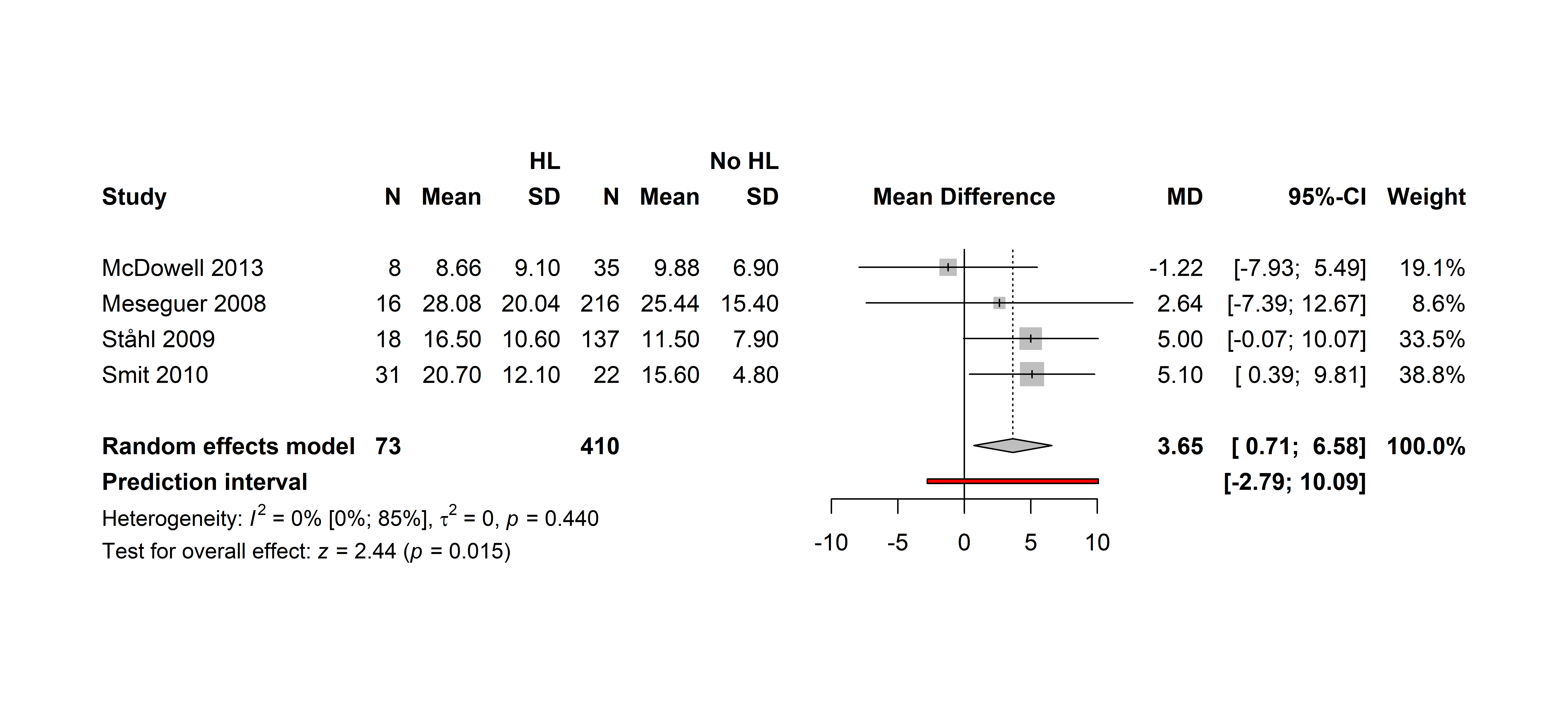
**

**Supplementary Figure 7.:** Comparison of patients’ sperm DNA fragmentation values with and without Hodgkin-lymphoma (HL) (continuous data)

**
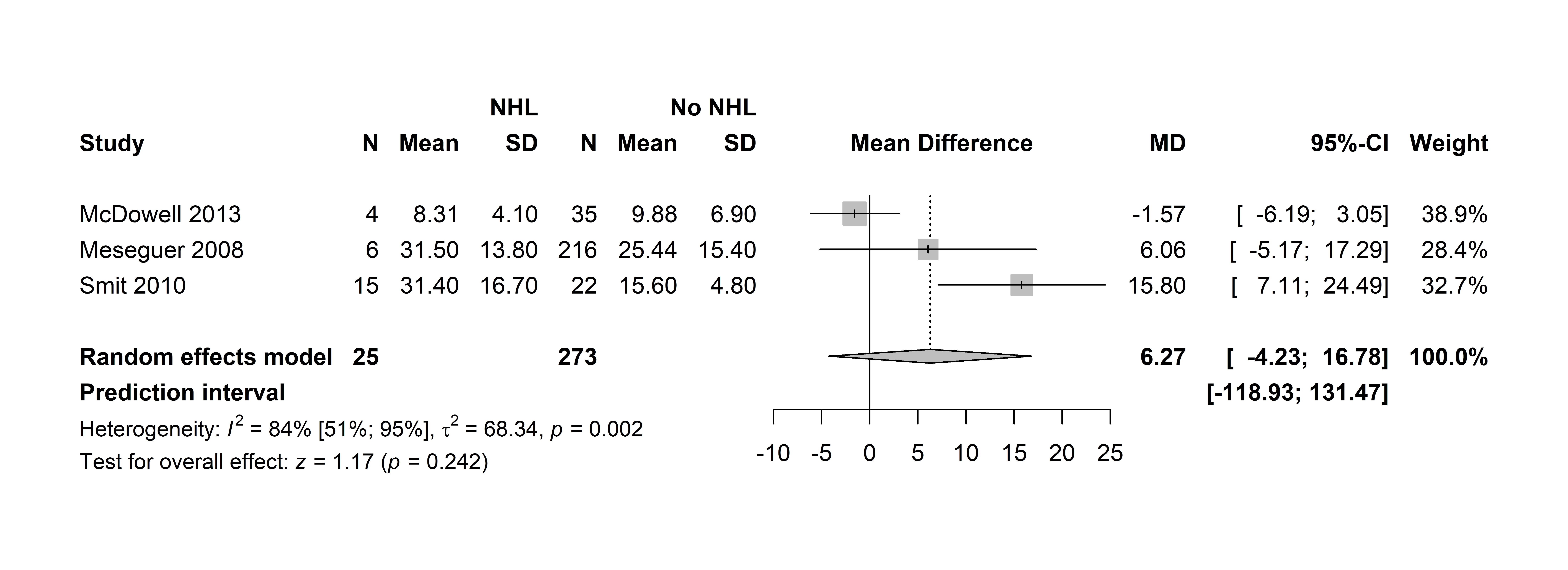
**

**Supplementary Figure 8.:** Comparison of patients’ sperm DNA fragmentation values with and without non-Hodgkin lymphoma (NHL) (continuous data)

**
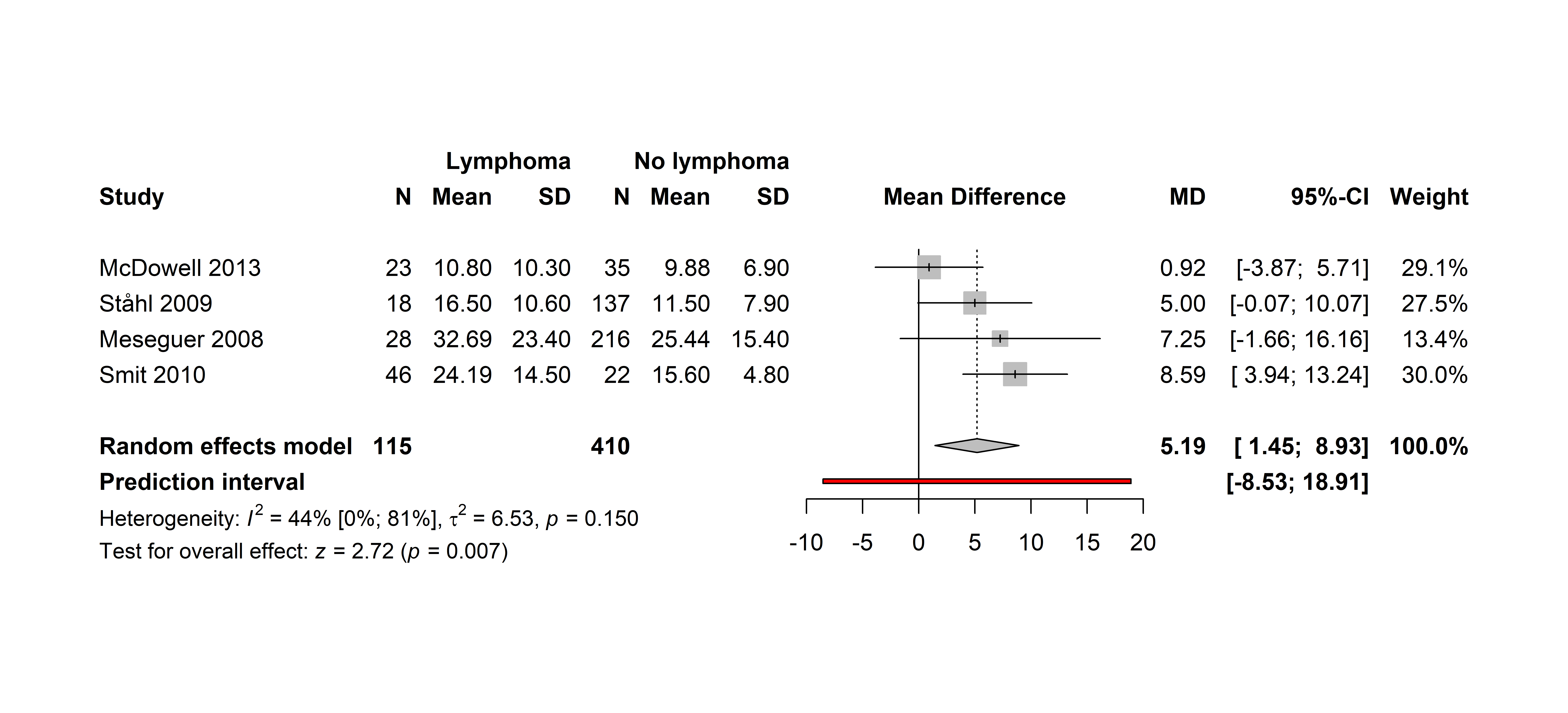
**

**Supplementary Figure 9.:** Comparison of patients’ sperm DNA fragmentation values with and without lymphomas (continuous data)

**
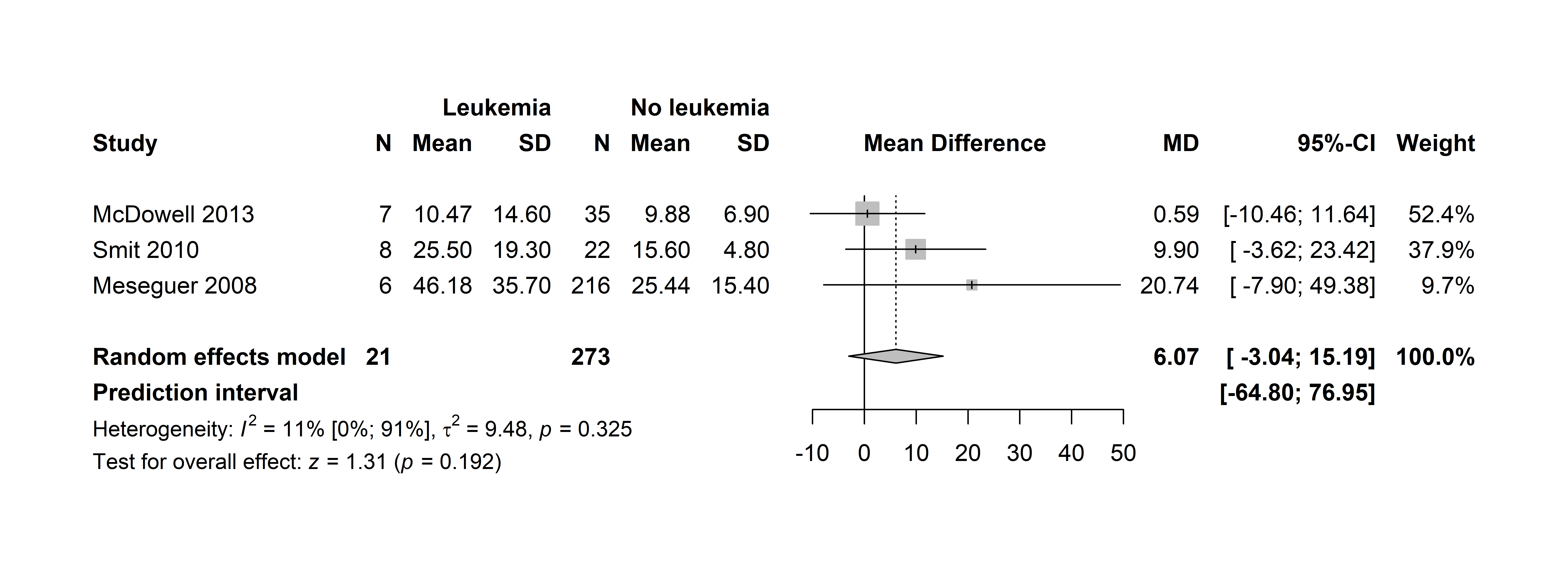
**

**Supplementary Figure 10.:** Comparison of patients’ sperm DNA fragmentation values with and without leukemia (continuous data)

**
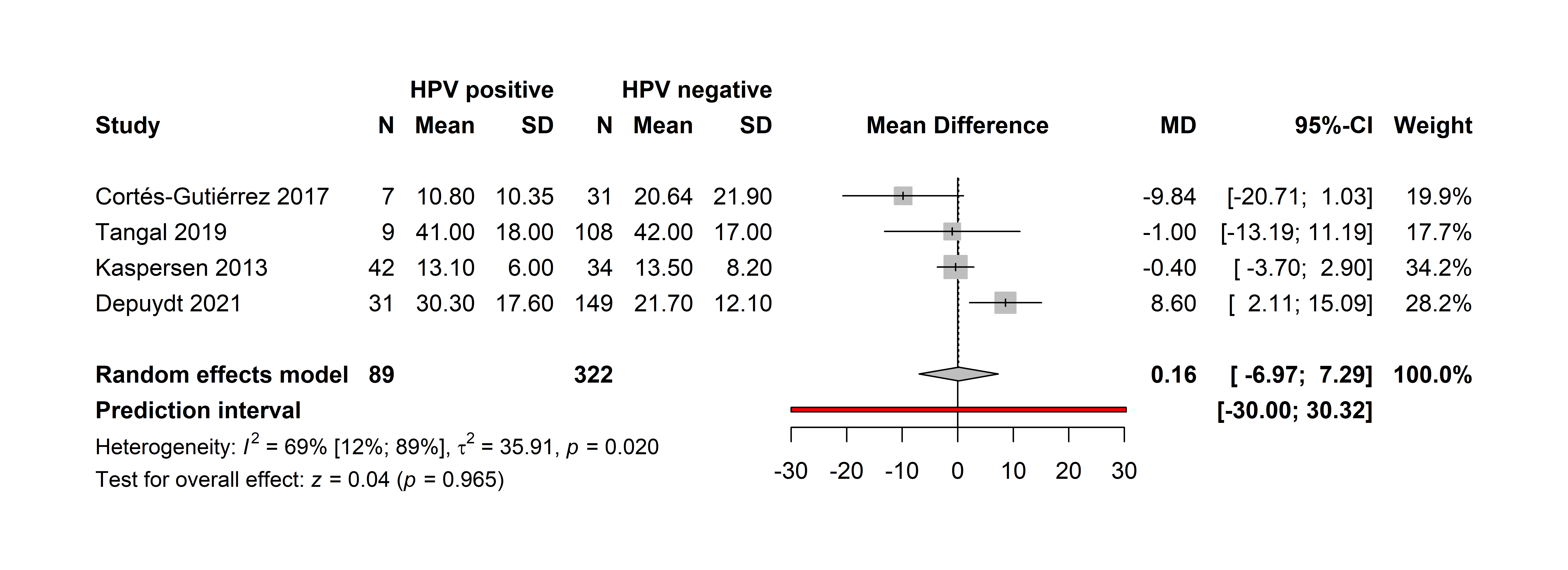
**

**Supplementary Figure 11.:** Comparison of patients’ sperm DNA fragmentation values with and without human papilloma virus (HPV) infections (continuous data)

**
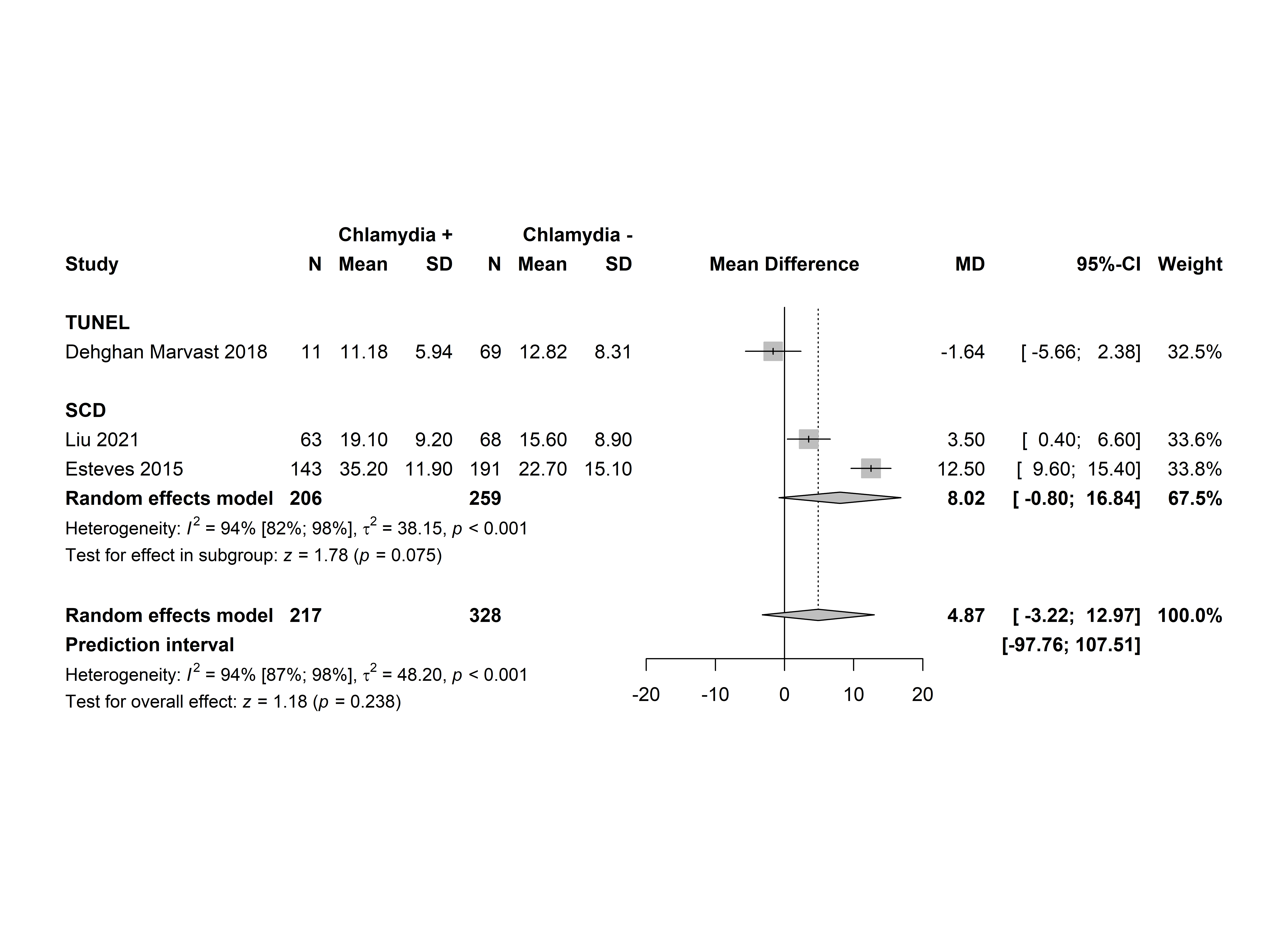
**

**Supplementary Figure 12.:** Comparison of patients’ sperm DNA fragmentation values with and without *Chlamydia* infections (continuous data)


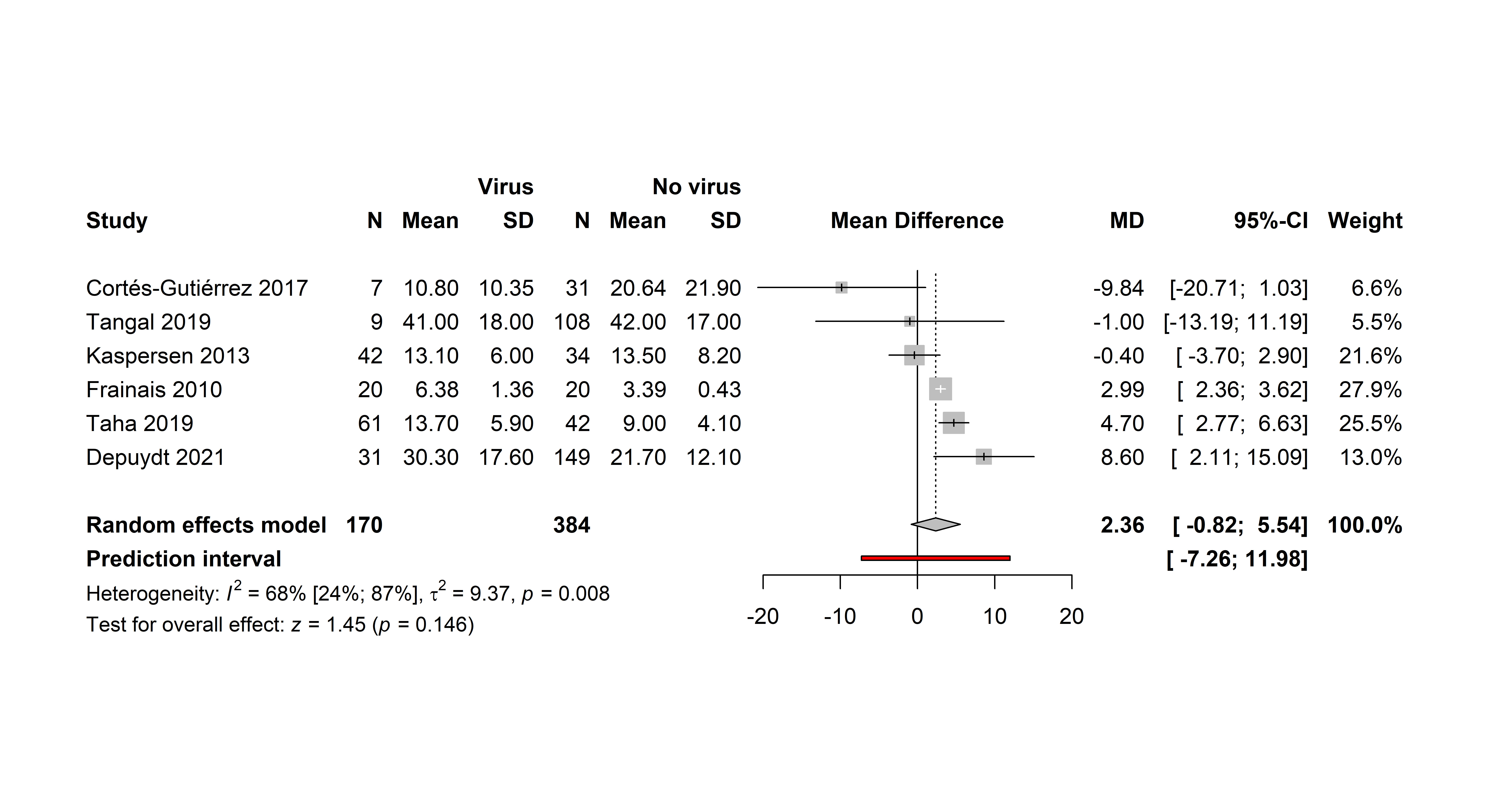


**Supplementary Figure 13.:** Comparison of patients’ sperm DNA fragmentation values with and without viral infections (continuous data)

**

**

**Supplementary Figure 14.:** Comparison of patients’ sperm DNA fragmentation values with and without bacterial infections subdivided based on sperm DNA fragmentation assays used (continuous data)

**

**

**Supplementary Figure 15.:** Comparison of patients’ sperm DNA fragmentation values with and without sexually transmitted infections (STI) subdivided based on sperm DNA fragmentation assays used (continuous data)

**Detailed results – lifestyle factors (smoking, alcohol consumption, body mass index, sexual abstinence):**

**

**

**Supplementary Figure 16.:** Comparison of smokers and non-smokers’ sperm DNA fragmentation values subdivided based on sperm DNA fragmentation assays used (continuous data)

**
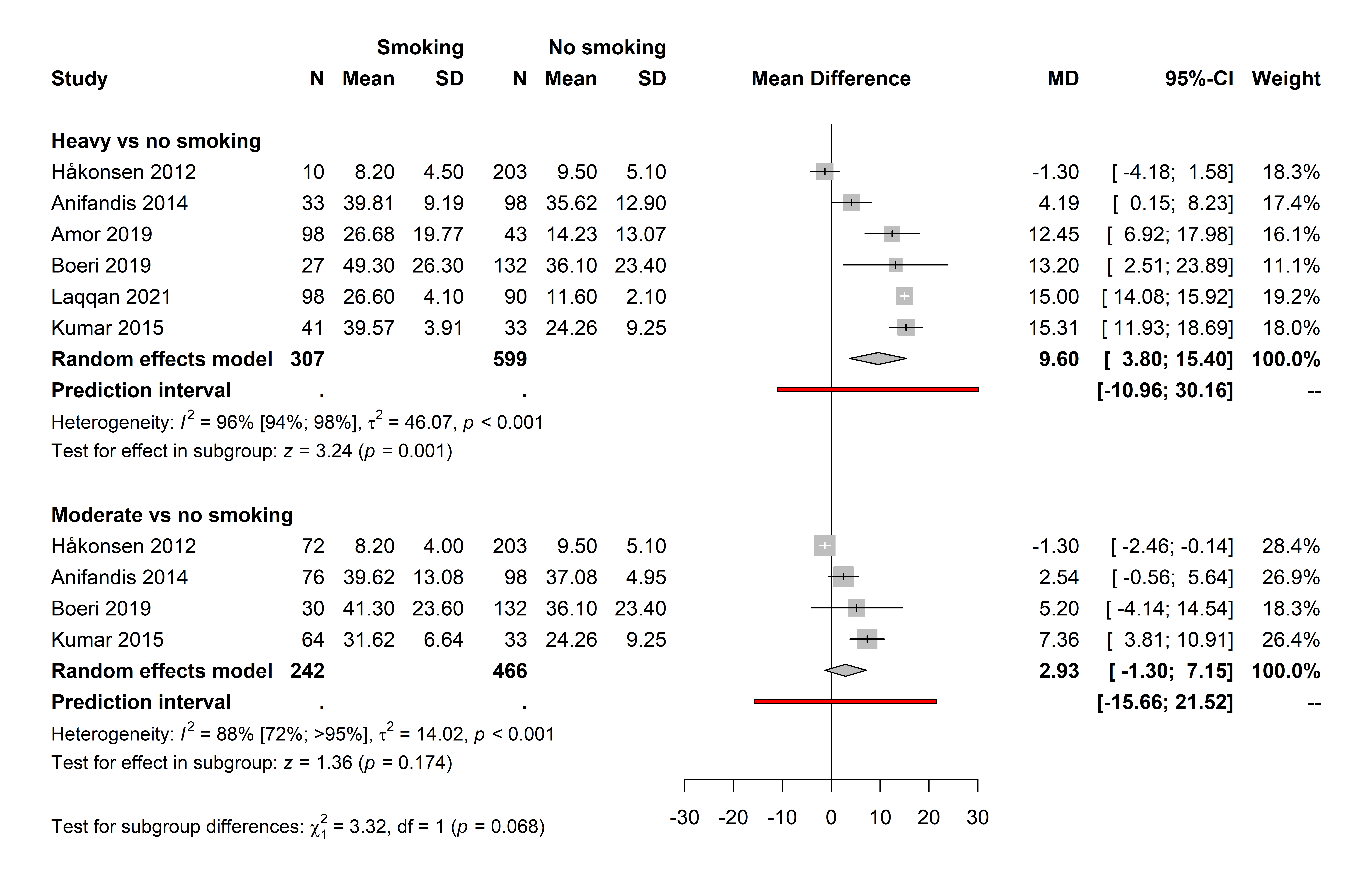
**

**Supplementary Figure 17.:** Comparison of heavy smokers with non-smokers, and moderate smokers with non-smokers’ sperm DNA fragmentation values (continuous data)

**

**

**Supplementary Figure 18.:** Comparison of smokers and non-smokers’ sperm DNA fragmentation values subdivided based on the fertility status of patients (continuous data)


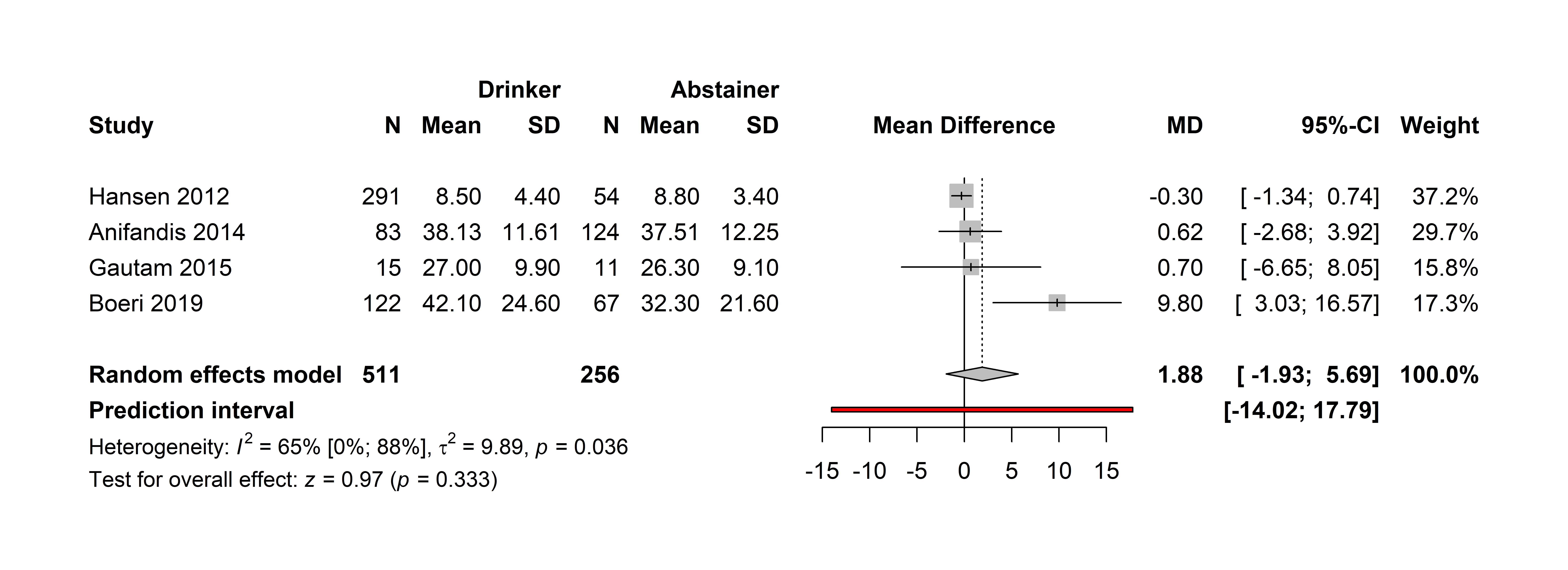


**Supplementary Figure 19.:** Comparison of drinkers’ and abstainers’ sperm DNA fragmentation values (continuous data)


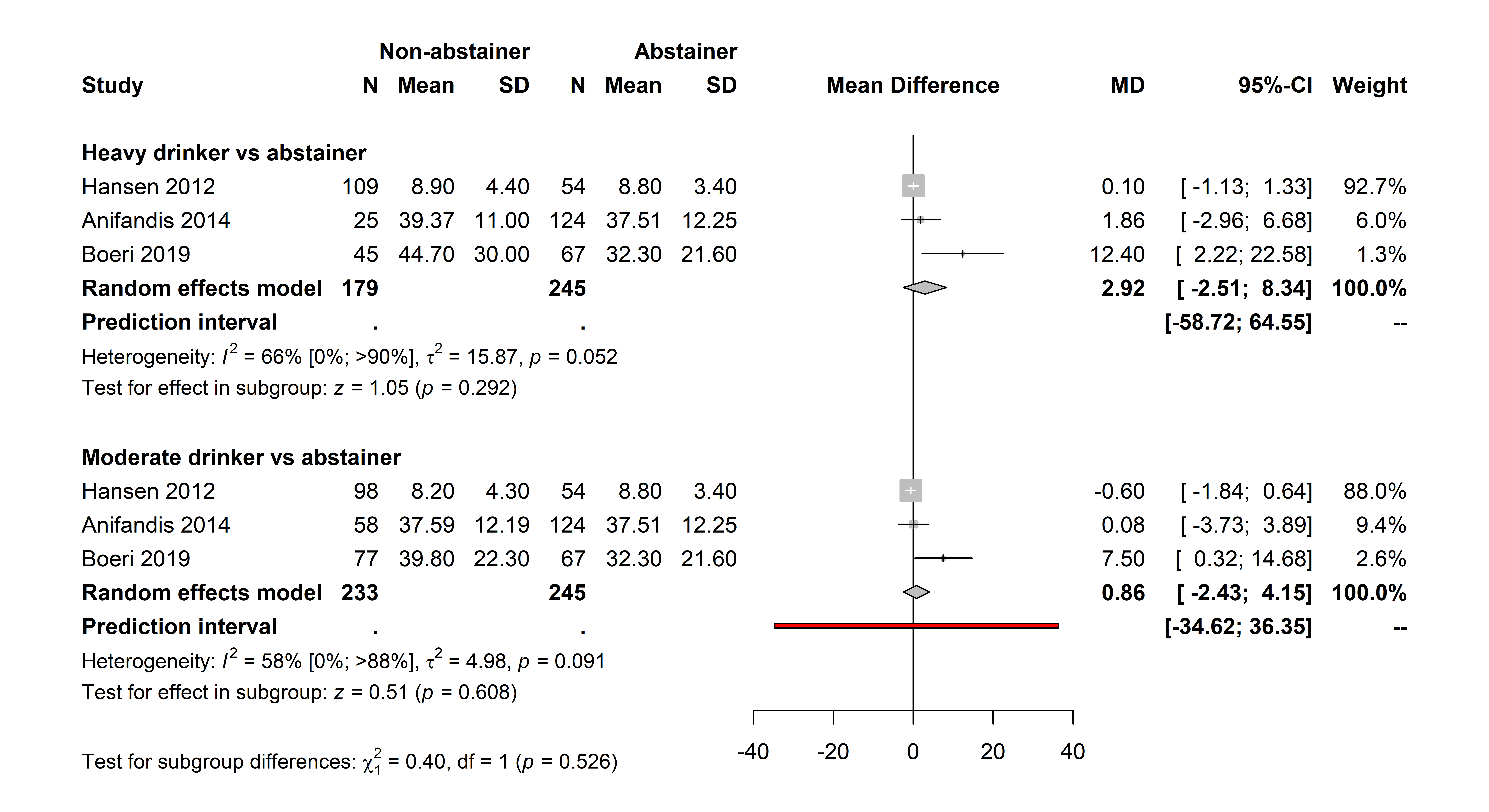


**Supplementary Figure 20.:** Comparison of heavy drinkers with abstainers, and moderate drinkers with abstainers’ sperm DNA fragmentation values (continuous data)

**

**

**Supplementary Figure 21.:** Comparison of sperm DNA fragmentation values of patients with different BMI categories (continuous data)





**Supplementary Figure 22.:** Comparison of sperm DNA fragmentation values measured via SCSA of patients with different BMI categories (continuous data)


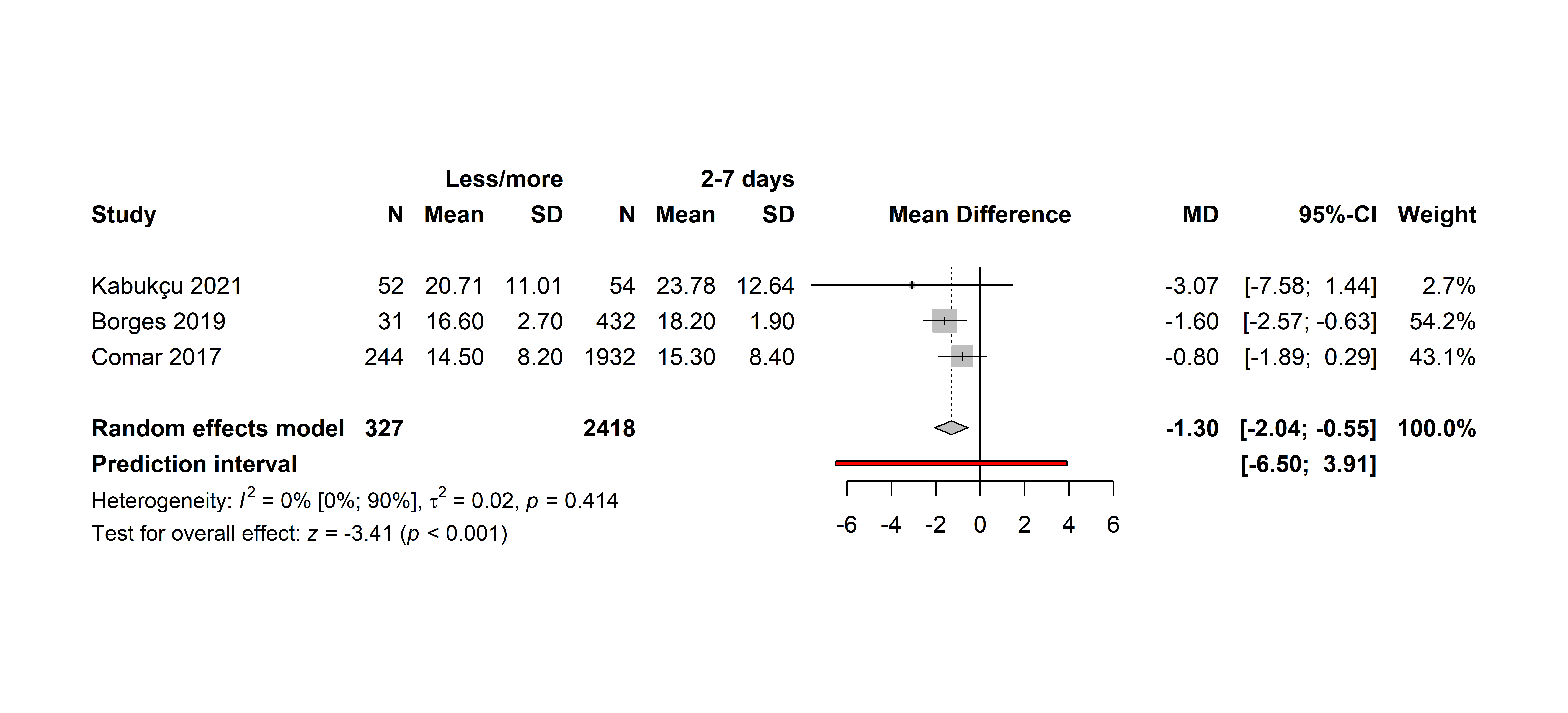


**Supplementary Figure 23.:** Comparison of patients’ sperm DNA fragmentation values after the “optimal” 2-7 days of sexual abstinence with abstinence times that differ from the “optimal” (continuous data, different subjects in the two groups)


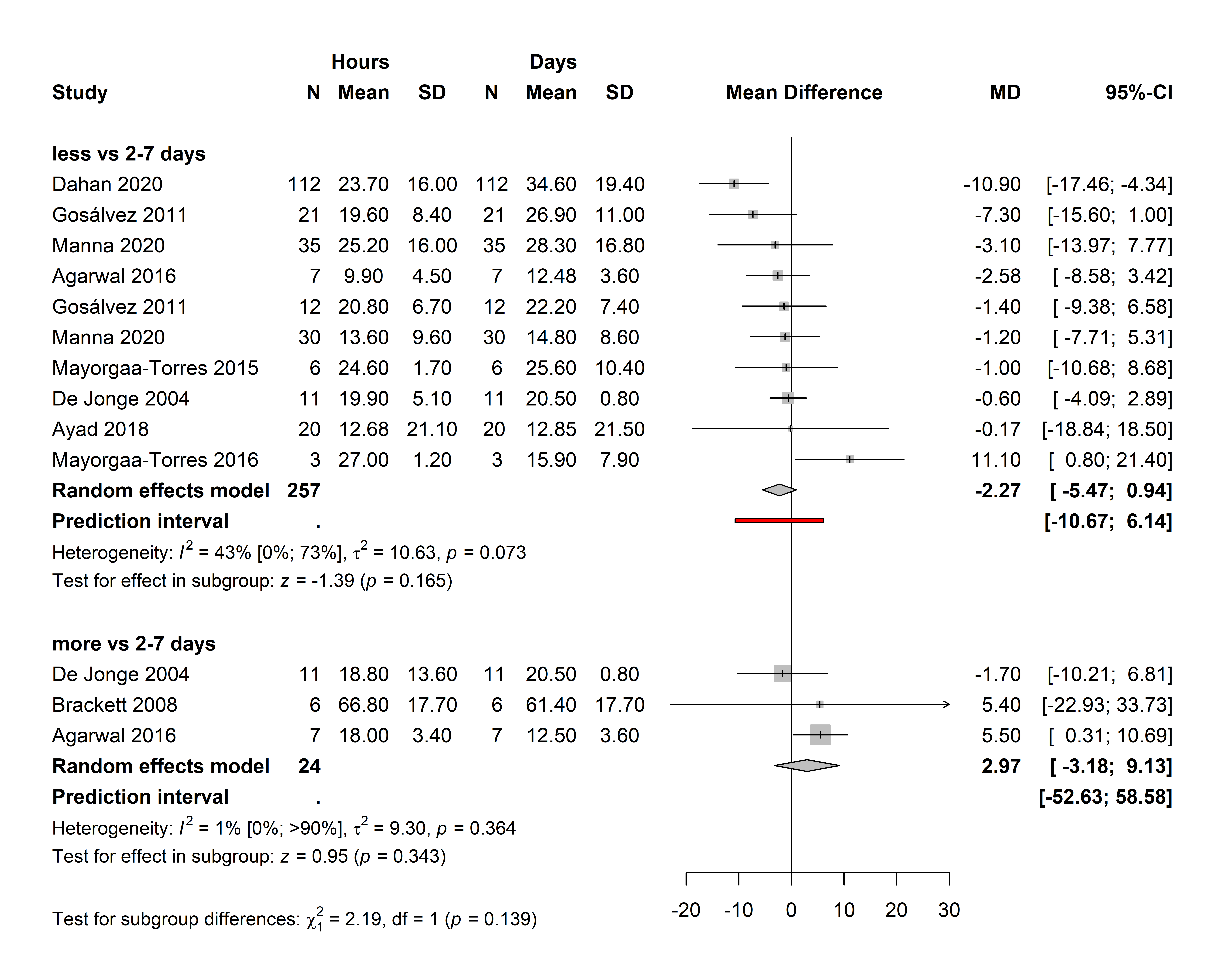


**Supplementary Figure 24.:** Comparison of patients’ sperm DNA fragmentation values after the “optimal” 2-7 days of sexual abstinence with abstinence times less than the “optimal” (continuous data, same subjects in the two groups)


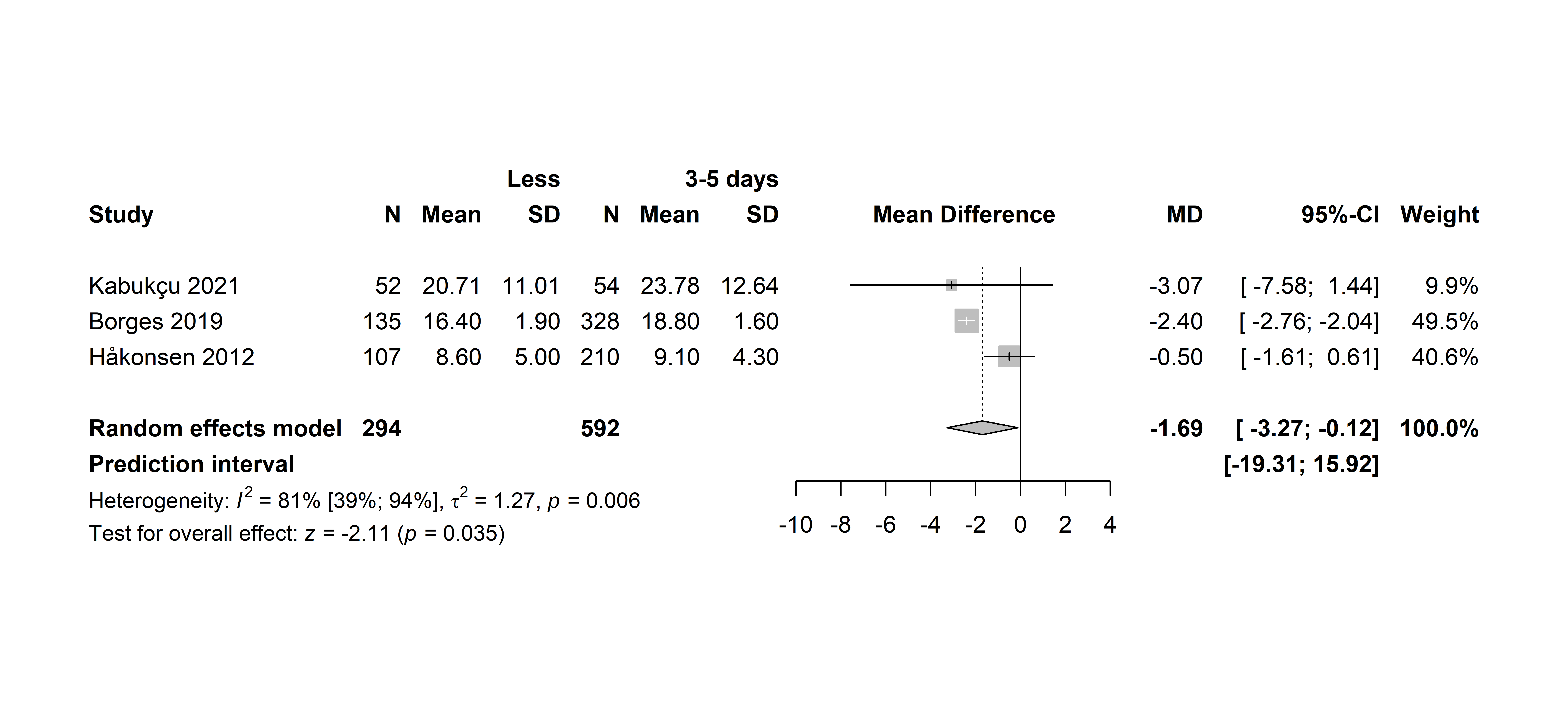


**Supplementary Figure 25.:** Comparison of patients’ sperm DNA fragmentation values after the “optimal” 3-5 days of sexual abstinence with abstinence times less than the “optimal” (continuous data, different subjects in the two groups)


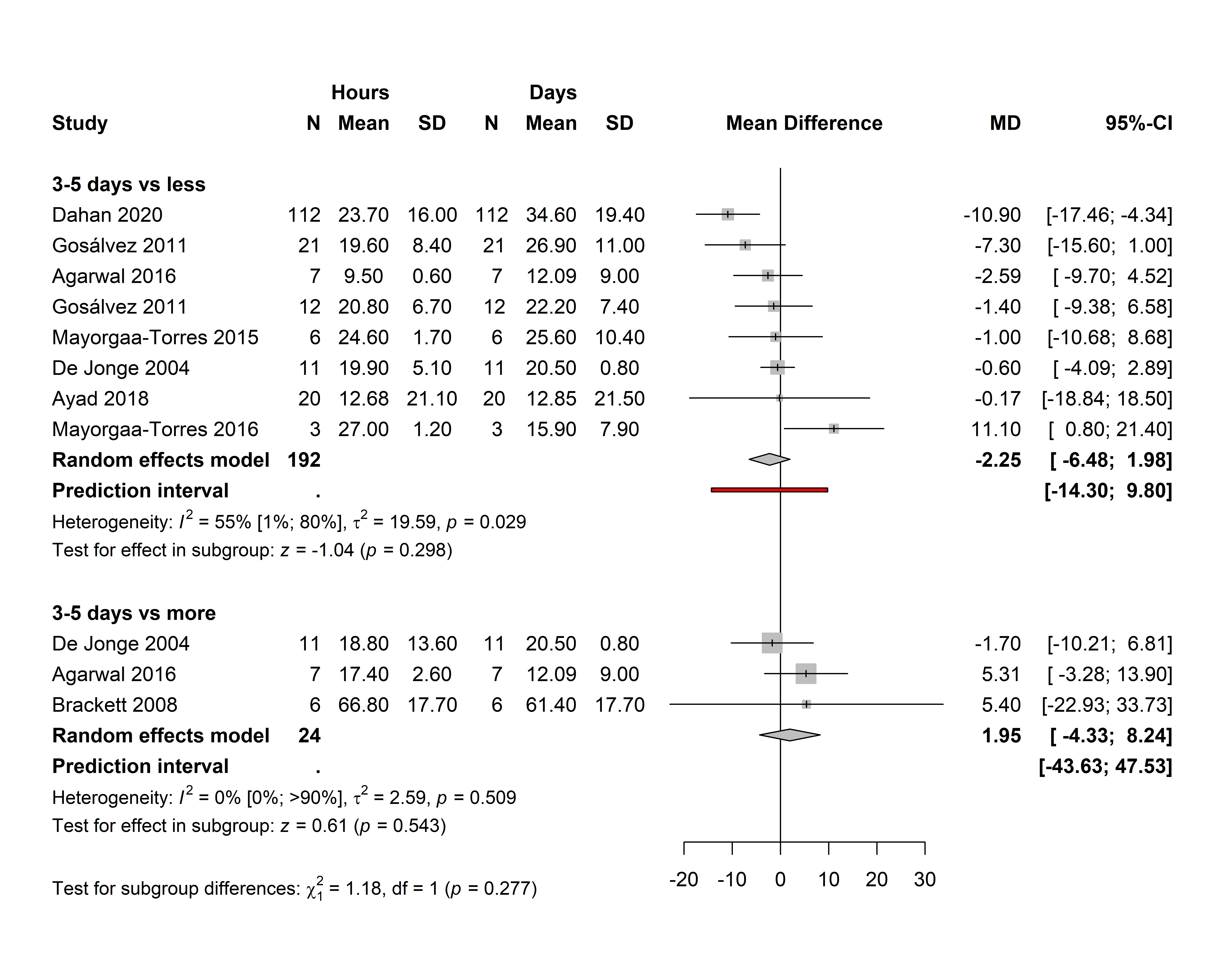


**Supplementary Figure 26.:** Comparison of patients’ sperm DNA fragmentation values after the “optimal” 3-5 days of sexual abstinence with abstinence times less than the “optimal” (continuous data, same subjects in the two groups)


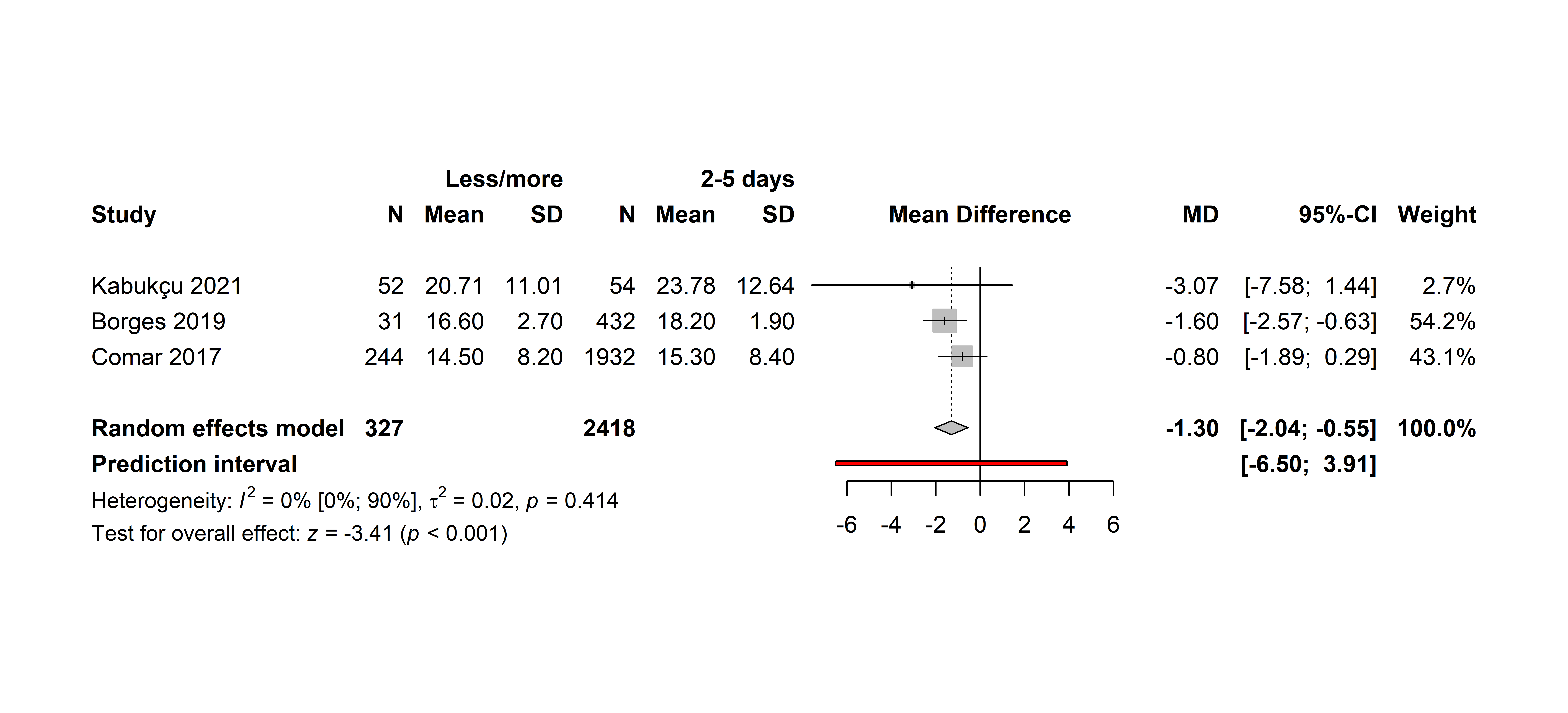


**Supplementary Figure 27.:** Comparison of patients’ sperm DNA fragmentation values after the “optimal” 2-5 days of sexual abstinence with abstinence times that differ from the “optimal” (continuous data, different subjects in the two groups)


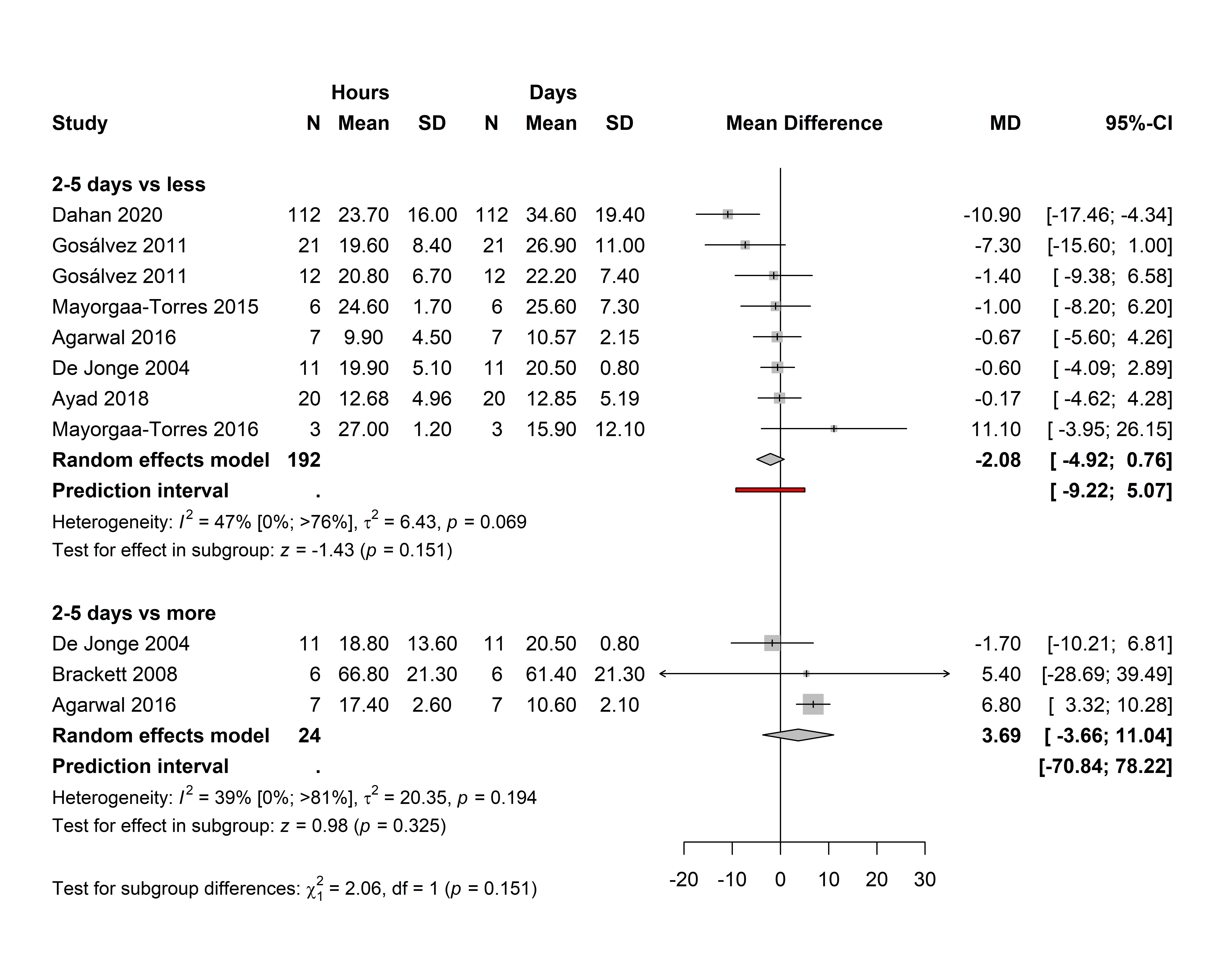


**Supplementary Figure 28.:** Comparison of patients’ sperm DNA fragmentation values after the “optimal” 2-5 days of sexual abstinence with abstinence times less than the “optimal” (continuous data, same subjects in the two groups)


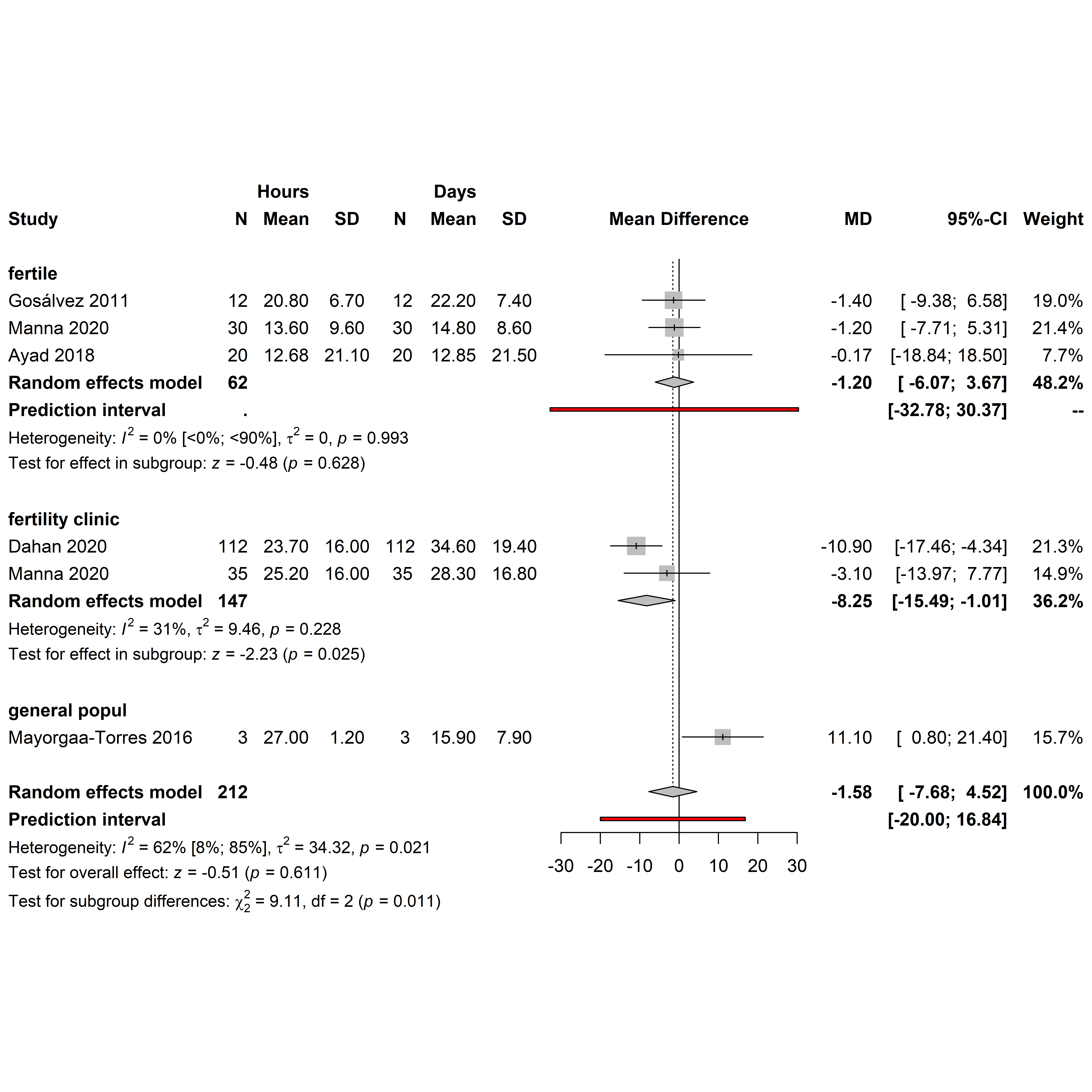


**Supplementary Figure 29.:** Comparison of patients’ sperm DNA fragmentation values after the “optimal” days of sexual abstinence with abstinence times of a few hours subdivided based on fertility status of the patients (continuous data, same subjects in the two groups)





**Supplementary Figure 30.:** Comparison of patients’ sperm DNA fragmentation values after the “optimal” 2-7 days of sexual abstinence with abstinence times less than the “optimal” subdivided based on sperm DNA fragmentation assay used (continuous data, same subjects in the two groups)


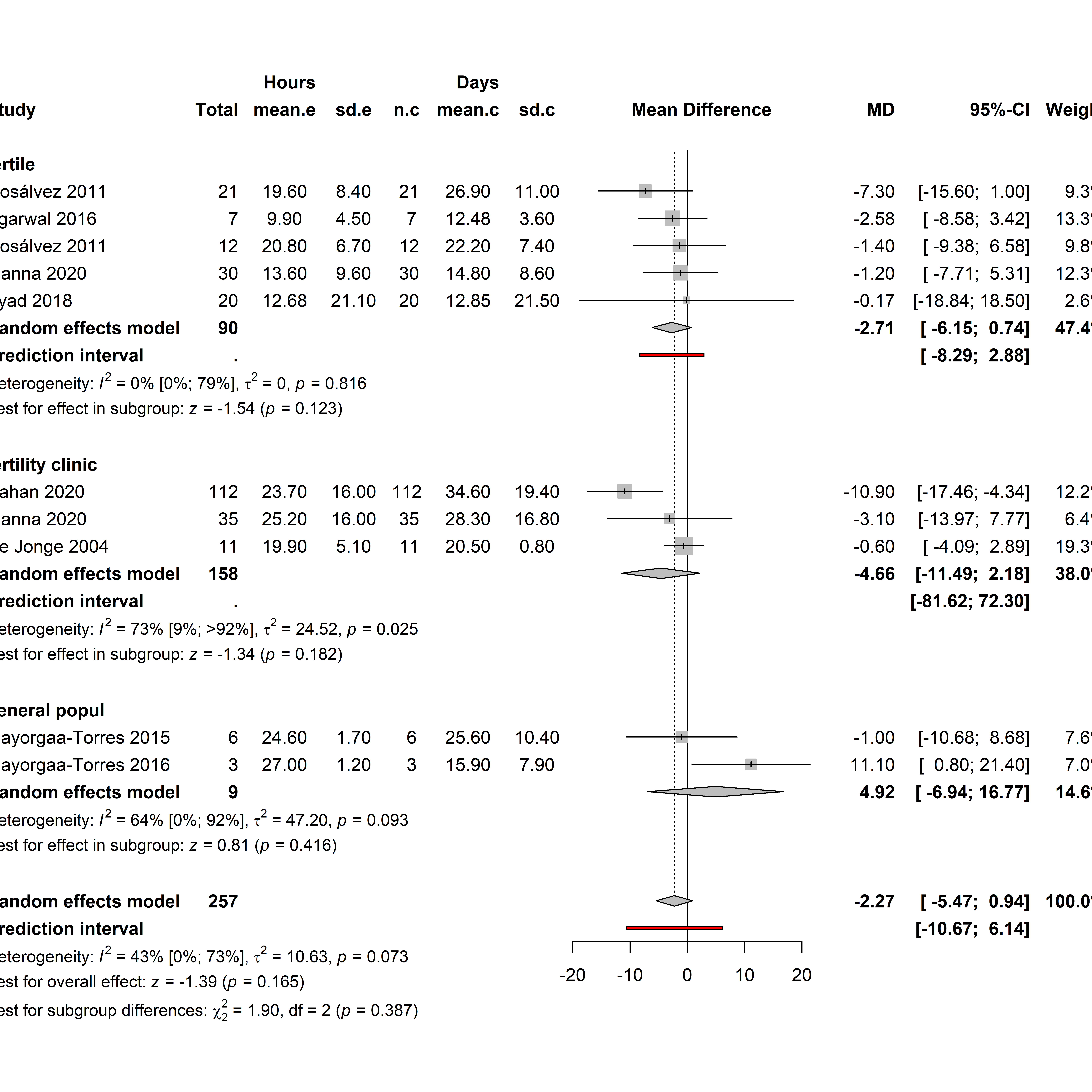


**Supplementary Figure 31.:** Comparison of patients’ sperm DNA fragmentation values after the “optimal” 2-7 days of sexual abstinence with abstinence times less than the “optimal” subdivided based on the fertility status of patients (continuous data, same subjects in the two groups)


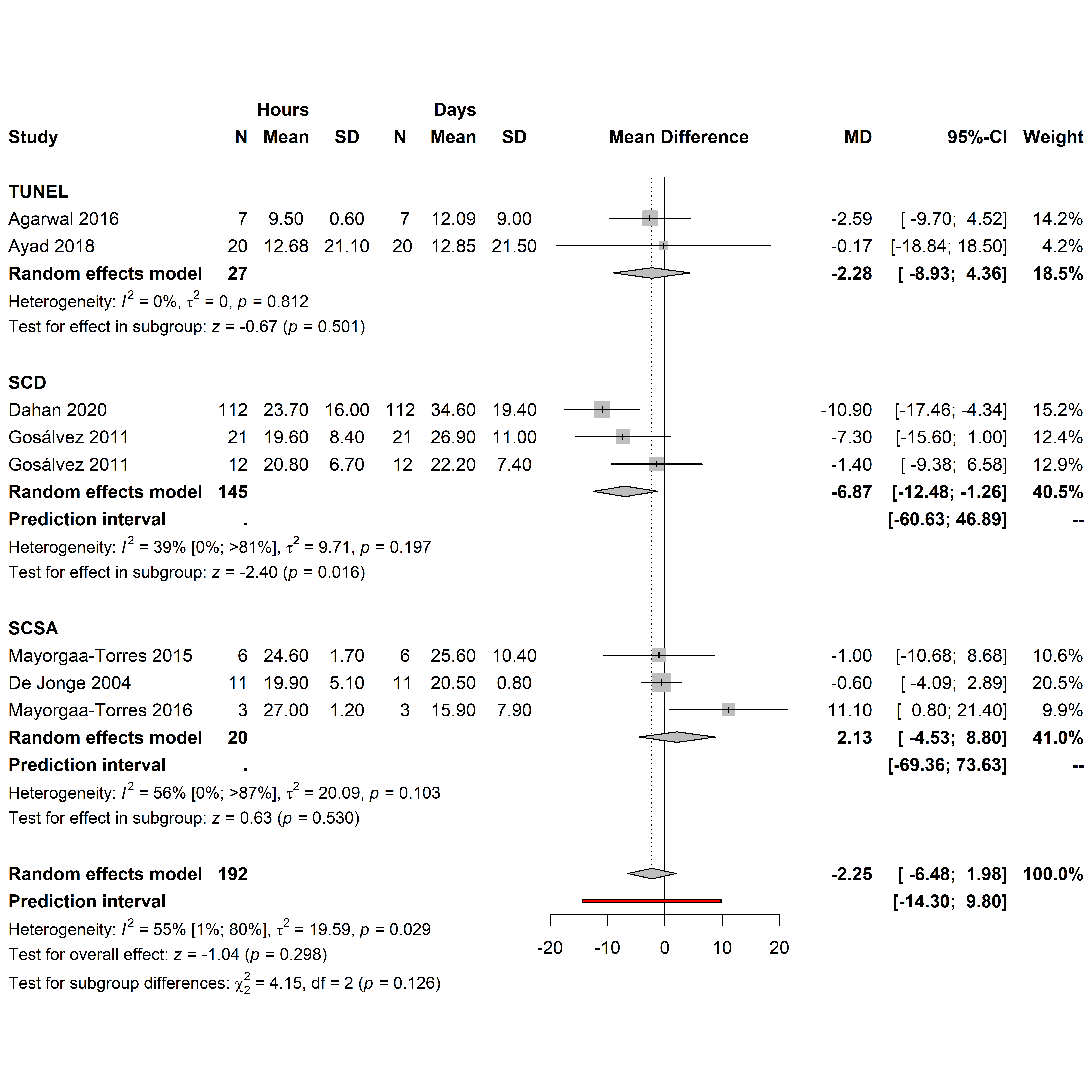


**Supplementary Figure 32.:** Comparison of patients’ sperm DNA fragmentation values after the “optimal” 3-5 days of sexual abstinence with abstinence times less than the “optimal” subdivided based on sperm DNA fragmentation assay used (continuous data, same subjects in the two groups)


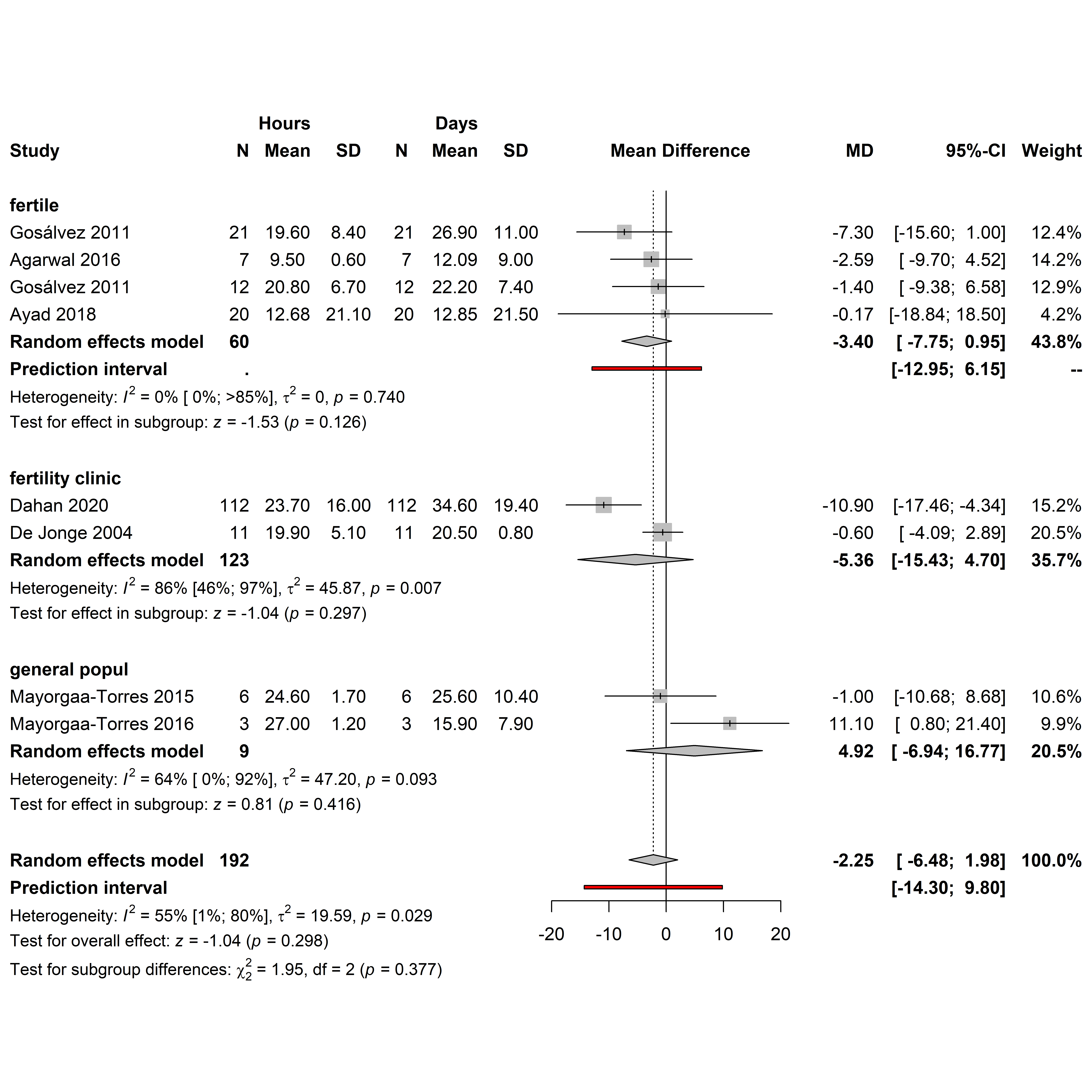


**Supplementary Figure 33.:** Comparison of patients’ sperm DNA fragmentation values after the “optimal” 3-5 days of sexual abstinence with abstinence times less than the “optimal” subdivided based on the fertility status of the patients (continuous data, same subjects in the two groups)


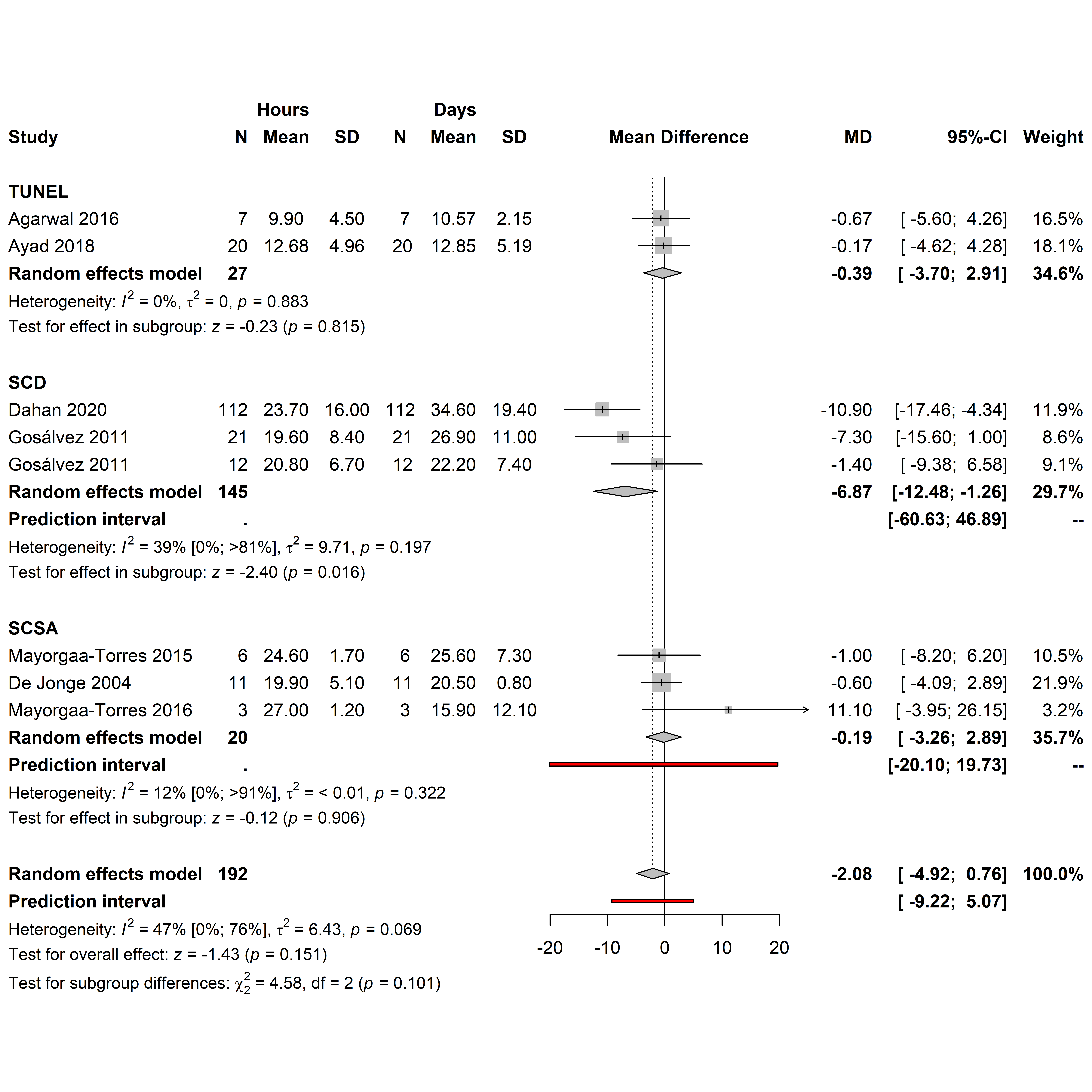


**Supplementary Figure 34.:** Comparison of patients’ sperm DNA fragmentation values after the “optimal” 2-5 days of sexual abstinence with abstinence times less than the “optimal” subdivided based on sperm DNA fragmentation assay used (continuous data, same subjects in the two groups)


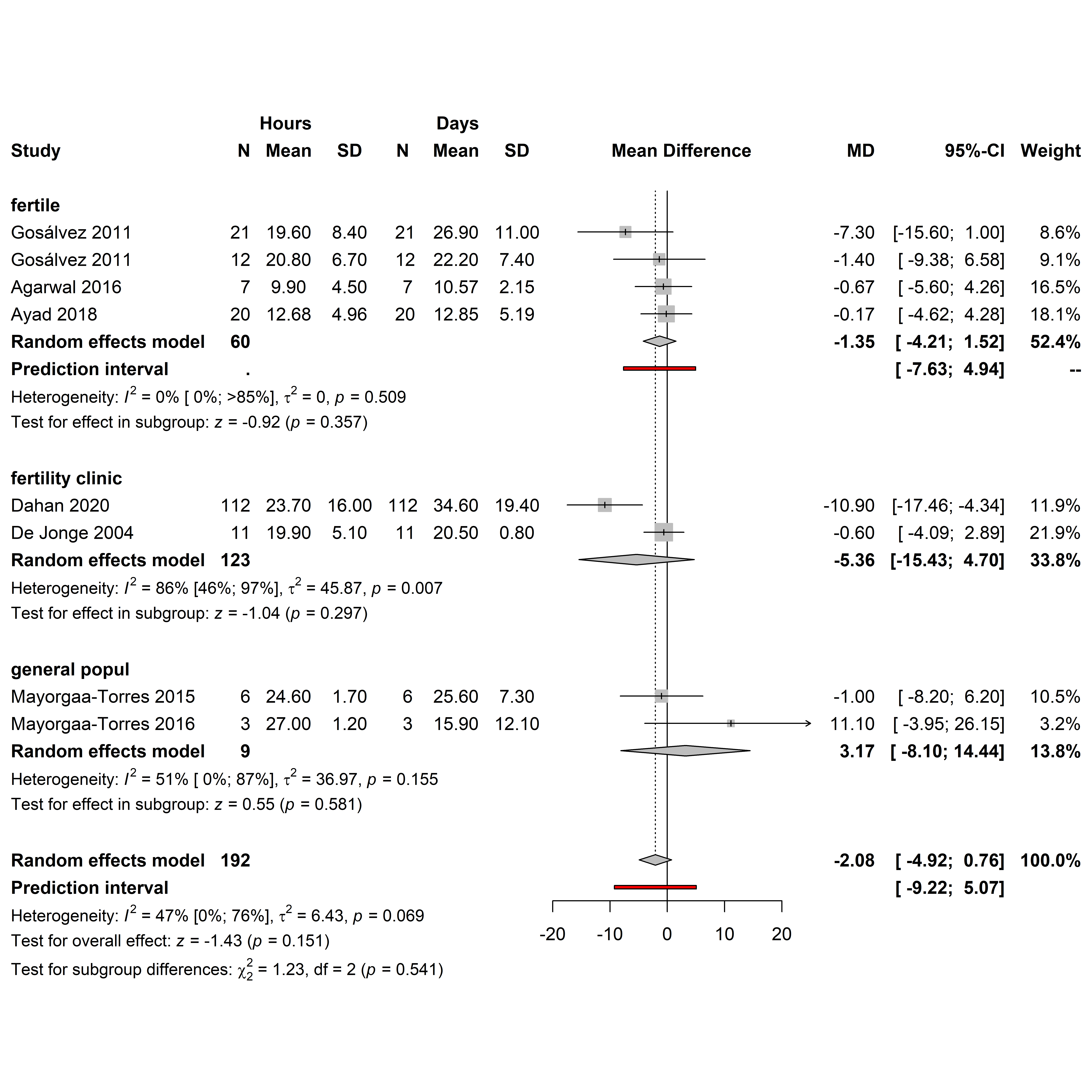


**Supplementary Figure 35.:** Comparison of patients’ sperm DNA fragmentation values after the “optimal” 3-5 days of sexual abstinence with abstinence times less than the “optimal” subdivided based on the fertility status of the patients (continuous data, same subjects in the two groups)

**Detailed results – other factors (paternal age, pollution, oxidative stress):**





**Supplementary Figure 36.:** Comparison of patients’ sperm DNA fragmentation values of different age groups (continuous data)


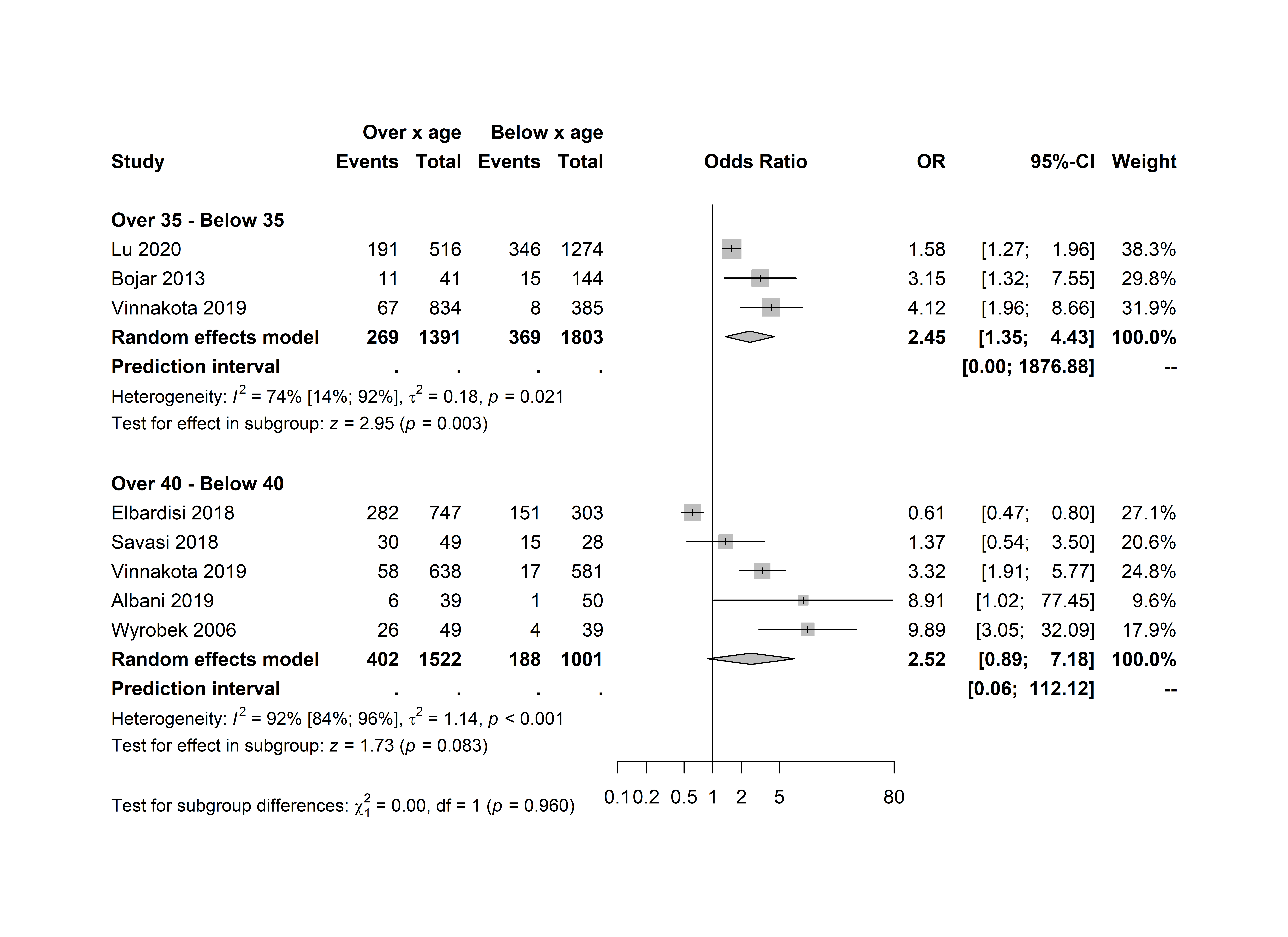


**Supplementary Figure 37.:** Comparison of patients’ sperm DNA fragmentation values of different age groups (continuous data)


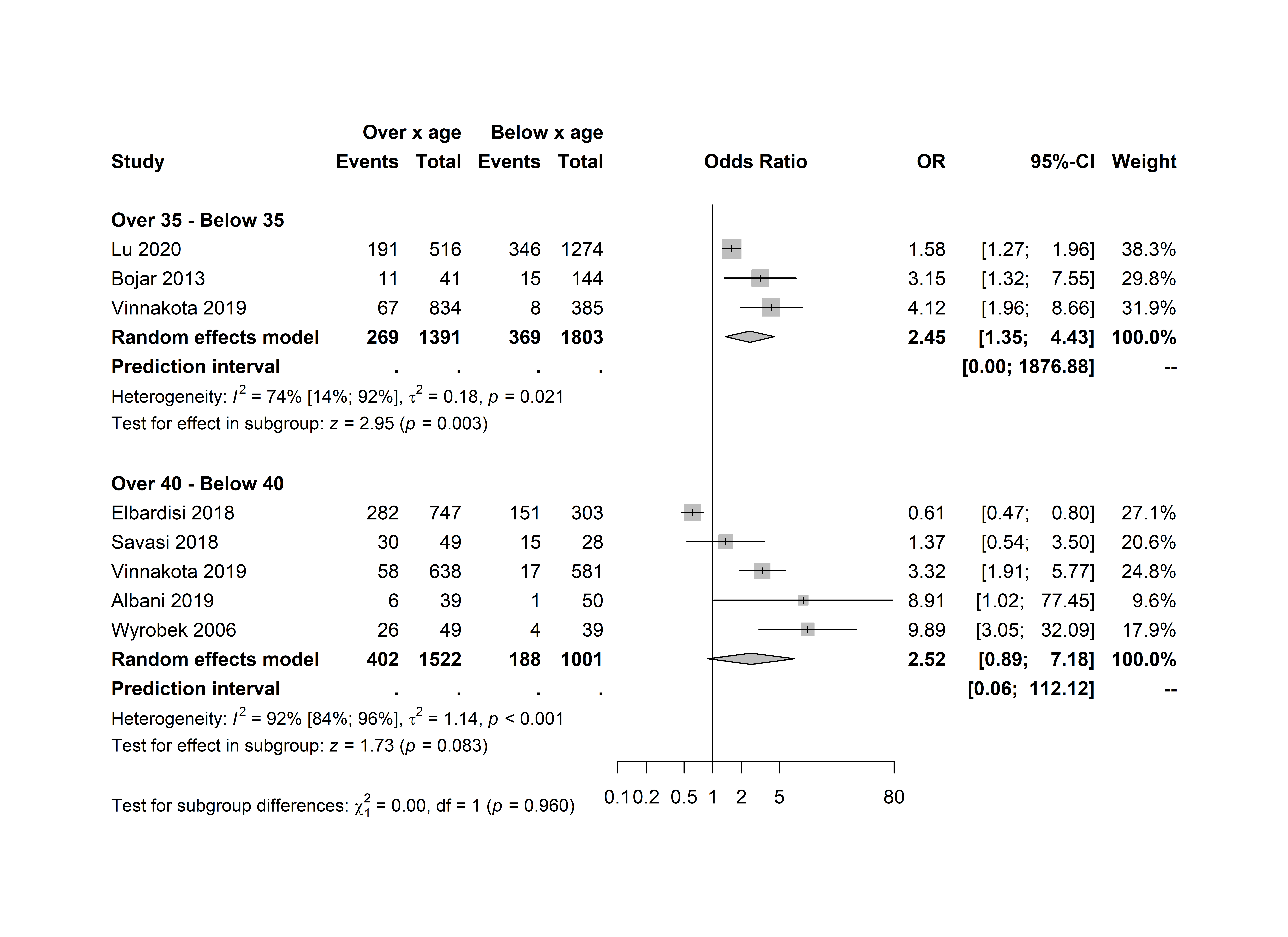


**Supplementary Figure 38.:** Comparison of patients’ sperm DNA fragmentation values of different age groups with cut-off values being at 30% DNA fragmentation


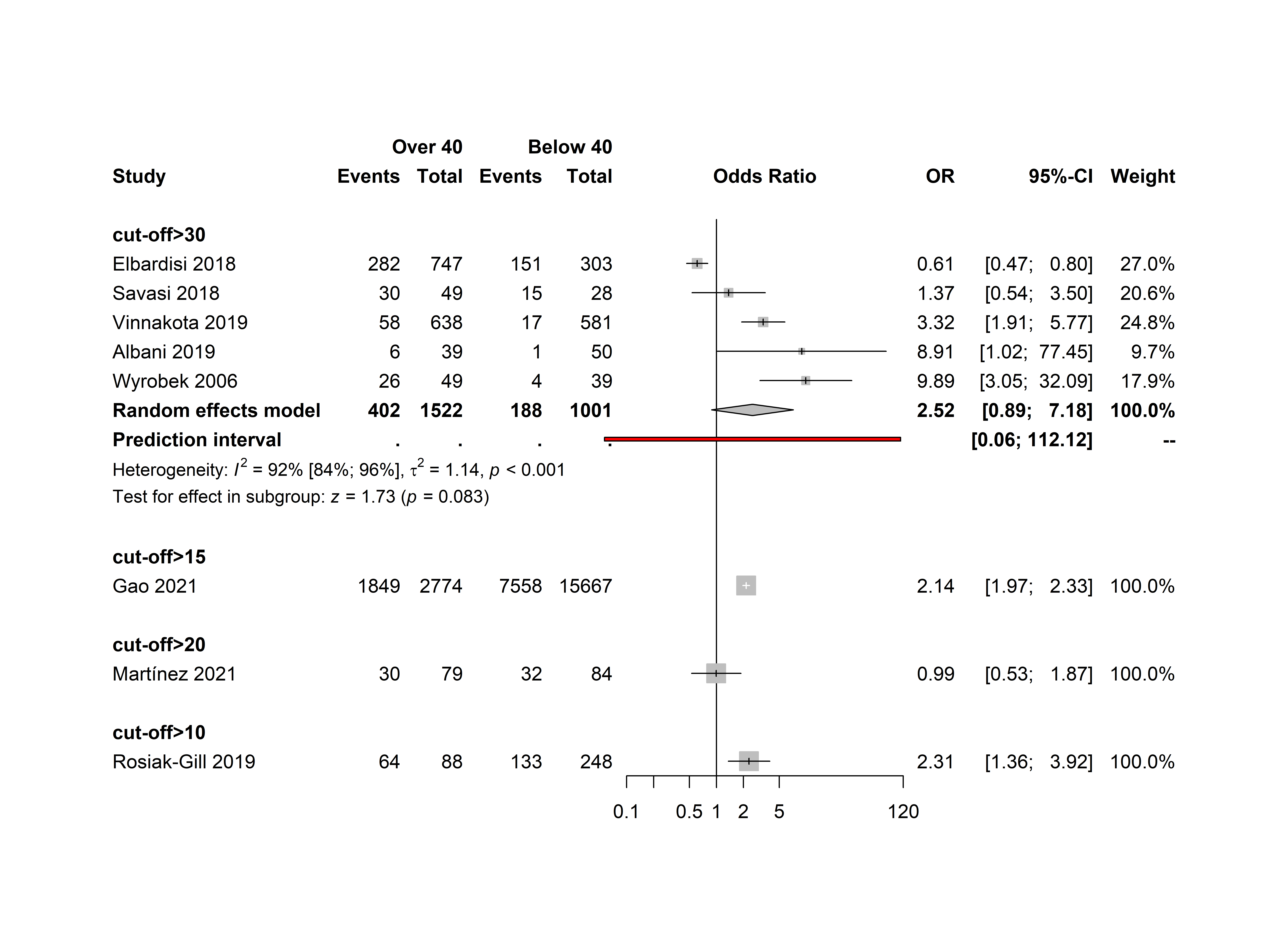


**Supplementary Figure 39.:** Comparison of patients’ sperm DNA fragmentation values of age groups over and below 40 years of age with different cut-off values for DNA fragmentation





**Supplementary Figure 40.:** Comparison of patients’ sperm DNA fragmentation values with more (experimental) or less (control) exposure to pollutants with subgroups based on the DNA fragmentation assay used (continuous data)





**Supplementary Figure 41.:** Comparison of patients’ sperm DNA fragmentation values with exposures to different types of pollutants (continuous data)


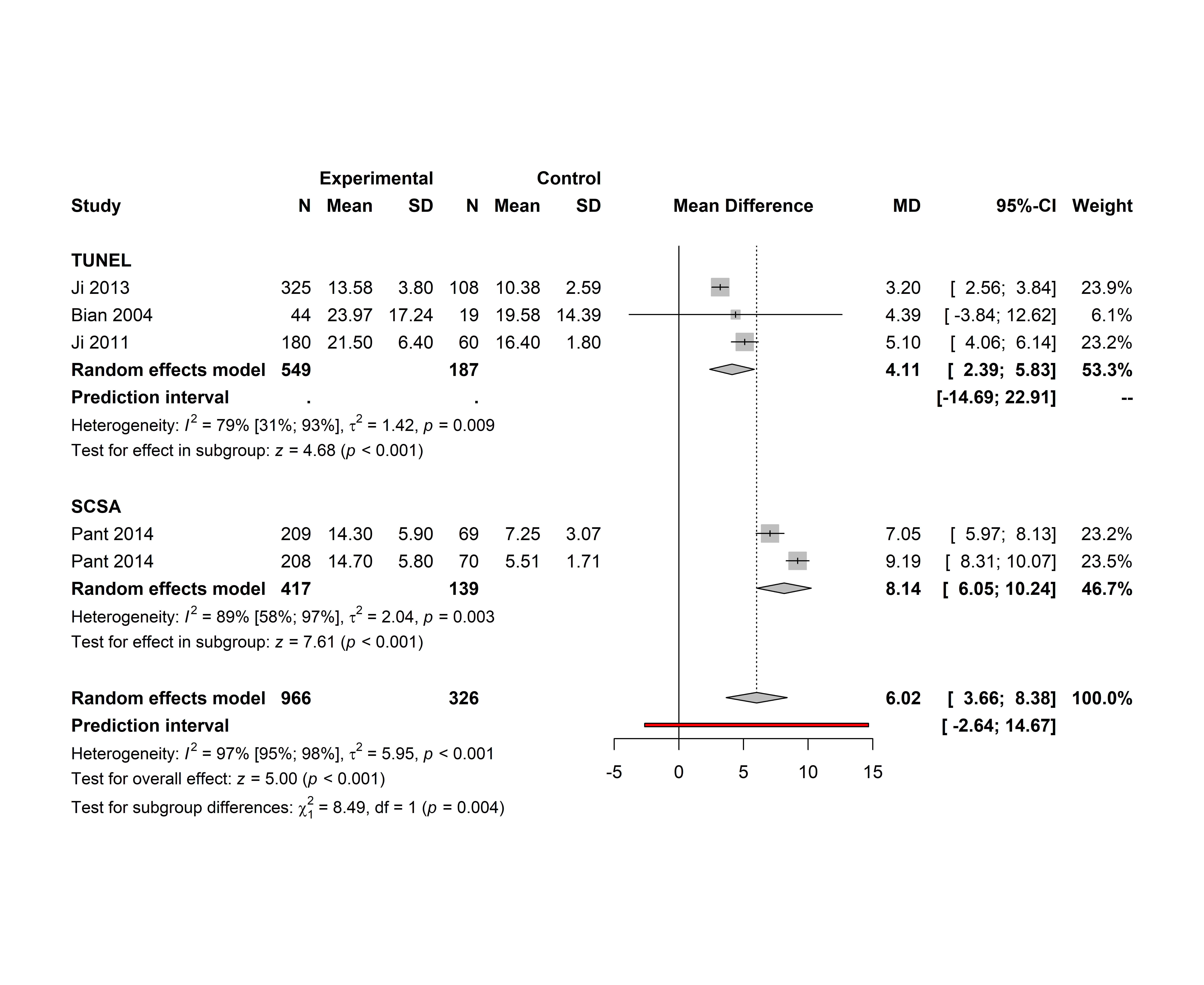


**Supplementary Figure 42.:** Comparison of patients’ sperm DNA fragmentation values with more (experimental) or less (control) exposure to pesticides or insecticides with subgroups based on the DNA fragmentation assay used (continuous data)


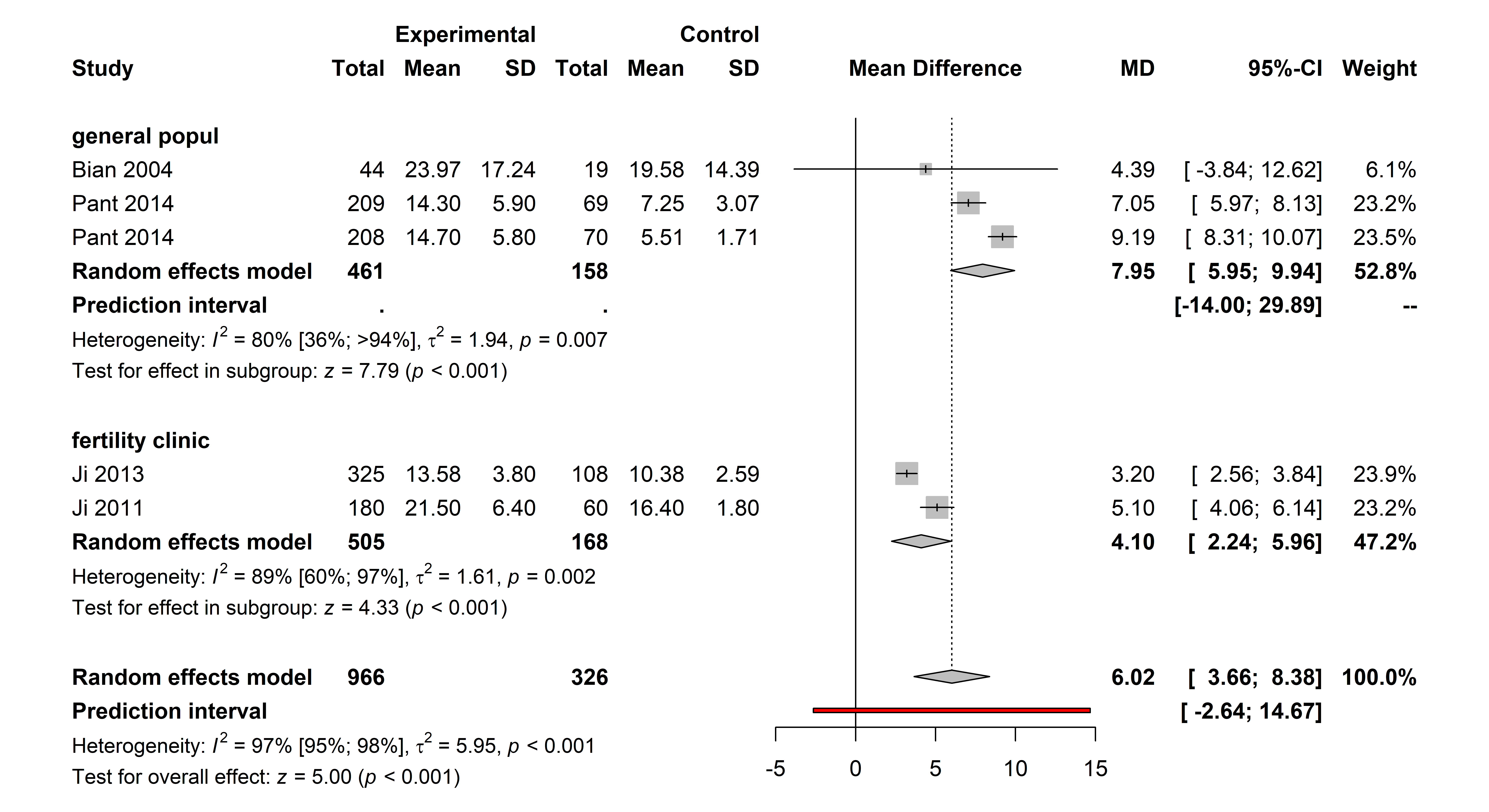


**Supplementary Figure 43.:** Comparison of patients’ sperm DNA fragmentation values with more (experimental) or less (control) exposure to pesticides or insecticides with subgroups based on the fertility status of patients (continuous data)





**Supplementary Figure 44.:** Comparison of patients’ sperm DNA fragmentation values with more or less reactive oxygen species (ROS) (continuous data)





**Supplementary Figure 45.:** Comparison of patients’ sperm DNA fragmentation values with more vs. less reactive oxygen species (ROS) – reduced model (continuous data)

**Supplementary Figure 46.:** Comparison of patients’ sperm DNA fragmentation values within the risk group and control group (continuous data)

**Supplementary Figure 47.:** Comparison of patients’ sperm DNA fragmentation values within the risk group and control group (continuous data, same subjects in the two groups)

**Supplementary Figure 48.:** Comparison of patients’ sperm DNA fragmentation values within the risk group and control group (cut-off values)

**REFERENCE**

1. Abdelbaki SA, Sabry JH, Al-Adl AM, Sabry HH. The impact of coexisting sperm DNA fragmentation and seminal oxidative stress on the outcome of varicocelectomy in infertile patients: a prospective controlled study. Arab journal of urology 2017;15:131‐139.

2. Abdullah N, Gilkey MS, Hunn C, Chen SH, Kavoussi KM, Esqueda AS, Wininger JD, et al. The impact of ipsilateral testicular atrophy on semen analysis and DNA fragmentation response to varicocele repair. Fertility and Sterility 2019;112:e412-e413.

3. Agarwal A, Gupta S, Du Plessis S, Sharma R, Esteves SC, Cirenza C, Eliwa J, et al. Abstinence Time and Its Impact on Basic and Advanced Semen Parameters. Urology 2016;94:102-110.

4. Agbaje IM, McVicar CM, Schock BC, McClure N, Atkinson AB, Rogers D, Lewis SEM. Increased concentrations of the oxidative DNA adduct 7,8-dihydro-8-oxo-2-deoxyguanosine in the germ-line of men with type 1 diabetes. Reproductive BioMedicine Online 2008;16:401-409.

5. Alargkof V, Kersten L, Stanislavov R, Kamenov Z, Nikolinakos P. Relationships between sperm DNA integrity and bulk semen parameters in Bulgarian patients with varicocele. Archivio Italiano di Urologia e Andrologia 2019;91:125-129.

6. Albani E, Castellano S, Gurrieri B, Arruzzolo L, Negri L, Borroni EM, Levi-Setti PE. Male age: negative impact on sperm DNA fragmentation. Aging 2019;11:2749-2761.

7. Alhathal N, San Gabriel M, Zini A. Beneficial effects of microsurgical varicocoelectomy on sperm maturation, DNA fragmentation, and nuclear sulfhydryl groups: A prospective trial. Andrology 2016;4:1204-1208.

8. Alshahrani S, Agarwal A, Assidi M, Abuzenadah AM, Durairajanayagam D, Ayaz A, Sharma R, et al. Infertile men older than 40 years are at higher risk of sperm DNA damage. Reproductive Biology and Endocrinology 2014;12.

9. Ammar O, Tekeya O, Hannachi I, Sallem A, Haouas Z, Mehdi M. Increased Sperm DNA Fragmentation in Infertile Men with Varicocele: Relationship with Apoptosis, Seminal Oxidative Stress, and Spermatic Parameters. Reproductive Sciences 2021;28:909-919.

10. Amor H, Nyaz S, Hammadeh ME. Paternal smoking in relation to sperm quality and intracytoplasmic sperm injection outcomes. International Journal of Women's Health and Reproduction Sciences 2019;7:451-460.

11. Andersen JM, Rønning PO, Herning H, Bekken SD, Haugen TB, Witczak O. Fatty acid composition of spermatozoa is associated with BMI and with semen quality. Andrology 2016;4:857-865.

12. Anifandis G, Bounartzi T, Messini CI, Dafopoulos K, Sotiriou S, Messinis IE. The impact of cigarette smoking and alcohol consumption on sperm parameters and sperm DNA fragmentation (SDF) measured by Halosperm®. Archives of Gynecology and Obstetrics 2014;290:777-782.

13. Ayad BM, Van der Horst G, du Plessis SS. Short abstinence: A potential strategy for the improvement of sperm quality. Middle East Fertility Society Journal 2018;23:19-22.

14. Bandel I, Bungum M, Richtoff J, Malm J, Axelsson J, Pedersen HS, Ludwicki JK, et al. No association between body mass index and sperm DNA integrity. Human Reproduction 2015;30:1704-1713.

15. Banks N, Sun F, Krawetz SA, Coward RM, Masson P, Smith JF, Trussell JC, et al. Male vitamin D status and male factor infertility. Fertil Steril 2021;116:973-979.

16. Belloc S, Benkhalifa M, Junca AM, Dumont M, Bacrie PC, Ménézo Y. Paternal age and sperm DNA decay: Discrepancy between chromomycin and aniline blue staining. Reproductive BioMedicine Online 2009;19:264-269.

17. Berg E, Houska P, Nesheim N, Schuppe HC, Pilatz A, Fijak M, Manthey M, et al. Chronic prostatitis/chronic pelvic pain syndrome leads to impaired semen parameters, increased sperm DNA fragmentation and unfavorable changes of sperm protamine mrna ratio. International Journal of Molecular Sciences 2021;22.

18. Bergamo P, Volpe MG, Lorenzetti S, Mantovani A, Notari T, Cocca E, Cerullo S, et al. Human semen as an early, sensitive biomarker of highly polluted living environment in healthy men: A pilot biomonitoring study on trace elements in blood and semen and their relationship with sperm quality and RedOx status. Reprod Toxicol 2016;66:1-9.

19. Bian Q, Xu LC, Wang SL, Xia YK, Tan LF, Chen JF, Song L, et al. Study on the relation between occupational fenvalerate exposure and spermatozoa DNA damage of pesticide factory workers. Occupational and Environmental Medicine 2004;61:999-1005.

20. Boeri L, Capogrosso P, Cazzaniga W, Pozzi E, Candela L, Belladelli F, Oreggia D, et al. SHBG levels in primary infertile men: A critical interpretation in clinical practice. Endocrine Connections 2020;9:658-666.

21. Boeri L, Capogrosso P, Ventimiglia E, Pederzoli F, Cazzaniga W, Chierigo F, Deho F, et al. Heavy cigarette smoking and alcohol consumption are associated with impaired sperm parameters in primary infertile men. Asian Journal of Andrology 2019;21:478-485.

22. Boeri L, Capogrosso P, Ventimiglia E, Pederzoli F, Frego N, Cazzaniga W, Chierigo F, et al. Undiagnosed prediabetes is highly prevalent in primary infertile men – results from a cross-sectional study. BJU International 2019;123:1070-1077.

23. Bojar I, Witczak M, Wdowiak A. Biological and environmental conditionings for sperm DNA fragmentation. Annals of Agricultural and Environmental Medicine 2013;20:865-868.

24. Borges E, Braga DPAF, Zanetti BF, Iaconelli A, Setti AS. Revisiting the impact of ejaculatory abstinence on semen quality and intracytoplasmic sperm injection outcomes. Andrology 2019;7:213-219.

25. Bosco L, Notari T, Ruvolo G, Roccheri MC, Martino C, Chiappetta R, Carone D, et al. Sperm DNA fragmentation: An early and reliable marker of air pollution. Environmental Toxicology and Pharmacology 2018;58:243-249.

26. Bozhedomov VA, Shomarufov AB, Bozhedomova GE, D OA, Kamalov DM, Sorokin NI, Kamalov AA. Varicocele and reproductive function: pathozoospermia treatment (a prospective comparative study). Urologiia (Moscow, Russia : 1999) 2021:62-68.

27. Brackett NL, Ibrahim E, Grotas JA, Aballa TC, Lynne CM. Higher sperm DNA damage in semen from men with spinal cord injuries compared with controls. Journal of Andrology 2008;29:93-99.

28. Brahem S, Mehdi M, Elghezal H, Saad A. The effects of male aging on semen quality, sperm DNA fragmentation and chromosomal abnormalities in an infertile population. Journal of Assisted Reproduction and Genetics 2011;28:425-432.

29. Chavarro JE, Toth TL, Wright DL, Meeker JD, Hauser R. Body mass index in relation to semen quality, sperm DNA integrity, and serum reproductive hormone levels among men attending an infertility clinic. Fertility and Sterility 2010;93:2222-2231.

30. Chigrinets SV, Brukhin GV. The risk of sperm demage in men with the combined effect of endocrine disruptors. Vestnik Rossiiskoi Akademii Meditsinskikh Nauk 2019;74:229-234.

31. Comar VA, Petersen CG, Mauri AL, Mattila M, Vagnini LD, Renzi A, Petersen B, et al. Influence of the abstinence period on human sperm quality: Analysis of 2,458 semen samples. Jornal Brasileiro de Reproducao Assistida 2017;21:306-312.

32. Cortés-Gutiérrez EI, Dávila-Rodríguez MI, Fernández JL, de la O-Pérez LO, Garza-Flores ME, Eguren–Garza R, Gosálvez J. The presence of human papillomavirus in semen does not affect the integrity of sperm DNA. Andrologia 2017;49.

33. Cui X, Jing X, Wu X, Wang Z, Li Q. Potential effect of smoking on semen quality through DNA damage and the downregulation of Chk1 in sperm. Molecular Medicine Reports 2016;14:753-761.

34. Dahan MH, Mills G, Khoudja R, Gagnon A, Tan G, Tan SL. Three hour abstinence as a treatment for high sperm DNA fragmentation: a prospective cohort study. Journal of Assisted Reproduction and Genetics 2021;38:227-233.

35. Darbandi M, Darbandi S, Agarwal A, Baskaran S, Dutta S, Sengupta P, Khorram Khorshid HR, et al. Reactive oxygen species-induced alterations in H19-Igf2 methylation patterns, seminal plasma metabolites, and semen quality. Journal of Assisted Reproduction and Genetics 2019;36:241-253.

36. Darbandi S, Darbandi M, Khorshid HRK, Sadeghi MR, Heidari M, Cheshmi G, Akhondi MM. The effect of paternal age on semen quality and fertilization outcome in men with normal sperm DNA compaction, reactive oxygen species, and total antioxidant capacity levels. Turk J Urol 2019;45:164-170.

37. Das M, Al-Hathal N, San-Gabriel M, Phillips S, Kadoch IJ, Bissonnette F, Holzer H, et al. High prevalence of isolated sperm DNA damage in infertile men with advanced paternal age. Journal of Assisted Reproduction and Genetics 2013;30:843-848.

38. De Jonge C, Lafromboise M, Bosmans E, Ombelet W, Cox A, Nijs M. Influence of the abstinence period on human sperm quality. Fertility and Sterility 2004;82:57-65.

39. De Win G, De Neubourg D, De Wachter S, Vaganée D, Punjabi U. Peak retrograde flow a potential objective management tool to identify young adults with varicocele ‘at risk’ for a high sperm DNA fragmentation. Journal of Pediatric Urology 2021.

40. Dehghan Marvast L, Talebi AR, Ghasemzadeh J, Hosseini A, Pacey AA. Effects of Chlamydia trachomatis infection on sperm chromatin condensation and DNA integrity. Andrologia 2018;50.

41. Depuydt C, Donders G, Verstraete L, Beert J, Salembier G, Bosmans E, Dhont N, et al. Negative impact of elevated dna fragmentation and human papillomavirus (Hpv) presence in sperm on the outcome of intra-uterine insemination (iui). Journal of Clinical Medicine 2021;10:1-14.

42. Dieamant F, Petersen CG, Mauri AL, Conmar V, Mattila M, Vagnini LD, Renzi A, et al. Semen parameters in men with varicocele: DNA fragmentation, chromatin packaging, mitochondrial membrane potential, and apoptosis. Jornal Brasileiro de Reproducao Assistida 2017;21:295-301.

43. Domes T, Lo KC, Grober ED, Mullen JBM, Mazzulli T, Jarvi K. The incidence and effect of bacteriospermia and elevated seminal leukocytes on semen parameters. Fertility and Sterility 2012;97:1050-1055.

44. Dupont C, Faure C, Sermondade N, Boubaya M, Eustache F, Clément P, Briot P, et al. Obesity leads to higher risk of sperm DNA damage in infertile patients. Asian Journal of Andrology 2013;15:622-625.

45. Eini F, Kutenaei MA, Zareei F, Dastjerdi ZS, Shirzeyli MH, Salehi E. Effect of bacterial infection on sperm quality and DNA fragmentation in subfertile men with Leukocytospermia. BMC Molecular and Cell Biology 2021;22.

46. Eisenberg ML, Kim S, Chen Z, Sundaram R, Schisterman EF, Buck Louis GM. The relationship between male BMI and waist circumference on semen quality: Data from the LIFE study. Human Reproduction 2014;29:193-200.

47. Elbardisi H, Arafa M, Singh N, Betts B, Agrawal A, Henkel R, Al-Hadi AA, et al. The effect of paternal age on intracytoplasmic sperm injection outcome in unexplained infertility. Arab Journal of Urology 2021;19:274-280.

48. Elbardisi H, Finelli R, Agarwal A, Majzoub A, Henkel R, Arafa M. Predictive value of oxidative stress testing in semen for sperm DNA fragmentation assessed by sperm chromatin dispersion test. Andrology 2020;8:610-617.

49. Elbardisi H, Majzoub A, Al Said S, Al Rumaihi K, El Ansari W, Alattar A, Arafa M. Geographical differences in semen characteristics of 13 892 infertile men. Arab Journal of Urology 2018;16:3-9.

50. Elshal MF, El-Sayed IH, Elsaied MA, El-Masry SA, Kumosani TA. Sperm head defects and disturbances in spermatozoal chromatin and DNA integrities in idiopathic infertile subjects: Association with cigarette smoking. Clinical Biochemistry 2009;42:589-594.

51. Esfahani MHN, Abbasi H, Mirhosseini Z, Ghasemi N, Razavi S, Tavalaee M, Tanhaei S, et al. Can altered expression of HSPA2 in varicocele patients lead to abnormal spermatogenesis? International Journal of Fertility and Sterility 2010;4:104-113.

52. Esteves SC, Gosálvez J, López-Fernández C, Núñez-Calonge R, Caballero P, Agarwal A, Fernández JL. Diagnostic accuracy of sperm DNA degradation index (DDSi) as a potential noninvasive biomarker to identify men with varicocele-associated infertility. International Urology and Nephrology 2015;47:1471-1477.

53. Evenson DP, Djira G, Kasperson K, Christianson J. Relationships between the age of 25,445 men attending infertility clinics and sperm chromatin structure assay (SCSA®) defined sperm DNA and chromatin integrity. Fertility and Sterility 2020;114:311-320.

54. Falahieh FM, Zarabadipour M, Mirani M, Abdiyan M, Dinparvar M, Alizadeh H, Paktinat S, et al. Effects of moderate COVID-19 infection on semen oxidative status and parameters 14 and 120 days after diagnosis. Reproduction, Fertility and Development 2021.

55. Fernandez-Encinas A, García-Peiró A, Del Rey J, Ribas-Maynou J, Abad C, Amengual MJ, Prada E, et al. Proteomic analysis in seminal plasma of fertile donors and infertile patients with sperm dna fragmentation. International Journal of Molecular Sciences 2020;21:2-15.

56. Finelli R, Pallotti F, Cargnelutti F, Faja F, Carlini T, Rizzo F, Lenzi A, et al. Sperm DNA damage and cytokines in varicocele: A case-control study. Andrologia 2021;53.

57. Frainais C, Vialard F, Rougier N, Aegerther P, Damond F, Ayel JP, Yazbeck C, et al. Impact of freezing/thawing technique on sperm DNA integrity in HIV-1 patients. Journal of assisted reproduction and genetics 2010;27:415‐421.

58. Gallegos G, Ramos B, Santiso R, Goyanes V, Gosálvez J, Fernández JL. Sperm DNA fragmentation in infertile men with genitourinary infection by Chlamydia trachomatis and Mycoplasma. Fertility and Sterility 2008;90:328-334.

59. Gao J, Duan YG, Yi X, Yeung WSB, Ng EHY. A randomised trial comparing conventional semen parameters, sperm DNA fragmentation levels and satisfaction levels between semen collection at home and at the clinic. Andrologia 2020;52:e13628.

60. Gao J, Yuan R, Yang S, Wang Y, Huang Y, Yan L, Jiang H, et al. Age-related changes in human conventional semen parameters and sperm chromatin structure assay-defined sperm DNA/chromatin integrity. Reproductive BioMedicine Online 2021;42:973-982.

61. García-Ferreyra J, Luna D, Villegas L, Romero R, Zavala P, Hilario R, Dueñas-Chacón J. High Aneuploidy Rates Observed in Embryos Derived from Donated Oocytes are Related to Male Aging and High Percentages of Sperm DNA Fragmentation. Clin Med Insights Reprod Health 2015;9:21-27.

62. García-Peiró A, Martínez-Heredia J, Oliver-Bonet M, Abad C, Amengual MJ, Navarro J, Jones C, et al. Protamine 1 to protamine 2 ratio correlates with dynamic aspects of DNA fragmentation in human sperm. Fertility and Sterility 2011;95:105-109.

63. García-Peiró A, Ribas-Maynou J, Oliver-Bonet M, Navarro J, Checa MA, Nikolaou A, Amengual MJ, et al. Multiple determinations of sperm DNA fragmentation show that varicocelectomy is not indicated for infertile patients with subclinical varicocele. BioMed Research International 2014;2014.

64. Gautam S, Chawla B, Kumar SB, Bisht S, Dada R. Sperm DNA damage in non-familial sporadic heritable retinoblastoma (NFSHRb). Clinical Epidemiology and Global Health 2015;3:S20-S25.

65. Ghandehari-Alavijeh R, Tavalaee M, Zohrabi D, Foroozan-Broojeni S, Abbasi H, Nasr-Esfahani MH. Hypoxia pathway has more impact than inflammation pathway on etiology of infertile men with varicocele. Andrologia 2019;51.

66. Ghazavi-Khorasgani N, Janghorban-Laricheh E, Tavalaee M, Zohrabi D, Abbasi H, Nasr-Esfahani MH. Evaluation of post-acrosomal sheath WW domain binding protein expression in spermatozoa from infertile men with varicocele. Tehran University Medical Journal 2017;75:417-423.

67. Gill K, Jakubik J, Kups M, Rosiak-Gill A, Kurzawa R, Kurpisz M, Fraczek M, et al. The impact of sedentary work on sperm nuclear DNA integrity. Folia Histochemica et Cytobiologica 2019;57:15-22.

68. Gill K, Jakubik-Uljasz J, Rosiak-Gill A, Grabowska M, Matuszewski M, Piasecka M. Male aging as a causative factor of detrimental changes in human conventional semen parameters and sperm DNA integrity. Aging Male 2020;23:1321-1332.

69. Gill K, Kups M, Harasny P, Machalowski T, Grabowska M, Lukaszuk M, Matuszewski M, et al. The negative impact of varicocele on basic semen parameters, sperm nuclear dna dispersion and oxidation-reduction potential in semen. International Journal of Environmental Research and Public Health 2021;18.

70. Giwercman A, Rylander L, Rignell-Hydbom A, Jönsson BAG, Pedersen HS, Ludwicki JK, Lesovoy V, et al. Androgen receptor gene CAG repeat length as a modifier of the association between persistent organohalogen pollutant exposure markers and semen characteristics. Pharmacogenetics and Genomics 2007;17:391-401.

71. Gosálvez J, González-Martínez M, López-Fernández C, Fernández JL, Sánchez-Martín P. Shorter abstinence decreases sperm deoxyribonucleic acid fragmentation in ejaculate. Fertility and Sterility 2011;96:1083-1086.

72. Grosen A, Bellaguarda E, Nersting J, Hvas CL, Liljeqvist-Soltic I, Stein A, Christensen LA, et al. Low-dose Methotrexate Therapy Does Not Affect Semen Parameters and Sperm DNA. Inflammatory bowel diseases 2021.

73. Grosen A, Bungum M, Christensen LA, Cordelli E, Larsen OH, Leter G, Julsgaard M, et al. Semen Quality and Sperm DNA Integrity in Patients with Severe Active Inflammatory Bowel Disease and Effects of Tumour Necrosis Factor-alpha Inhibitors. Journal of Crohn's and Colitis 2019;13:564-571.

74. Grosen A, Bungum M, Hvas CL, Julsgaard M, Cordelli E, Kelsen J. Vedolizumab Does Not Impair Sperm DNA Integrity in Men With Inflammatory Bowel Disease. Gastroenterology 2019;156:2342-2344.

75. Grosen A, Nersting J, Bungum M, Christensen LA, Schmiegelow K, Spanò M, Julsgaard M, et al. Sperm DNA integrity is unaffected by thiopurine treatment in men with inflammatory bowel disease. Journal of Crohn's and Colitis 2019;13:3-11.

76. Guo LY, Zhou H, Liu M, Li Q, Sun XF. Male age is more critical to sperm DNA integrity than routine semen parameters in Chinese infertile males. Andrologia 2020;52.

77. Håkonsen LB, Spano M, Bonde JP, Olsen J, Thulstrup AM, Ernst E, Ramlau-Hansen CH. Exposures that may affect sperm DNA integrity: Two decades of follow-up in a pregnancy cohort. Reproductive Toxicology 2012;33:316-321.

78. Hammadeh ME, Hamad MF, Montenarh M, Fischer-Hammadeh C. Protamine contents and P1/P2 ratio in human spermatozoa from smokers and non-smokers. Human Reproduction 2010;25:2708-2720.

79. Hammiche F, Laven JSE, Boxmeer JC, Dohle GR, Steegers EAP, Steegers-Theunissen RPM. Sperm quality decline among men below 60 years of age undergoing IVF or ICSI treatment. Journal of Andrology 2011;32:70-76.

80. Hansen ML, Thulstrup AM, Bonde JP, Olsen J, Håkonsen LB, Ramlau-Hansen CH. Does last week's alcohol intake affect semen quality or reproductive hormones? A cross-sectional study among healthy young Danish men. Reproductive Toxicology 2012;34:457-462.

81. Henkel R, Kierspel E, Hajimohammad M, Stalf T, Hoogendijk C, Mehnert C, Menkveld R, et al. DNA fragmentation of spermatozoa and assisted reproduction technology. Reproductive BioMedicine Online 2003;7:477-484.

82. Homa ST, Vassiliou AM, Stone J, Killeen AP, Dawkins A, Xie J, Gould F, et al. A comparison between two assays for measuring seminal oxidative stress and their relationship with sperm DNA fragmentation and semen parameters. Genes 2019;10.

83. Horta F, Madariaga M, García A, Hartel S, Smith R. Association of age with sperm DNA fragmentation. Revista Medica de Chile 2011;139:306-312.

84. Huang LP, Lee CC, Hsu PC, Shih TS. The association between semen quality in workers and the concentration of di(2-ethylhexyl) phthalate in polyvinyl chloride pellet plant air. Fertility and Sterility 2011;96:90-94.

85. Humaidan P, Haahr T, Povlsen BB, Kofod L, Laursen RJ, Alsbjerg B, Elbaek HO, et al. The combined effect of lifestyle intervention and antioxidant therapy on sperm DNA fragmentation and seminal oxidative stress in IVF patients: a pilot study. International braz j urol : official journal of the Brazilian Society of Urology 2021;47.

86. Iommiello VM, Albani E, Di Rosa A, Marras A, Menduni F, Morreale G, Levi SL, et al. Ejaculate oxidative stress is related with sperm DNA fragmentation and round cells. International Journal of Endocrinology 2015;2015.

87. Janghorban-Laricheh E, Ghazavi-Khorasgani N, Tavalaee M, Zohrabi D, Abbasi H, Nasr- Esfahani MH. An association between sperm PLCζ levels and varicocele? Journal of Assisted Reproduction and Genetics 2016;33:1649-1655.

88. Jeng HA, Pan CH, Chao MR, Lin WY. Sperm DNA oxidative damage and DNA adducts. Mutation Research - Genetic Toxicology and Environmental Mutagenesis 2015;794:75-82.

89. Jeremias JT, Belardin LB, Okada FK, Antoniassi MP, Fraietta R, Bertolla RP, Intasqui P. Oxidative origin of sperm DNA fragmentation in the adult varicocele. International braz j urol : official journal of the Brazilian Society of Urology 2021;47:275-283.

90. Ji G, Xia Y, Gu A, Shi X, Long Y, Song L, Wang S, et al. Effects of non-occupational environmental exposure to pyrethroids on semen quality and sperm DNA integrity in Chinese men. Reproductive Toxicology 2011;31:171-176.

91. Ji G, Yan L, Wu S, Liu J, Wang L, Zhang S, Shi L, et al. Bulky DNA adducts in human sperm associated with semen parameters and sperm DNA fragmentation in infertile men: A cross-sectional study. Environmental Health: A Global Access Science Source 2013;12.

92. Jurewicz J, Radwan M, Sobala W, Radwan P, Bochenek M, Hanke W. Dietary Patterns and Their Relationship With Semen Quality. American journal of men's health 2018;12:575-583.

93. Kabukçu C, Çil N, Çabuş Ü, Alataş E. Effect of ejaculatory abstinence period on sperm DNA fragmentation and pregnancy outcome of intrauterine insemination cycles: A prospective randomized study. Archives of gynecology and obstetrics 2021;303:269‐278.

94. Karimi J, Goodarzi MT, Tavilani H, Khodadadi I, Amiri I. Increased receptor for advanced glycation end products in spermatozoa of diabetic men and its association with sperm nuclear DNA fragmentation. Andrologia 2012;44:280-286.

95. Kaspersen MD, Bungum M, Fedder J, Bonde J, Larsen PB, J Ingerslev H, Höllsberg P. No increased sperm DNA fragmentation index in semen containing human papillomavirus or herpesvirus. Andrology 2013;1:361-364.

96. Kavoussi P, Abdullah N, Gilkey M, Hunn C, MacHen G, Chen SH, Kavoussi K, et al. The impact of ipsilateral testicular atrophy on semen quality and sperm DNA fragmentation response to varicocele repair. Asian journal of andrology 2021;23:146‐149.

97. Kiwitt-Cárdenas J, Adoamnei E, Arense-Gonzalo JJ, Sarabia-Cos L, Vela-Soria F, Fernández MF, Gosálvez J, et al. Associations between urinary concentrations of bisphenol A and sperm DNA fragmentation in young men. Environmental Research 2021;199.

98. Krüger T, Spanò M, Long M, Eleuteri P, Rescia M, Hjelmborg PS, Manicardi GC, et al. Xenobiotic activity in serum and sperm chromatin integrity in European and Inuit populations. Molecular Reproduction and Development 2008;75:669-680.

99. Kumar D, Salian SR, Kalthur G, Uppangala S, Kumari S, Challapalli S, Chandraguthi SG, et al. Semen Abnormalities, Sperm DNA Damage and Global Hypermethylation in Health Workers Occupationally Exposed to Ionizing Radiation. PLoS ONE 2013;8.

100. Kumar SB, Chawla B, Bisht S, Yadav RK, Dada R. Tobacco Use Increases Oxidative DNA Damage in Sperm - Possible Etiology of Childhood Cancer. Asian Pacific journal of cancer prevention : APJCP 2015;16:6967-6972.

101. La Vignera S, Condorelli R, Vicari E, D'Agata R, Calogero AE. Effects of varicocelectomy on sperm DNA fragmentation, mitochondrial function, chromatin condensation, and apoptosis. Journal of Andrology 2012;33:389-396.

102. Laqqan MM, Yassin MM. Potential effect of tobacco cigarettes smoking on global DNA methylation status and protamines transcripts in human spermatozoa. Middle East Fertility Society Journal 2021;26.

103. Lara-Cerrillo S, Gual-Frau J, Benet J, Abad C, Prats J, Amengual MJ, García-Peiró A. Microsurgical varicocelectomy effect on sperm telomere length, DNA fragmentation and seminal parameters. Human Fertility 2020.

104. Le MT, Nguyen DN, Le DD, Tran NQT. Impact of body mass index and metabolic syndrome on sperm DNA fragmentation in males from infertile couples: A cross-sectional study from Vietnam. Metabolism Open 2020;7.

105. Le MT, Thi Tran NQ, Nguyen ND, Vu Nguyen QH. The prevalence and components of metabolic syndrome in men from infertile couples and its relation on semen analysis. Diabetes, Metabolic Syndrome and Obesity: Targets and Therapy 2021;14:1453-1463.

106. Lenters V, Portengen L, Smit LAM, Jönsson BAG, Giwercman A, Rylander L, Lindh CH, et al. Phthalates, perfluoroalkyl acids, metals and organochlorines and reproductive function: A multipollutant assessment in Greenlandic, Polish and Ukrainian men. Occupational and Environmental Medicine 2015;72:385-393.

107. Li F, Yamaguchi K, Okada K, Matsushita K, Ando M, Chiba K, Yue H, et al. Significant improvement of sperm DNA quality after microsurgical repair of varicocele. Systems Biology in Reproductive Medicine 2012;58:274-277.

108. Liu KS, Mao XD, Pan F, An RF. Effect and mechanisms of reproductive tract infection on oxidative stress parameters, sperm DNA fragmentation, and semen quality in infertile males. Reproductive Biology and Endocrinology 2021;19.

109. Liu KS, Mao XD, Pan F, Chen YJ. Application of leukocyte subsets and sperm DNA fragment rate in infertile men with asymptomatic infection of genital tract. Annals of palliative medicine 2021;10:1021.

110. Long M, Stronati A, Bizzaro D, Krüger T, Manicardi GC, Hjelmborg PS, Spanò M, et al. Relation between serum xenobiotic-induced receptor activities and sperm DNA damage and sperm apoptotic markers in European and Inuit populations. Reproduction 2007;133:517-530.

111. Lu JC, Jing J, Chen L, Ge YF, Feng RX, Liang YJ, Yao B. Analysis of human sperm DNA fragmentation index (DFI) related factors: A report of 1010 subfertile men in China. Reproductive Biology and Endocrinology 2018;16.

112. Lu R, Chen X, Yu W, Jiang F, Zhou X, Xu Y, Wang F. Analysis of age-associated alternation of SCSA sperm DNA fragmentation index and semen characteristics of 1790 subfertile males in China. Journal of Clinical Laboratory Analysis 2020;34.

113. Lu X, Huang Y, Zhang H, Zhao J. Effect of diabetes mellitus on the quality and cytokine content of human semen. Journal of Reproductive Immunology 2017;123:1-2.

114. Ma XP, Gao XQ. The effect of Ureaplasma urealyticum on the level of P34H expression, the activity of hyaluronidase, and DNA fragmentation in human spermatozoa. American Journal of Reproductive Immunology 2017;77.

115. Mahfouz R, Sharma R, Thiyagarajan A, Kale V, Gupta S, Sabanegh E, Agarwal A. Semen characteristics and sperm DNA fragmentation in infertile men with low and high levels of seminal reactive oxygen species. Fertility and Sterility 2010;94:2141-2146.

116. Mahran AM, Mosad E, Abdel-Raheem MA, Ahmed EH, Abdel Motaleb AA, Hofny ER. The correlation between mammalian target of rapamycin (mTOR) gene expression and sperm DNA damage among infertile patients with and without varicocele. Andrologia 2019;51.

117. Malm G, Haugen T, Rylander L, Giwercman A. Seasonal fluctuation in the secretion of the antioxidant melatonin is not associated with alterations in sperm DNA damage. Asian Journal of Andrology 2017;19:52-56.

118. Manna C, Barbagallo F, Manzo R, Rahman A, Francomano D, Calogero AE. Sperm parameters before and after swim-up of a second ejaculate after a short period of abstinence. Journal of Clinical Medicine 2020;9.

119. Marchlewska K, Filipiak E, Walczak-Jedrzejowska R, Oszukowska E, Sobkiewicz S, Wojt M, Chmiel J, et al. Sperm DNA Fragmentation Index and Hyaluronan Binding Ability in Men from Infertile Couples and Men with Testicular Germ Cell Tumor. BioMed Research International 2016;2016.

120. Martínez E, Bezazián C, Bezazián A, Lindl K, Peliquero A, Cattaneo A, Gnocchi D, et al. Sperm DNA fragmentation and male age: results of in vitro fertilization treatments. JBRA Assist Reprod 2021;25:533-539.

121. Mayorga-Torres BJM, Camargo M, Agarwal A, du Plessis SS, Cadavid P, Cardona Maya WD. Influence of ejaculation frequency on seminal parameters. Reproductive Biology and Endocrinology 2015;13.

122. Mayorga-Torres JM, Agarwal A, Roychoudhury S, Cadavid A, Cardona-Maya WD. Can a short term of repeated ejaculations affect seminal parameters? Journal of Reproduction and Infertility 2016;17:177-183.

123. McDowell S, Harrison K, Kroon B, Ford E, Yazdani A. Sperm DNA fragmentation in men with malignancy. Fertility and Sterility 2013;99:1862-1866.

124. Meseguer M, Santiso R, Garrido N, Fernandez JL. The effect of cancer on sperm DNA fragmentation as measured by the sperm chromatin dispersion test. Fertility and Sterility 2008;90:225-227.

125. Mohammed EE, Mosad E, Zahran AM, Hameed DA, Taha EA, Mohamed MA. Acridine Orange and Flow Cytometry: Which Is Better to Measure the Effect of Varicocele on Sperm DNA Integrity? Adv Urol 2015;2015:814150.

126. Moskovtsev SI, Lecker I, Mullen JBM, Jarvi K, Willis J, White J, Lo KC. Cause-specific treatment in patients with high sperm DNA damage resulted in significant DNA improvement. Systems Biology in Reproductive Medicine 2009;55:109-115.

127. Moskovtsev SI, Willis J, White J, Mullen JBM. Sperm DNA Damage: Correlation to Severity of Semen Abnormalities. Urology 2009;74:789-793.

128. Moustafa M, Sharma RK, Thornton J, Mascha E, Abdel-Hafez MA, Thomas Jr AJ, Agarwal A. Relationship between ROS production, apoptosis and DNA denaturation in spermatozoa from patients examined for infertility. Human Reproduction 2004;19:129-138.

129. Nazmara Z, Shirinbayan P, reza Asgari H, Ahadi R, Asgari F, Maki CB, Fattahi F, et al. The epigenetic alterations of human sperm cells caused by heroin use disorder. Andrologia 2021;53.

130. Nguyen TT, Trieu TS, Tran TO, Luong TLA. Evaluation of sperm DNA fragmentation index, Zinc concentration and seminal parameters from infertile men with varicocele. Andrologia 2019;51.

131. Ni K, Steger K, Yang H, Wang H, Hu K, Zhang T, Chen B. A comprehensive investigation of sperm DNA damage and oxidative stress injury in infertile patients with subclinical, normozoospermic, and astheno/oligozoospermic clinical varicocoele. Andrology 2016;4:816-824.

132. Nijs M, De Jonge C, Cox A, Janssen M, Bosmans E, Ombelet W. Correlation between male age, WHO sperm parameters, DNA fragmentation, chromatin packaging and outcome in assisted reproduction technology. Andrologia 2011;43:174-179.

133. Oliveira JBA, Petersen CG, Mauri AL, Vagnini LD, Baruff RLR, Franco Jr JG. The effects of age on sperm quality: An evaluation of 1,500 semen samples. Jornal Brasileiro de Reproducao Assistida 2014;18:34-41.

134. Oliveira JBA, Petersen CG, Mauri AL, Vagnini LD, Renzi A, Petersen B, Mattila M, et al. Association between body mass index and sperm quality and sperm DNA integrity. A large population study. Andrologia 2018;50.

135. Osadchuk LV, Erkovich AA, Tataru DA, Markova EV, Svetlakov AV. [Level of DNA fragmentation in human sperm cells in varicocele and prostatitis]. Urologii{combining double inverted breve}a (Moscow, Russia : 1999) 2014:37-43.

136. Pant N, Shukla M, Upadhyay AD, Chaturvedi PK, Saxena DK, Gupta YK. Association between environmental exposure to p, p'-DDE and lindane and semen quality. Environmental science and pollution research international 2014;21:11009-11016.

137. Pearce KL, Hill A, Tremellen KP. Obesity related metabolic endotoxemia is associated with oxidative stress and impaired sperm DNA integrity. Basic Clin Androl 2019;29:6.

138. Pelliccione F, Verratti V, D'Angeli A, Micillo A, Doria C, Pezzella A, Iacutone G, et al. Physical exercise at high altitude is associated with a testicular dysfunction leading to reduced sperm concentration but healthy sperm quality. Fertility and Sterility 2011;96:28-33.

139. Petersen CG, Mauri AL, Vagnini LD, Renzi A, Petersen B, Mattila M, Comar V, et al. The effects of male age on sperm DNA damage: An evaluation 2,178 semen samples. Jornal Brasileiro de Reproducao Assistida 2018;22:323-330.

140. Pons I, Cercas R, Villas C, Braña C, Fernández-Shaw S. One abstinence day decreases sperm DNA fragmentation in 90 % of selected patients. Journal of Assisted Reproduction and Genetics 2013;30:1211-1218.

141. Rago R, Salacone P, Caponecchia L, Sebastianelli A, Marcucci I, Calogero AE, Condorelli R, et al. The semen quality of the mobile phone users. Journal of Endocrinological Investigation 2013;36:970-974.

142. Ranganathan P, Rao KA, Thalaivarasai Balasundaram S. Deterioration of semen quality and sperm-DNA integrity as influenced by cigarette smoking in fertile and infertile human male smokers—A prospective study. Journal of Cellular Biochemistry 2019;120:11784-11793.

143. Ribeiro TM, Bertolla RP, Spaine DM, Fraietta R, Ortiz V, Cedenho AP. Sperm nuclear apoptotic DNA fragmentation in men with testicular cancer. Fertility and Sterility 2008;90:1782-1786.

144. Romerius P, Ståhl O, Moëll C, Relander T, Cavallin-Ståhl E, Gustafsson H, Thapper KL, et al. Sperm DNA integrity in men treated for childhood cancer. Clinical Cancer Research 2010;16:3843-3850.

145. Rosiak-Gill A, Gill K, Jakubik J, Fraczek M, Patorski L, Gaczarzewicz D, Kurzawa R, et al. Age-related changes in human sperm DNA integrity. Aging 2019;11:5399-5411.

146. Rubes J, Rybar R, Prinosilova P, Veznik Z, Chvatalova I, Solansky I, Sram RJ. Genetic polymorphisms influence the susceptibility of men to sperm DNA damage associated with exposure to air pollution. Mutation Research - Fundamental and Molecular Mechanisms of Mutagenesis 2010;683:9-15.

147. Rubes J, Sipek J, Kopecka V, Musilova P, Vozdova M, Prinosilova P, Topinka J, et al. The effects of age on DNA fragmentation, the condensation of chromatin and conventional semen parameters in healthy nonsmoking men exposed to traffic air pollution. Health Science Reports 2021;4.

148. Safarinejad MR. Sperm DNA Damage and Semen Quality Impairment After Treatment With Selective Serotonin Reuptake Inhibitors Detected Using Semen Analysis and Sperm Chromatin Structure Assay. Journal of Urology 2008;180:2124-2128.

149. Safarinejad MR. Sperm chromatin structure assay analysis of Iranian mustard gas casualties: A long-term outlook. Current Urology 2010;4:71-80.

150. Said TM, Tellez S, Evenson DP, Del Valle AP. Assessment of sperm quality, DNA integrity and cryopreservation protocols in men diagnosed with testicular and systemic malignancies. Andrologia 2009;41:377-382.

151. Saleh RA, Agarwal A, Sharma RK, Said TM, Sikka SC, Thomas Jr AJ. Evaluation of nuclear DNA damage in spermatozoa from infertile men with varicocele. Fertility and Sterility 2003;80:1431-1436.

152. Savasi V, Oneta M, Laoreti A, Parisi F, Parrilla B, Duca P, Cetin I. Effects of Antiretroviral Therapy on Sperm DNA Integrity of HIV-1-Infected Men. American journal of men's health 2018;12:1835-1842.

153. Sepaniak S, Forges T, Gerard H, Foliguet B, Bene MC, Monnier-Barbarino P. The influence of cigarette smoking on human sperm quality and DNA fragmentation. Toxicology 2006;223:54-60.

154. Smit M, Van Casteren NJ, Wildhagen MF, Romijn JC, Dohle GR. Sperm DNA integrity in cancer patients before and after cytotoxic treatment. Human Reproduction 2010;25:1877-1883.

155. Smit M, Wissenburg OG, Romijn JC, Dohle GR. Increased Sperm DNA Fragmentation in Patients With Vasectomy Reversal Has No Prognostic Value for Pregnancy Rate. Journal of Urology 2010;183:662-665.

156. Smith G R, Kaune G H, Parodi Ch D, Madariaga A M, Morales D I, Ríos S R, Castro G A. Extent of sperm DNA damage in spermatozoa from men examined for infertility. Relationship with oxidative stress. Revista Medica de Chile 2007;135:279-286.

157. Smith R, Kaune H, Parodi D, Madariaga M, Rios R, Morales I, Castro A. Increased sperm DNA damage in patients with varicocele: Relationship with seminal oxidative stress. Human Reproduction 2006;21:986-993.

158. Spanò M, Toft G, Hagmar L, Eleuteri P, Rescia M, Rignell-Hydbom A, Tyrkiel E, et al. Exposure to PCB and p,p′-DDE in European and Inuit populations: Impact on human sperm chromatin integrity. Human Reproduction 2005;20:3488-3499.

159. Specht IO, Hougaard KS, Spanò M, Bizzaro D, Manicardi GC, Lindh CH, Toft G, et al. Sperm DNA integrity in relation to exposure to environmental perfluoroalkyl substances - A study of spouses of pregnant women in three geographical regions. Reproductive Toxicology 2012;33:577-583.

160. Ståhl O, Eberhard J, Cavallin-Ståhl E, Jepson K, Friberg B, Tingsmark C, Spanò M, et al. Sperm DNA integrity in cancer patients: The effect of disease and treatment. International Journal of Andrology 2009;32:695-703.

161. Ståhl O, Eberhard J, Jepson K, Spano M, Cwikiel M, Cavallin-Ståhl E, Giwercman A. The Impact of Testicular Carcinoma and its Treatment on Sperm DNA Integrity. Cancer 2004;100:1137-1144.

162. Ståhl O, Eberhard J, Jepson K, Spano M, Cwikiel M, Cavallin-Ståhl E, Giwercman A. Sperm DNA integrity in testicular cancer patients. Human Reproduction 2006;21:3199-3205.

163. Stronati A, Manicardi GC, Cecati M, Bordicchia M, Ferrante L, Spanò M, Toft G, et al. Relationships between sperm DNA fragmentation, sperm apoptotic markers and serum levels of CB-153 and p,p'-DDE in European and Inuit populations. Reproduction 2006;132:949-958.

164. Taha EA, Ez-Aldin AM, Sayed SK, Ghandour NM, Mostafa T. Effect of smoking on sperm vitality, DNA integrity, seminal oxidative stress, zinc in fertile men. Urology 2012;80:822-825.

165. Taha EA, Ezz-Aldin AM, Sayed SK, Ghandour NM, Mostafa T. Smoking influence on sperm vitality, DNA fragmentation, reactive oxygen species and zinc in oligoasthenoteratozoospermic men with varicocele. Andrologia 2014;46:687-691.

166. Taha EA, Mekky MA, Gaber HD, Abdel-Gaber RM, Zahran AM, Abd Allah ESH, Mohamed AQ, et al. Impact of chronic hepatitis B virus infection on semen parameters of fertile men. Future Virology 2019;14:515-522.

167. Taha EA, Sayed SK, Gaber HD, Abdel Hafez HK, Ghandour N, Zahran A, Mostafa T. Does being overweight affect seminal variables in fertile men? Reproductive BioMedicine Online 2016;33:703-708.

168. Tahamtan S, Tavalaee M, Izadi T, Barikrow N, Zakeri Z, Lockshin RA, Abbasi H, et al. Reduced sperm telomere length in individuals with varicocele is associated with reduced genomic integrity. Scientific reports 2019;9:4336.

169. Talebi AR, Moein MR, Tabibnejad N, Ghasemzadeh J. Effect of varicocele on chromatin condensation and DNA integrity of ejaculated spermatozoa using cytochemical tests. Andrologia 2008;40:245-251.

170. Tanaka T, Kobori Y, Terai K, Inoue Y, Osaka A, Yoshikawa N, Shimomura Y, et al. Seminal oxidation–reduction potential and sperm DNA fragmentation index increase among infertile men with varicocele. Human Fertility 2020.

171. Tangal S, Taşçı Y, Pabuçcu EG, Çağlar GS, Haliloğlu AH, Yararbaş K. DNA fragmentation index and human papilloma virus in males with previous assisted reproductive technology failures. Turk J Urol 2019;45:12-16.

172. Tartibian B, Maleki BH. Correlation between seminal oxidative stress biomarkers and antioxidants with sperm DNA damage in elite athletes and recreationally active men. Clinical Journal of Sport Medicine 2012;22:132-139.

173. Vagnini L, Baruffi RLR, Mauri AL, Petersen CG, Massaro FC, Pontes A, Oliveira JBA, et al. The effects of male age on sperm DNA damage in an infertile population. Reproductive BioMedicine Online 2007;15:514-519.

174. van Brakel J, Dinkelman-Smit M, de Muinck Keizer-Schrama SMPF, Hazebroek FWJ, Dohle GR. Sperm DNA damage measured by sperm chromatin structure assay in men with a history of undescended testes. Andrology 2017;5:838-843.

175. Vargas-Baquero E, Johnston S, Sánchez-Ramos A, Arévalo-Martín A, Wilson R, Gosálvez J. The incidence and etiology of sperm DNA fragmentation in the ejaculates of males with spinal cord injuries. Spinal Cord 2020;58:803-810.

176. Vaughan DA, Tirado E, Garcia D, Datta V, Sakkas D. DNA fragmentation of sperm: A radical examination of the contribution of oxidative stress and age in 16 945 semen samples. Human Reproduction 2020;35:2188-2196.

177. Vellani E, Colasante A, Mamazza L, Minasi MG, Greco E, Bevilacqua A. Association of state and trait anxiety to semen quality of in vitro fertilization patients: A controlled study. Fertility and Sterility 2013;99:1565-1572.e1562.

178. Vinnakota C, Cree L, Peek J, Morbeck DE. Incidence of high sperm DNA fragmentation in a targeted population of subfertile men. Systems Biology in Reproductive Medicine 2019;65:451-457.

179. Vivas-Acevedo G, Lozano-Hernández R, Camejo MI. Varicocele decreases epididymal neutral α-glucosidase and is associated with alteration of nuclear DNA and plasma membrane in spermatozoa. BJU International 2014;113:642-649.

180. Vujkovic M, De Vries JH, Dohle GR, Bonsel GJ, Lindemans J, MacKlon NS, Van Der Spek PJ, et al. Associations between dietary patterns and semen quality in men undergoing IVF/ICSI treatment. Human Reproduction 2009;24:1304-1312.

181. Wang X, Chen Q, Zou P, Liu T, Mo M, Yang H, Zhou N, et al. Sleep duration is associated with sperm chromatin integrity among young men in Chongqing, China. Journal of Sleep Research 2018;27.

182. Wang Y, Zhang W, Li D. High ligation of varicocele improves sperm DNA integrity in patients with asthenospermia. Journal of Central South University (Medical Sciences) 2012;37:1228-1232.

183. Wijesekara GUS, Fernando DMS, Wijeratne S. The effects of Pb on sperm parameters and sperm DNA fragmentation of men investigated for infertility. Journal of Basic and Clinical Physiology and Pharmacology 2020;31.

184. Winkle T, Rosenbusch B, Gagsteiger F, Paiss T, Zoller N. The correlation between male age, sperm quality and sperm DNA fragmentation in 320 men attending a fertility center. Journal of Assisted Reproduction and Genetics 2009;26:41-46.

185. Wyrobek AJ, Eskenazi B, Young S, Arnheim N, Tiemann-Boege I, Jabs EW, Glaser RL, et al. Advancing age has differential effects on DNA damage, chromatin integrity, gene mutations, and aneuploidies in sperm. Proceedings of the National Academy of Sciences of the United States of America 2006;103:9601-9606.

186. Yang Q, Zhao F, Hu L, Bai R, Zhang N, Yao G, Sun Y. Effect of paternal overweight or obesity on IVF treatment outcomes and the possible mechanisms involved. Scientific reports 2016;6:29787.

187. Zeqiraj A, Gashi Z, Elezaj S, Aliu H, Bexheti S, Hajrulai-Musliu Z, Shabani A. Sperm DNA fragmentation, age and male infertility. International Medical Journal 2019;26:370-372.

188. Zeyad A, Hamad MF, Hammadeh ME. The effects of bacterial infection on human sperm nuclear protamine P1/P2 ratio and DNA integrity. Andrologia 2018;50.

189. Zhang F, Li J, Liang Z, Wu J, Li L, Chen C, Jin F, et al. Sperm DNA fragmentation and male fertility: a retrospective study of 5114 men attending a reproductive center. Journal of Assisted Reproduction and Genetics 2021;38:1133-1141.

190. Zhu G, Zhang Y, Dong J, Liu Y, Zhao F, Li T, Shi Z, et al. Association between body mass index and male sperm apoptosis and apoptosis-related factors. Diabetes, Metabolic Syndrome and Obesity: Targets and Therapy 2021;14:1043-1051.
