# Supplementary material for: Lifestyle-, environmental-, and additional health factors associated with an increased sperm DNA fragmentation: a systematic review and meta-analysis
Source: Reprod Biol Endocrinol. 2023 Jan 18;21:5. doi: 10.1186/s12958-023-01054-0 (PMC9847125; doi:10.1186/s12958-023-01054-0)
Supplement: Supplementary file 1 — Additional file 1: Supplementary Appendix 1. Risk of bias assessment methodology. Supplementary Table 1. PRISMA 2020 checklist. Supplementary Table 2. Basic characteristics of the included article. Supplementary Table 3. Eligibility criteria in each included article. Supplementary Table 4. Risk factor and population definitions in each included article. Supplementary Table 5. Risk of bias assessment using the QUIPS tools. Supplementary Table 6. Articles also looking at pregnancy or birth as an outcome. Supplementary Figure 1. Comparison of patients’ sperm DNA fragmentation values with and without varicocele subdivided based on sperm DNA fragmentation assays used (continuous data). Supplementary Figure 2. Comparison of patients’ sperm DNA fragmentation values with and without varicocele subdivided based on different cut-off values. Supplementary Figure 3. Supplementary Figure 4. Comparison of patients’ sperm DNA fragmentation values with and without varicocele subdivided based on the fertility status of patients (continuous data). Supplementary Figure 5. Comparison of patients’ sperm DNA fragmentation values with impaired and normal glucose tolerance (continuous data). Supplementary Figure 6. Comparison of patients’ sperm DNA fragmentation values with and without testicular tumors subdivided based on sperm DNA fragmentation assays used (continuous data). Supplementary Figure 7. Comparison of patients’ sperm DNA fragmentation values with and without Hodgkin-lymphoma (HL) (continuous data). Supplementary Figure 8. Comparison of patients’ sperm DNA fragmentation values with and without non-Hodgkin lymphoma (NHL) (continuous data). Supplementary Figure 9. Comparison of patients’ sperm DNA fragmentation values with and without lymphomas (continuous data). Supplementary Figure 10. Comparison of patients’ sperm DNA fragmentation values with and without leukemia (continuous data). Supplementary Figure 11. Comparison of patients’ sperm DNA fragmentation values with and withou [file 12958_2023_1054_MOESM1_ESM.zip › ESM1/Supplementary Figure 46.pdf]

Mean differences

				Group 1			Group 2					
**Studies**	**Comparison**	**Population**	**Assay**	**N**	**Mean**	**SD[1]**	**N**	**Mean**	**SD[1]**	**MD[2]**	**95%-CI[3]**	**Visualization**
**Borges 2019**	abstinence>4days - abstinence<=4days	fertility clinic	SCD	20	19.20	0.80	463	16.80	0.70	**2.4**	**(2, 2.8)**	
**van Brakel 2017**	acquired undescended testes - no undescended testes	fertility clinic + fertile controls	SCSA	49	19.80	11.20	22	15.70	5.10	**4.1**	**(0.3, 7.9)**	
**Stahl 2004**	adjuvant chemotherapy - no tumor	TGCC patients + general population	SCSA	29	13.80	5.80	278	16.60	8.40	**-2.8**	**(-5.1, -0.5)**	
**Stahl 2004**		TGCC patients 6 months after therapy + general population	SCSA	12	10.20	3.50	278	16.60	8.40	**-6.4**	**(-8.6, -4.2)**	
**Stahl 2004**		TGCC pts 12-24 months after therapy + general popul controls	SCSA	20	16.70	7.80	278	16.60	8.40	**0.1**	**(-3.5, 3.7)**	
**Stahl 2004**		TGCC pts 30-60 months after therapy + general popul controls	SCSA	12	16.20	6.50	278	16.60	8.40	**-0.4**	**(-4.2, 3.4)**	
**Stahl 2006**		TGCC patients 6 months after therapy + general population	TUNEL	12	10.70	6.50	278	16.60	8.40	**-5.9**	**(-9.7, -2.1)**	
**Stahl 2006**		TGCC pts 12-24 months after therapy + general popul controls	TUNEL	22	13.30	6.80	24	13.90	7.30	**-0.6**	**(-4.7, 3.5)**	
**Stahl 2006**		TGCC pts 36-60 months after therapy + general popul controls	TUNEL	19	12.60	4.30	24	13.90	7.30	**-1.3**	**(-4.8, 2.2)**	

[1] Standar deviation [2] Mean difference [3] 95% Confidence Interval

				Group 1			Group 2					
Studies	Comparison	Population	Assay	N	Mean	SD[1]	N	Mean	SD[1]	MD[2]	95%-CI[3]	Visualization
**Stahl 2006**		TGCC patients 6 months after therapy + general population	SCSA	15	13.40	6.50	278	21.40	10.60	**-8.0**	**(-11.5, -4.5)**	
**Stahl 2006**		TGCC pts 12-24 months after therapy + general popul controls	SCSA	24	19.80	9.50	278	21.40	10.60	**-1.6**	**(-5.6, 2.4)**	
**Stahl 2006**		TGCC pts 36-60 months after therapy + general popul controls	SCSA	21	15.30	6.60	278	21.40	10.60	**-6.1**	**(-9.2, -3)**	
**Winkle 2008**	age=>30 - age<30	fertility clinic (but normozoospermic)	SCSA	63	9.56	11.49	6	7.92	3.93	**1.6**	**(-2.6, 5.9)**	
**Winkle 2008**	age=>40 - age<40	fertility clinic (but normozoospermic)	SCSA	13	9.99	11.86	56	9.29	10.94	**0.7**	**(-6.4, 7.8)**	
**Winkle 2008**	age>35 - age=<35	fertility clinic (but normozoospermic)	SCSA	36	11.32	14.42	33	7.34	5.36	**4.0**	**(-1.1, 9)**	
**Tartibian 2012**	athlete - recreationally active	athletes + recreationally active)	TUNEL	56	7.20	3.60	52	2.30	1.30	**4.9**	**(3.9, 5.9)**	
**Smit 2010**	BEP+radioth or radioth only - BEP only	TGCT patients	SCSA	13	21.30	8.50	17	16.10	8.80	**5.2**	**(-1, 11.4)**	
**Andersen 2016**		fertility clinic + general controls	SCSA	34	26.00	15.00	24	19.00	8.00	**7.0**	**(1, 13)**	
**Eisenberg 2014**	BMI 25-29,9 - BMI<25	general population	SCSA	191	14.00	8.70	83	13.10	6.90	**0.9**	**(-1, 2.8)**	
**Pelliccione 2011**		sportsmen	TUNEL	4	5.20	1.50	2	7.40	3.70	**-2.2**	**(-7.5, 3.1)**	

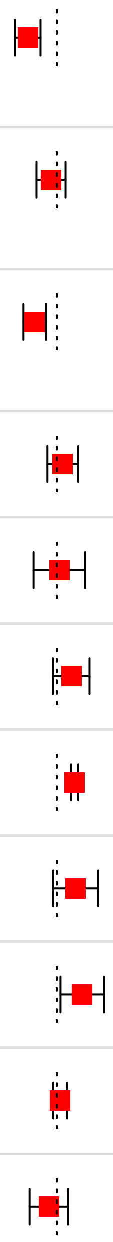

[1] Standar deviation [2] Mean difference [3] 95% Confidence Interval

Studies	Comparison	Population	Assay	Group 1 N	Group 1 Mean	Group 1 SD[1]	Group 2 N	Group 2 Mean	Group 2 SD[1]	MD[2]	95%-CI[3]	Visualization
**Andersen 2016**	BMI 30-34,9 - BMI<25	fertility clinic + general controls	SCSA	26	24.00	12.00	24	19.00	8.00	**5.0**	**(-0.6, 10.6)**	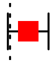
**Eisenberg 2014**		general population	SCSA	122	13.20	7.70	83	13.10	6.90	**0.1**	**(-1.9, 2.1)**	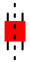
**Yang 2016**	BMI28 - 20-25 BMI	fertility clinic	SCD	54	29.00	13.00	50	19.00	11.50	**10.0**	**(5.3, 14.7)**	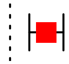
**Oliveira 2018**	BMI>=25 - BMI<=24,9	fertility clinic	TUNEL	1454	14.50	7.90	370	14.40	7.50	**0.1**	**(-0.8, 1)**	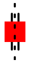
**Andersen 2016**	BMI>=25 - BMI<25	fertility clinic + general controls	SCSA	88	28.00	16.00	24	19.00	8.00	**9.0**	**(4.4, 13.6)**	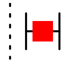
**Eisenberg 2014**		general population	SCSA	385	13.60	8.30	83	13.10	6.90	**0.5**	**(-1.2, 2.2)**	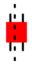
**Pearce 2019**		fertility clinic	SCD	26	17.20	6.10	3	16.10	6.80	**1.1**	**(-6.9, 9.1)**	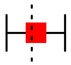
**Oliveira 2018**	BMI>=30 - BMI<30	fertility clinic	TUNEL	598	14.50	7.40	1226	14.50	8.00	**0.0**	**(-0.7, 0.7)**	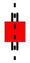
**Andersen 2016**	BMI>=35 - BMI<25	fertility clinic + general controls	SCSA	28	33.00	19.00	24	19.00	8.00	**14.0**	**(6.3, 21.7)**	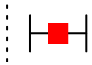
**Eisenberg 2014**		general population	SCSA	72	13.00	8.50	83	13.10	6.90	**-0.1**	**(-2.6, 2.4)**	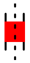
**Pearce 2019**	BMI>30 - BMI<=30	fertility clinic	SCD	14	19.30	6.10	15	15.10	5.50	**4.2**	**(0, 8.4)**	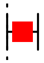
**Pelliccione 2011**	body fat >25 % - body fat 18-25 %	sportsmen	continuous	4	5.70	2.10	3	7.00	2.70	**-1.3**	**(-5, 2.4)**	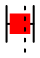

[1] Standar deviation [2] Mean difference [3] 95% Confidence Interval

Studies	Comparison	Population	Assay	Group 1 N	Group 1 Mean	Group 1 SD[1]	Group 2 N	Group 2 Mean	Group 2 SD[1]	MD[2]	95%-CI[3]	Visualization
**Romerius 2010**	childhood cancer - no cancer	general population	SCSA	99	13.00	7.50	193	11.00	7.50	**2.0**	**(0.2, 3.8)**	
**Berg 2021**	chronic prostatitis - no chronic prostatitis	patients with chronic prostatitis + general population controls	SCSA	41	25.20	14.30	22	9.90	3.80	**15.3**	**(10.6, 20)**	
**Cortés-Gutiérrez 2017**	condyloma - no condyloma	HPV negative patients	SCD	2	17.00	15.56	25	22.76	23.57	**-5.8**	**(-29.2, 17.7)**	
**van Brakel 2017**	congenital undescended testes - no undescende testes	fertility clinic + fertile controls	SCSA	50	22.70	12.80	22	15.70	5.10	**7.0**	**(2.9, 11.1)**	
**Hammiche 2011**	Dutch - Migrant	fertility clinic	SCSA	128	31.80	13.80	22	24.50	12.70	**7.3**	**(1.5, 13.1)**	
**Krüger 2008**	Europe - Greenland (lower xenobiotic exposure)	fertile	SCSA	247	28.60	15.30	53	10.70	5.90	**17.9**	**(15.4, 20.4)**	
**Long 2007**		fertile	TUNEL	208	25.90	12.30	54	5.30	3.30	**20.6**	**(18.7, 22.5)**	
**Lenters 2015**	Greenland - Poland (lower POP exposure)	fertile	TUNEL	199	2.95	45.30	197	11.64	127.90	**-8.7**	**(-27.6, 10.2)**	
**Lenters 2015**		fertile	SCSA	199	7.66	61.00	197	10.07	97.90	**-2.4**	**(-18.5, 13.7)**	
**Savasi 2018**	HAART th - no HAART	HIV pts	SCD	53	41.00	16.00	24	27.00	15.00	**14.0**	**(6.6, 21.4)**	
**Nazmara 2021**	heroin - no heroin	heroin userrs - not	SCSA	24	41.93	6.59	24	10.14	1.43	**31.8**	**(29.1, 34.5)**	

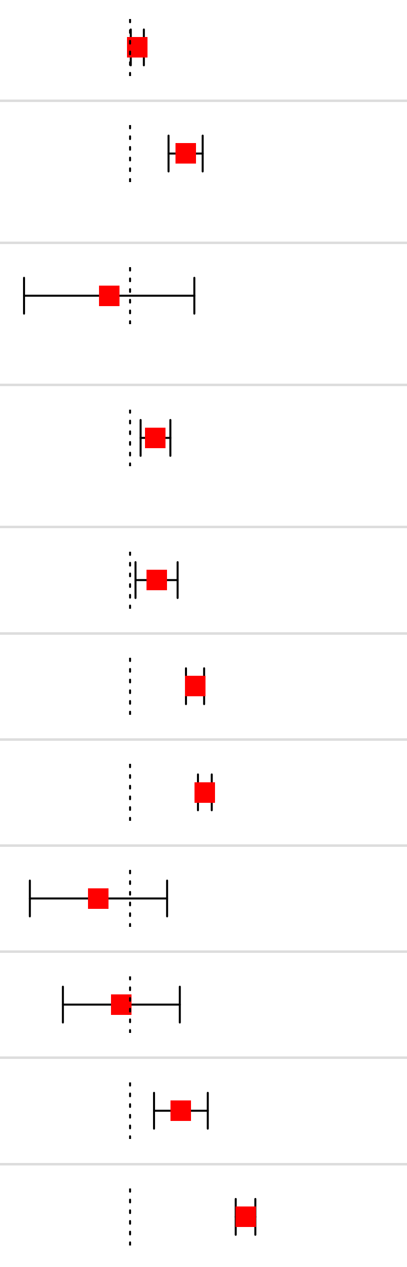

[1] Standar deviation [2] Mean difference [3] 95% Confidence Interval

				Group 1			Group 2					
Studies	Comparison	Population	Assay	N	Mean	SD[1]	N	Mean	SD[1]	MD[2]	95%-CI[3]	Visualization
**Vellani 2013**	high anxiety - low anxiety	fertility clinic	TUNEL	94	7.80	6.26	85	7.31	5.08	**0.5**	**(-1.2, 2.2)**	
**Stahl 2004**	high dose chemotherapy - no tumor	TGCC patients + general population	SCSA	16	9.40	4.70	278	16.60	8.40	**-7.2**	**(-9.7, -4.7)**	
**Stahl 2004**		TGCC pts 12-24 months after therapy + general popul controls	SCSA	9	11.80	7.70	278	16.60	8.40	**-4.8**	**(-9.9, 0.3)**	
**Stahl 2004**		TGCC pts 30-60 months after therapy + general popul controls	SCSA	9	9.90	5.70	278	16.60	8.40	**-6.7**	**(-10.6, -2.8)**	
**Stahl 2006**		TGCC patients 6 months after therapy + general population	TUNEL	3	6.50	8.10	278	16.60	8.40	**-10.1**	**(-19.3, -0.9)**	
**Stahl 2006**		TGCC pts 12-24 months after therapy + general popul controls	TUNEL	10	8.80	5.80	24	13.90	7.30	**-5.1**	**(-9.7, -0.5)**	
**Stahl 2006**		TGCC pts 36-60 months after therapy + general popul controls	TUNEL	17	11.50	6.40	24	13.90	7.30	**-2.4**	**(-6.6, 1.8)**	
**Stahl 2006**		TGCC patients 6 months after therapy + general population	SCSA	3	20.80	8.60	278	21.40	10.60	**-0.6**	**(-10.4, 9.2)**	
**Stahl 2006**		TGCC pts 12-24 months after therapy + general popul controls	SCSA	12	14.30	8.70	278	21.40	10.60	**-7.1**	**(-12.2, -2)**	

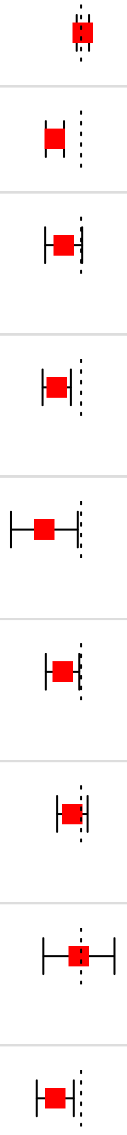

[1] Standar deviation [2] Mean difference [3] 95% Confidence Interval

Studies	Comparison	Population	Assay	Group 1 N	Group 1 Mean	Group 1 SD[1]	Group 2 N	Group 2 Mean	Group 2 SD[1]	MD[2]	95%-CI[3]	Visualization
**Stahl 2006**		TGCC pts 36-60 months after therapy + general popul controls	SCSA	17	14.10	8.40	278	21.40	10.60	**-7.3**	**(-11.5, -3.1)**	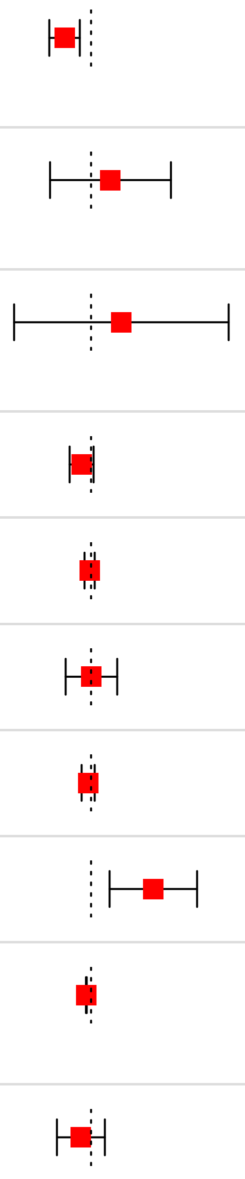
**Cortés-Gutiérrez 2017**	HPV high risk - HPV low risk	fertility clinic	SCD	3	14.67	13.61	3	9.33	5.51	**5.3**	**(-11.3, 22)**	
**Cortés-Gutiérrez 2017**		patients with condylomas	SCD	2	16.00	21.21	3	7.67	2.89	**8.3**	**(-21.2, 37.9)**	
**Grosen 2019**	IBD pts on Thiopurines - no IBD	IBD patients + general population	SCSA	40	16.80	7.10	40	19.40	7.80	**-2.6**	**(-5.9, 0.7)**	
**Grosen 2019**		IBD patients + general population	neutral Comet	40	5.30	2.80	40	5.70	3.50	**-0.4**	**(-1.8, 1)**	
**Grosen 2019**	IBD pts on Vendolizumab - no IBD	IBD patients + general population	SCSA	15	19.50	13.20	40	19.40	7.80	**0.1**	**(-7, 7.2)**	
**Grosen 2019**		IBD patients + general population	neutral Comet	15	4.90	2.80	40	5.70	3.50	**-0.8**	**(-2.6, 1)**	
**Wijesekara 2020**	Lead positive semen - negative	fertility clinic	SCD	20	39.80	25.08	20	22.65	11.30	**17.1**	**(5.1, 29.2)**	
**Elbardisi 2018**	MENA (Middle East/North Africa) - Non-MENA	fertility clinic	SCD	726	26.65	0.69	324	27.94	0.95	**-1.3**	**(-1.4, -1.2)**	
**Le 2020**	metabolic syndrome - no metabolic syndrome	patients with BMI<23	SCD	16	18.50	11.58	118	21.29	18.61	**-2.8**	**(-9.4, 3.8)**	

[1] Standar deviation [2] Mean difference [3] 95% Confidence Interval

Studies	Comparison	Population	Assay	Group 1			Group 2					Visualization
				N	Mean	SD[1]	N	Mean	SD[1]	MD[2]	95%-CI[3]	
**Le 2020**		patients with BMI>=23	SCD	49	28.34	21.91	107	22.09	14.59	**6.2**	**(-0.5, 13)**	
**Le 2021**		fertility clinic	SCD	125	25.80	18.60	409	25.90	18.20	**-0.1**	**(-3.8, 3.6)**	
**Jurewicz 2018**	Mixed diet - Prudent diet	fertility clinic	SCSA	90	16.04	9.05	96	15.20	10.45	**0.8**	**(-2, 3.6)**	
**Safarinejad 2010**		mustard gas injured and not, fertile and infertile	SCSA	134	34.30	8.00	134	28.70	8.10	**5.6**	**(3.7, 7.5)**	
**Safarinejad 2010**	mustard gas injured - not mustard gas injured	fertile	SCSA	68	27.20	2.40	66	21.40	2.30	**5.8**	**(5, 6.6)**	
**Safarinejad 2010**		fertility clinic	SCSA	66	41.60	4.40	68	35.80	4.50	**5.8**	**(4.3, 7.3)**	
**Savasi 2018**	NRTI + Efavirenz - only NRTI	HIV pts on HAART	SCD	19	39.30	21.20	16	35.20	20.20	**4.1**	**(-9.6, 17.8)**	
**Savasi 2018**	NRTI + protease inhibitor - only NRTI	HIV pts on HAART	SCD	18	35.40	14.80	16	35.20	20.20	**0.2**	**(-11.8, 12.2)**	
**Smith 2007**	orchidopexia - no orchidopexia	orchidopexia patients + idiopathic oligozoospermic controls + normozoospermic healthy controls	SCSA	18	26.40	10.10	45	16.70	11.50	**9.7**	**(4, 15.4)**	

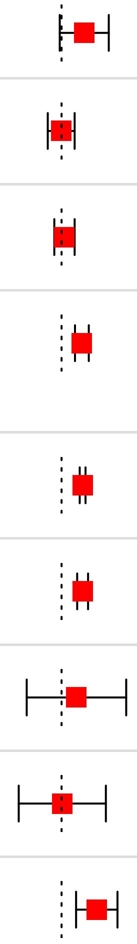

[1] Standar deviation [2] Mean difference [3] 95% Confidence Interval

				Group 1			Group 2					
**Studies**	**Comparison**	**Population**	**Assay**	**N**	**Mean**	**SD[1]**	**N**	**Mean**	**SD[1]**	**MD[2]**	**95%-CI[3]**	**Visualization**
**Smith 2007**		orchidopexia patients + idiopathic oligozoospermic controls + normozoospermic healthy controls	TUNEL	18	29.10	3.90	45	19.80	6.70	**9.3**	**(6.6, 12)**	
**Rago 2013**	phone use 2-4h/day - phone use=0h/day	fertile	TUNEL	17	3.10	9.10	10	3.00	3.80	**0.1**	**(-4.8, 5)**	
**Rago 2013**	phone use<2h/day - phone use=0h/day	fertile	TUNEL	16	3.20	6.40	10	3.00	3.80	**0.2**	**(-3.7, 4.1)**	
**Rago 2013**	phone use>4h/day - phone use=0h/day	fertile	TUNEL	20	6.60	9.80	10	3.00	3.80	**3.6**	**(-1.3, 8.5)**	
**Spano 2005**	Poland - Greenland	general population	SCSA	141	12.20	8.90	193	9.00	6.70	**3.2**	**(1.5, 4.9)**	
**Specht 2012**		fertile	SCSA	143	12.20	8.80	198	9.10	6.80	**3.1**	**(1.4, 4.8)**	
**Specht 2012**		fertile	TUNEL	134	15.20	15.40	198	4.50	8.20	**10.7**	**(7.9, 13.5)**	
**Stronati 2006**		mainly fertile	TUNEL	134	14.60	8.00	200	3.70	1.90	**10.9**	**(9.5, 12.3)**	
**Krüger 2008**	Poland - Greenland (lower xenobiotic exposure)	fertile	SCSA	69	17.90	9.70	53	10.70	5.90	**7.2**	**(4.4, 10)**	
**Long 2007**		fertile	TUNEL	69	27.00	16.50	54	5.30	3.30	**21.7**	**(17.7, 25.7)**	

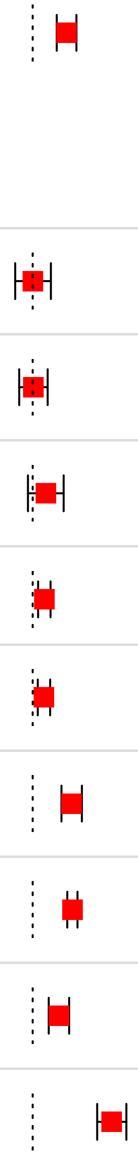

[1] Standar deviation [2] Mean difference [3] 95% Confidence Interval

				Group 1			Group 2					
**Studies**	**Comparison**	**Population**	**Assay**	**N**	**Mean**	**SD[1]**	**N**	**Mean**	**SD[1]**	**MD[2]**	**95%-CI[3]**	**Visualization**
**Giwercman 2007**	Poland (lower expected POP-exposure) - Greenland (higher expected POP-exposure)	mainly fertile	SCSA	167	12.00	7.80	188	9.00	5.90	**3.0**	**(1.5, 4.5)**	
**Giwercman 2007**	Poland (lower expected POP-exposure) - Sweden (higher expected POP-exposure)	mainly fertile	SCSA	167	12.00	7.80	178	19.00	13.00	**-7.0**	**(-9.2, -4.8)**	
**Giwercman 2007**	Poland (lower expected POP-exposure) - Ukraine (higher expected POP-exposure)	mainly fertil	SCSA	167	12.00	7.80	147	13.00	8.50	**-1.0**	**(-2.8, 0.8)**	
**Osadchuk 2014**	prostatitis - no prostatitis	general population	SCSA	9	28.38	6.23	22	15.02	0.94	**13.4**	**(9.3, 17.4)**	
**Grosen 2021**	Pts on MTX - no MTX	MTX-treated + general population	SCSA	14	13.50	6.10	40	18.40	8.70	**-4.9**	**(-9.1, -0.7)**	
**Kumar 2013**	Radiation - no radiation	hospital workers: occupationally exposed to ionizing radiation - not exposed	TUNEL	83	9.24	6.29	51	7.57	4.50	**1.7**	**(-0.2, 3.5)**	
**Stahl 2004**	radiotherapy - no tumor	TGCC patients + general population	SCSA	19	24.00	14.20	278	16.60	8.40	**7.4**	**(0.9, 13.9)**	
**Stahl 2004**		TGCC patients 6 months after therapy + general population	SCSA	4	25.50	23.80	278	16.60	8.40	**8.9**	**(-14.4, 32.2)**	

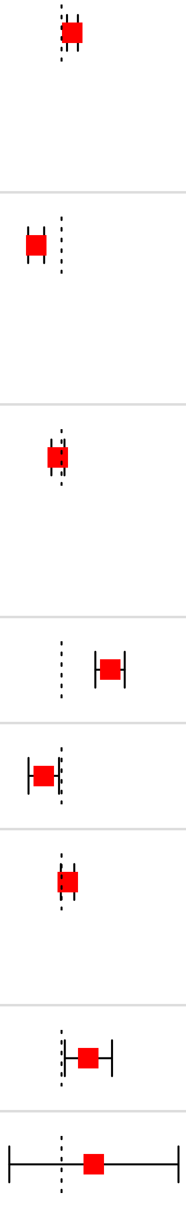

[1] Standar deviation [2] Mean difference [3] 95% Confidence Interval

				Group 1			Group 2					
**Studies**	**Comparison**	**Population**	**Assay**	**N**	**Mean**	**SD[1]**	**N**	**Mean**	**SD[1]**	**MD[2]**	**95%-CI[3]**	**Visualization**
**Stahl 2004**		TGCC pts 12-24 months after therapy + general popul controls	SCSA	10	17.10	9.50	278	16.60	8.40	**0.5**	**(-5.5, 6.5)**	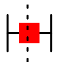
**Stahl 2004**		TGCC pts 30-60 months after therapy + general popul controls	SCSA	10	23.90	18.90	278	16.60	8.40	**7.3**	**(-4.5, 19.1)**	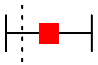
**Stahl 2006**		TGCC patients 6 months after therapy + general population	TUNEL	4	23.10	22.60	278	16.60	8.40	**6.5**	**(-15.7, 28.7)**	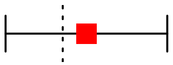
**Stahl 2006**		TGCC pts 12-24 months after therapy + general popul controls	TUNEL	13	20.60	12.40	24	13.90	7.30	**6.7**	**(-0.6, 14)**	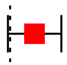
**Stahl 2006**		TGCC pts 36-60 months after therapy + general popul controls	TUNEL	13	14.90	8.50	24	13.90	7.30	**1.0**	**(-4.5, 6.5)**	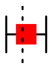
**Stahl 2006**		TGCC patients 6 months after therapy + general population	SCSA	8	21.80	13.60	278	21.40	10.60	**0.4**	**(-9.1, 9.9)**	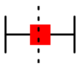
**Stahl 2006**		TGCC pts 12-24 months after therapy + general popul controls	SCSA	16	22.70	10.50	278	21.40	10.60	**1.3**	**(-4, 6.6)**	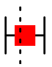
**Stahl 2006**		TGCC pts 36-60 months after therapy + general popul controls	SCSA	14	25.40	15.90	278	21.40	10.60	**4.0**	**(-4.4, 12.4)**	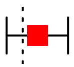
**Gill 2019**	sedentary work - active work	general population	SCD	152	24.76	15.01	102	19.99	13.73	**4.8**	**(1.2, 8.3)**	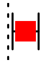

[1] Standar deviation [2] Mean difference [3] 95% Confidence Interval

				Group 1			Group 2					
Studies	Comparison	Population	Assay	N	Mean	SD[1]	N	Mean	SD[1]	MD[2]	95%-CI[3]	Visualization
**Gao 2020**	semen sample collected at clinic first - collected at home first	fertility clinic	TUNEL	49	21.10	9.20	53	22.70	12.10	**-1.6**	**(-5.8, 2.6)**	
**Wang 2018**	sleep duration<=6,5 h/day - sleep duration >6,5 h/day	general population	SCSA	40	13.10	10.10	667	12.50	9.50	**0.6**	**(-2.6, 3.8)**	
**Wang 2018**	sleep duration<7 h/day - sleep duration >7 h/day	general population	SCSA	121	12.80	10.30	586	12.50	9.40	**0.3**	**(-1.7, 2.3)**	
**Wang 2018**	sleep duration<7,5 h/day - sleep duration >7,5 h/day	general population	SCSA	257	12.30	9.30	372	12.60	9.60	**-0.3**	**(-1.8, 1.2)**	
**Wang 2018**	sleep duration<8 h/day - sleep duration >8 h/day	general population	SCSA	434	12.50	9.30	273	12.70	10.00	**-0.2**	**(-1.7, 1.3)**	
**Wang 2018**	sleep duration<8,5 h/day - sleep duration >8,5 h/day	general population	SCSA	577	12.70	9.60	130	11.70	9.20	**1.0**	**(-0.8, 2.8)**	
**Wang 2018**	sleep duration<9 h/day - sleep duration >9 h/day	general population	SCSA	655	12.70	9.70	52	10.00	7.50	**2.7**	**(0.5, 4.9)**	
**Vargas-Baquero 2020**	spinal cord injury - no spinal cord injury	spinal cord injured patients + fertile controls	SCD	27	76.90	16.30	10	16.10	4.90	**60.8**	**(53.9, 67.7)**	
**Brackett 2008**	spinal cord injury (anejaculation) - no spinal cord injury	spinal cord injury (SCI) & non-SCI controls	SCSA	10	65.20	20.90	12	15.40	10.00	**49.8**	**(35.7, 63.9)**	
**Eisenberg 2014**	Sports <1 time/week - Sports >=1 time/week	general population	SCSA	270	20.30	9.80	198	19.60	11.20	**0.7**	**(-1.2, 2.6)**	

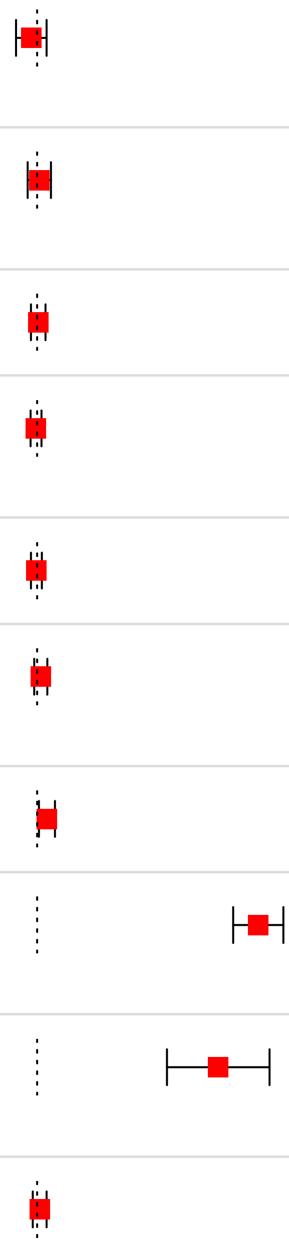

[1] Standar deviation [2] Mean difference [3] 95% Confidence Interval

				Group 1			Group 2					
Studies	Comparison	Population	Assay	N	Mean	SD[1]	N	Mean	SD[1]	MD[2]	95%-CI[3]	Visualization
**Safarinejad 2008**	SSRI th - no SSRI	fertile	SCSA	74	43.20	98.10	44	21.40	70.30	**21.8**	**(-8.7, 52.3)**	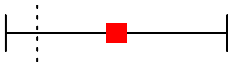
**Stahl 2004**	surgery only - no tumor	TGCC patients + general population	SCSA	20	15.60	8.20	278	16.60	8.40	**-1.0**	**(-4.7, 2.7)**	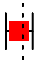
**Stahl 2004**		TGCC patients 6 months after therapy + general population	SCSA	20	17.80	9.30	278	16.60	8.40	**1.2**	**(-3, 5.4)**	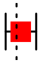
**Stahl 2004**		TGCC pts 12-24 months after therapy + general popul controls	SCSA	20	17.80	9.30	278	16.60	8.40	**1.2**	**(-3, 5.4)**	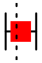
**Stahl 2004**		TGCC pts 30-60 months after therapy + general popul controls	SCSA	20	17.80	9.30	278	16.60	8.40	**1.2**	**(-3, 5.4)**	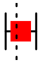
**Stahl 2006**		TGCC patients 6 months after therapy + general population	TUNEL	19	13.20	6.90	278	16.60	8.40	**-3.4**	**(-6.7, -0.1)**	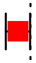
**Stahl 2006**		TGCC pts 12-24 months after therapy + general popul controls	TUNEL	19	13.20	6.90	24	13.90	7.30	**-0.7**	**(-5, 3.6)**	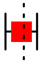
**Stahl 2006**		TGCC pts 36-60 months after therapy + general popul controls	TUNEL	19	13.20	6.90	24	13.90	7.30	**-0.7**	**(-5, 3.6)**	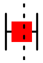
**Stahl 2006**		TGCC patients 6 months after therapy + general population	SCSA	25	25.80	16.50	278	21.40	10.60	**4.4**	**(-2.2, 11)**	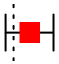

[1] Standar deviation [2] Mean difference [3] 95% Confidence Interval

				Group 1			Group 2					
**Studies**	**Comparison**	**Population**	**Assay**	**N**	**Mean**	**SD[1]**	**N**	**Mean**	**SD[1]**	**MD[2]**	**95%-CI[3]**	**Visualization**
**Stahl 2006**		TGCC pts 12-24 months after therapy + general popul controls	SCSA	25	25.80	16.50	278	21.40	10.60	**4.4**	**(-2.2, 11)**	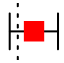
**Stahl 2006**		TGCC pts 36-60 months after therapy + general popul controls	SCSA	25	25.80	16.50	278	21.40	10.60	**4.4**	**(-2.2, 11)**	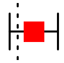
**Spano 2005**	Sweden - Greenland	general population	SCSA	178	18.60	15.90	193	9.00	6.70	**9.6**	**(7.1, 12.1)**	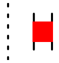
**Stronati 2006**		mainly fertile	TUNEL	166	13.70	8.00	200	3.70	1.90	**10.0**	**(8.8, 11.2)**	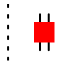
**Krüger 2008**	Sweden - Greenland (lower xenobiotic exposure)	fertile	SCSA	93	30.60	17.30	53	10.70	5.90	**19.9**	**(16, 23.8)**	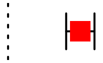
**Long 2007**		fertile	TUNEL	81	24.10	15.20	54	5.30	3.30	**18.8**	**(15.4, 22.2)**	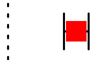
**Abdullah 2020**	testicular atrophy - no testicular atrophy	patients with varicocele	SCD	20	29.70	5.00	121	35.30	11.60	**-5.6**	**(-8.6, -2.6)**	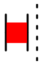
**Kavoussi 2021**		patients with varicocele	SCD	20	29.70	5.00	121	35.30	11.60	**-5.6**	**(-8.6, -2.6)**	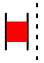
**Vujkovic 2009**	Traditional Dutch' diet HIGH - Health Conscious' HIGH	fertility clinic	SCSA	39	22.00	1.70	42	20.60	1.40	**1.4**	**(0.7, 2.1)**	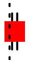
**Vujkovic 2009**	Traditional Dutch' INTERMEDIATE - Health Conscious' HIGH	fertility clinic	SCSA	43	25.00	2.00	42	20.60	1.40	**4.4**	**(3.7, 5.1)**	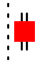

[1] Standar deviation [2] Mean difference [3] 95% Confidence Interval

				Group 1			Group 2					
**Studies**	**Comparison**	**Population**	**Assay**	**N**	**Mean**	**SD[1]**	**N**	**Mean**	**SD[1]**	**MD[2]**	**95%-CI[3]**	**Visualization**
**Vujkovic 2009**	Traditional Dutch' LOW - Health Conscious' HIGH	fertility clinic	SCSA	44	23.50	1.50	42	20.60	1.40	**2.9**	**(2.3, 3.5)**	
**Malm 2017**	Tromsoe, summer - Oslo, summer (more melatinon, thus more antioxidants)	general population	SCSA	88	12.00	8.80	110	12.00	7.40	**0.0**	**(-2.3, 2.3)**	
**Malm 2017**	Tromsoe, winter - Oslo, winter (more melatinon, thus more antioxidants)	general population	SCSA	85	12.00	7.50	108	11.00	6.10	**1.0**	**(-1, 3)**	
**Spano 2005**	Ukraine - Greenland	general population	SCSA	195	13.30	8.70	193	9.00	6.70	**4.3**	**(2.8, 5.8)**	
**Specht 2012**		fertile	SCSA	207	13.30	18.30	198	9.10	6.80	**4.2**	**(1.5, 6.9)**	
**Specht 2012**		fertile	TUNEL	133	9.30	9.60	198	4.50	8.20	**4.8**	**(2.8, 6.8)**	
**Stronati 2006**		mainly fertile	TUNEL	152	9.00	5.50	200	3.70	1.90	**5.3**	**(4.4, 6.2)**	
**Krüger 2008**	Ukraine - Greenland (lower xenobiotic exposure)	fertile	SCSA	85	14.90	7.40	53	10.70	5.90	**4.2**	**(2, 6.4)**	
**Long 2007**		fertile	TUNEL	58	19.60	14.00	54	5.30	3.30	**14.3**	**(10.6, 18)**	
**Lenters 2015**	Ukraine - Poland (lower POP exposure)	fertile	TUNEL	197	11.64	127.90	206	6.46	103.50	**5.2**	**(-17.6, 28)**	
**Lenters 2015**		fertile	SCSA	206	10.83	102.60	197	10.07	97.90	**0.8**	**(-18.8, 20.3)**	

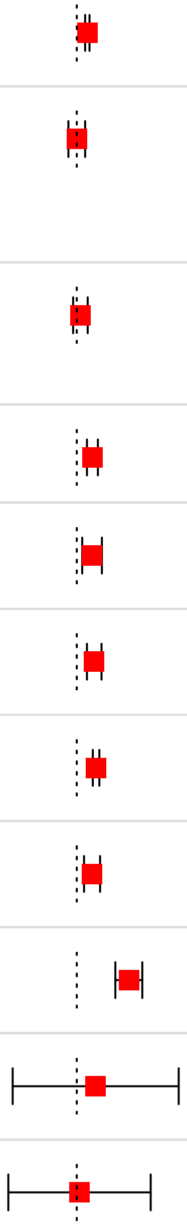

[1] Standar deviation [2] Mean difference [3] 95% Confidence Interval

Studies	Comparison	Population	Assay	Group 1			Group 2					
				N	Mean	SD[1]	N	Mean	SD[1]	MD[2]	95%-CI[3]	Visualization
**van Brakel 2017**	undescended testes - no undescended testes	fertility clinic + fertile controls	SCSA	99	21.80	11.50	22	15.70	5.10	**6.1**	**(3, 9.2)**	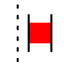
**Banks 2021**	vit D<20 ng/ml - vit D>=20 ng/ml	fertility clinic	SCSA	23	23.00	17.00	112	20.60	10.30	**2.4**	**(-4.8, 9.6)**	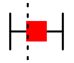
**Lu 2018**	Waist-to-height ratio>=0,5 - Waist-to-height ratio<0,5	fertility clinic	SCSA	324	17.80	11.91	686	19.00	12.71	**-1.2**	**(-2.8, 0.4)**	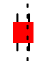
**Pelliccione 2011**	waist circumference >94 cm - waist circumference <94 cm	sportsmen	continuous	2	5.50	3.50	5	6.50	2.00	**-1.0**	**(-6.2, 4.2)**	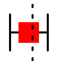
**Eisenberg 2014**	WC>=101,6 cm - WC<101,59 cm	general population	SCSA	183	19.30	8.60	281	20.00	11.50	**-0.7**	**(-2.5, 1.1)**	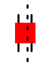
**Lu 2018**	WC>=90 cm - WC<90 cm	fertility clinic	SCSA	203	18.04	12.33	807	18.76	12.50	**-0.7**	**(-2.6, 1.2)**	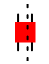
**Eisenberg 2014**	WC>=94 cm - WC <93,99	general population	SCSA	310	19.70	10.10	154	19.80	11.20	**-0.1**	**(-2.2, 2)**	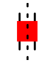
**Jurewicz 2018**	Western diet - Mixed diet	fertility clinic	SCSA	150	17.98	8.12	90	16.04	9.05	**1.9**	**(-0.3, 4.2)**	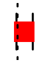
**Jurewicz 2018**	Western diet - Prudent diet	fertility clinic	SCSA	150	17.98	8.12	96	15.20	10.45	**2.8**	**(0.3, 5.2)**	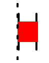
**Lu 2018**	WHR>=0,9 - WHR<0,9	fertility clinic	SCSA	263	18.24	12.26	747	18.75	12.54	**-0.5**	**(-2.2, 1.2)**	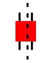

[1] Standar deviation [2] Mean difference [3] 95% Confidence Interval
