# Supplementary material for: Lifestyle-, environmental-, and additional health factors associated with an increased sperm DNA fragmentation: a systematic review and meta-analysis
Source: Reprod Biol Endocrinol. 2023 Jan 18;21:5. doi: 10.1186/s12958-023-01054-0 (PMC9847125; doi:10.1186/s12958-023-01054-0)
Supplement: Supplementary file 1 — Additional file 1: Supplementary Appendix 1. Risk of bias assessment methodology. Supplementary Table 1. PRISMA 2020 checklist. Supplementary Table 2. Basic characteristics of the included article. Supplementary Table 3. Eligibility criteria in each included article. Supplementary Table 4. Risk factor and population definitions in each included article. Supplementary Table 5. Risk of bias assessment using the QUIPS tools. Supplementary Table 6. Articles also looking at pregnancy or birth as an outcome. Supplementary Figure 1. Comparison of patients’ sperm DNA fragmentation values with and without varicocele subdivided based on sperm DNA fragmentation assays used (continuous data). Supplementary Figure 2. Comparison of patients’ sperm DNA fragmentation values with and without varicocele subdivided based on different cut-off values. Supplementary Figure 3. Supplementary Figure 4. Comparison of patients’ sperm DNA fragmentation values with and without varicocele subdivided based on the fertility status of patients (continuous data). Supplementary Figure 5. Comparison of patients’ sperm DNA fragmentation values with impaired and normal glucose tolerance (continuous data). Supplementary Figure 6. Comparison of patients’ sperm DNA fragmentation values with and without testicular tumors subdivided based on sperm DNA fragmentation assays used (continuous data). Supplementary Figure 7. Comparison of patients’ sperm DNA fragmentation values with and without Hodgkin-lymphoma (HL) (continuous data). Supplementary Figure 8. Comparison of patients’ sperm DNA fragmentation values with and without non-Hodgkin lymphoma (NHL) (continuous data). Supplementary Figure 9. Comparison of patients’ sperm DNA fragmentation values with and without lymphomas (continuous data). Supplementary Figure 10. Comparison of patients’ sperm DNA fragmentation values with and without leukemia (continuous data). Supplementary Figure 11. Comparison of patients’ sperm DNA fragmentation values with and withou [file 12958_2023_1054_MOESM1_ESM.zip › ESM1/Supplementary Figure 47.pdf]

Mean differences of Maradék - continuous - ua popul

				Group 1			Group 2					
**Studies**	**Comparison**	**Population**	**Assay**	**N**	**Mean**	**SD[1]**	**N**	**Mean**	**SD[1]**	**MD[2]**	**95%-CI[3]**	**Visualization**
**Falahieh 2021**	14 days after COVID dg - 120 days after COVID dg	fertile	SCD	20	33.10	7.70	20	25.70	11.30	**7.4**	**(-0.9, 15.7)**	
**Pons 2013**	abstinence=1 day - abstinence=3-7 days	men of DFI>=30	SCD	32	20.40	6.60	32	40.60	10.80	**-20.2**	**(-26.2, -14.2)**	
**Rubes 2010**	high pollution - low pollution	general population	SCSA	47	7.31	3.64	47	5.46	3.21	**1.8**	**(-0.1, 3.8)**	
**Rubes 2010**		smokers	SCSA	15	7.07	2.90	15	5.67	2.87	**1.4**	**(-1.5, 4.3)**	
**Rubes 2010**		non-smokers	SCSA	32	7.42	3.98	32	5.37	3.40	**2.0**	**(-0.5, 4.6)**	
**Grosen 2019**	IBD pts off anti-TNFa (off to begin with) - IBD pts off anti-TNFa	IBD patients	SCSA	19	17.70	10.00	19	14.00	7.60	**3.7**	**(-4.2, 11.6)**	
**Grosen 2019**		IBD patients	neutral Comet	19	4.40	3.60	19	3.90	3.10	**0.5**	**(-2.5, 3.5)**	
**Grosen 2019**	IBD pts on anti-TNFa (on to begin with) - IBD pts off anti-TNFa (remission)	IBD patients	SCSA	17	18.50	8.40	17	15.30	8.60	**3.2**	**(-4.9, 11.3)**	
**Grosen 2019**		IBD patients	neutral Comet	17	3.90	2.20	17	3.80	3.20	**0.1**	**(-2.5, 2.7)**	
**Grosen 2019**	IBD pts on Thiopurines - IBD pts off Thiopurines	IBD patients	SCSA	10	13.50	4.90	10	11.30	3.60	**2.2**	**(-3.1, 7.5)**	
**Grosen 2019**		IBD patients	neutral Comet	10	3.80	2.60	10	5.70	5.20	**-1.9**	**(-6.7, 2.9)**	

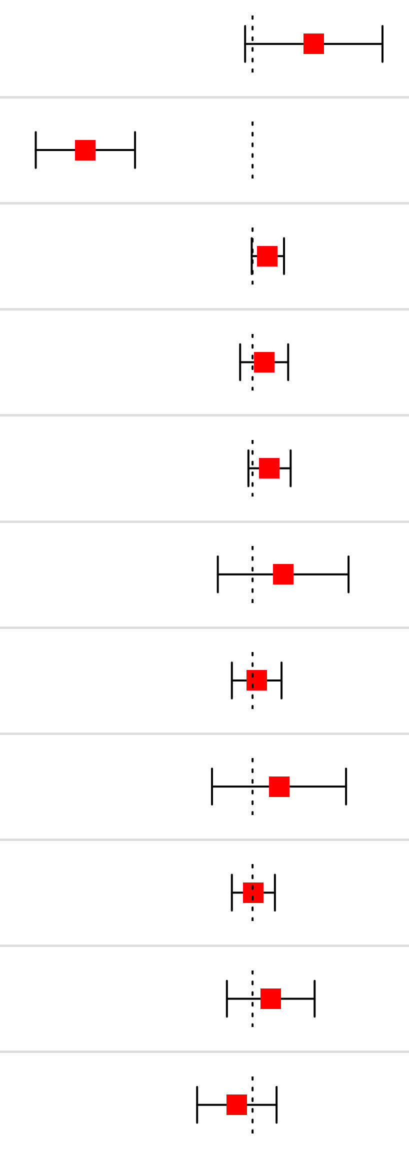

[1] Standar deviation [2] Mean difference [3] 95% Confidence Interval

				Group 1			Group 2					
**Studies**	**Comparison**	**Population**	**Assay**	**N**	**Mean**	**SD[1]**	**N**	**Mean**	**SD[1]**	**MD[2]**	**95%-CI[3]**	**Visualization**
**Grosen 2019**	IBD pts on Vendolizumab - IBD pts before Vendolizumab	IBD patients	SCSA	3	7.80	6.30	3	7.50	1.20	**0.3**	**(-8.2, 8.8)**	
**Grosen 2019**		IBD patients	neutral Comet	3	2.50	2.90	3	1.20	2.10	**1.3**	**(-4.4, 7)**	
**Grosen 2021**	on MTX - off MTX	MTX-treated	SCSA	5	11.50	3.40	5	14.80	6.40	**-3.3**	**(-11.9, 5.3)**	
**Malm 2017**	Oslo, winter - Oslo, summer (more melatinon, thus more antioxidants)	general population	SCSA	108	11.00	6.10	110	12.00	7.40	**-1.0**	**(-3.5, 1.5)**	
**Gao 2020**	semen sample collected at clinic - collected at home	fertility clinic	TUNEL	102	24.20	11.80	102	25.70	12.70	**-1.5**	**(-6.3, 3.3)**	
**Grosen 2019**	severe IBD - severe IBD remission after iv CS	IBD patients	SCSA	20	22.30	15.00	20	15.80	6.70	**6.5**	**(-3, 16)**	
**Grosen 2019**		IBD patients	neutral Comet	20	5.80	4.60	20	6.80	5.90	**-1.0**	**(-5.6, 3.6)**	
**Malm 2017**	Tromsoe, winter - Tromsoe, summer (more melatinon, thus more antioxidants)	general population	SCSA	85	12.00	7.50	88	12.00	8.80	**0.0**	**(-3.5, 3.5)**	

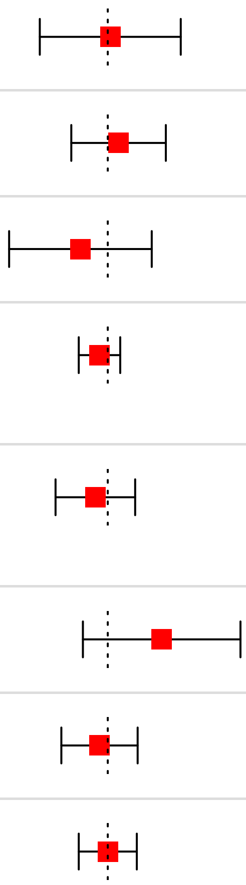

[1] Standar deviation [2] Mean difference [3] 95% Confidence Interval
