# Supplementary material for: Lifestyle-, environmental-, and additional health factors associated with an increased sperm DNA fragmentation: a systematic review and meta-analysis
Source: Reprod Biol Endocrinol. 2023 Jan 18;21:5. doi: 10.1186/s12958-023-01054-0 (PMC9847125; doi:10.1186/s12958-023-01054-0)
Supplement: Supplementary file 1 — Additional file 1: Supplementary Appendix 1. Risk of bias assessment methodology. Supplementary Table 1. PRISMA 2020 checklist. Supplementary Table 2. Basic characteristics of the included article. Supplementary Table 3. Eligibility criteria in each included article. Supplementary Table 4. Risk factor and population definitions in each included article. Supplementary Table 5. Risk of bias assessment using the QUIPS tools. Supplementary Table 6. Articles also looking at pregnancy or birth as an outcome. Supplementary Figure 1. Comparison of patients’ sperm DNA fragmentation values with and without varicocele subdivided based on sperm DNA fragmentation assays used (continuous data). Supplementary Figure 2. Comparison of patients’ sperm DNA fragmentation values with and without varicocele subdivided based on different cut-off values. Supplementary Figure 3. Supplementary Figure 4. Comparison of patients’ sperm DNA fragmentation values with and without varicocele subdivided based on the fertility status of patients (continuous data). Supplementary Figure 5. Comparison of patients’ sperm DNA fragmentation values with impaired and normal glucose tolerance (continuous data). Supplementary Figure 6. Comparison of patients’ sperm DNA fragmentation values with and without testicular tumors subdivided based on sperm DNA fragmentation assays used (continuous data). Supplementary Figure 7. Comparison of patients’ sperm DNA fragmentation values with and without Hodgkin-lymphoma (HL) (continuous data). Supplementary Figure 8. Comparison of patients’ sperm DNA fragmentation values with and without non-Hodgkin lymphoma (NHL) (continuous data). Supplementary Figure 9. Comparison of patients’ sperm DNA fragmentation values with and without lymphomas (continuous data). Supplementary Figure 10. Comparison of patients’ sperm DNA fragmentation values with and without leukemia (continuous data). Supplementary Figure 11. Comparison of patients’ sperm DNA fragmentation values with and withou [file 12958_2023_1054_MOESM1_ESM.zip › ESM1/Supplementary Figure 48.pdf]

Odds ratios of Maradék - cut-off

				Group 1		Group 2				
**Studies**	**Comparison**	**Population**	**Assay**	**Event**	**N**	**Event**	**N**	**OR[1]**	**95%-CI[2]**	**Visualization**
**van Brakel 2017**	acquired undescended testes - fertile	follow-up on fertility + fertile controls	SCSA	7	49	0	22	**7.9**	**(0.4, 145.5)**	
**Bojar 2013**	age>=26 - age<26	fertility clinic	SCSA	56	150	7	35	**2.4**	**(1, 5.8)**	
**Bojar 2013**		fertility clinic	SCSA	92	150	10	35	**4.0**	**(1.8, 8.9)**	
**Martínez 2021**	age>=40 - age<=39	fertility clinic	TUNEL	30	79	32	84	**1.0**	**(0.5, 1.9)**	
**Gao 2021**	age>=40 - age<40	fertility clinic	SCSA	1849	2774	7558	15667	**2.1**	**(2, 2.3)**	
**Gill 2020**	age>29 - age<=29	fertility clinic	SCD	251	462	83	213	**1.9**	**(1.3, 2.6)**	
**Bojar 2013**	age>35 - age<=35	fertility clinic	SCSA	32	41	70	144	**3.8**	**(1.7, 8.4)**	
**Bojar 2013**		fertility clinic	SCSA	25	41	38	144	**4.4**	**(2.1, 9)**	
**Eisenberg 2014**	BMI 25-29,9 - BMI<25	general population	SCSA	171	191	76	82	**0.7**	**(0.3, 1.7)**	
**Eisenberg 2014**	BMI 30-34,9 - BMI<25	general population	SCSA	112	119	76	82	**1.3**	**(0.4, 3.9)**	
**Le 2020**	BMI>=23 - BMI<23	fertility clinic	SCD	47	156	25	134	**1.9**	**(1.1, 3.3)**	
**Eisenberg 2014**	BMI>=25 - BMI<25	general population	SCSA	349	377	76	82	**1.0**	**(0.4, 2.5)**	

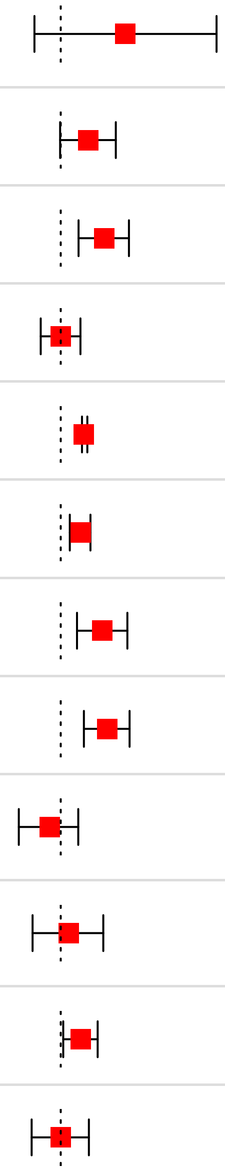

[1] Odds ratio [2] 95% Confidence Interval

				Group 1		Group 2				
Studies	Comparison	Population	Assay	Event	N	Event	N	OR[1]	95%-CI[2]	Visualization
**Pearce 2019**		fertility clinic	SCD	18	26	1	3	**4.5**	**(0.4, 57.1)**	
**Pearce 2019**		fertility clinic	SCD	8	26	1	3	**0.9**	**(0.1, 11.3)**	
**Pearce 2019**		fertility clinic	SCD	3	26	0	3	**1.0**	**(0, 24.8)**	
**Pearce 2019**		fertility clinic	SCD	1	26	0	3	**0.4**	**(0, 12.2)**	
**Eisenberg 2014**	BMI>=35 - BMI<25	general population	SCSA	66	67	76	82	**5.2**	**(0.6, 44.4)**	
**Pearce 2019**	BMI>30 - BMI<=30	fertility clinic	SCD	12	14	7	15	**6.9**	**(1.1, 41.8)**	
**Pearce 2019**		fertility clinic	SCD	7	14	2	15	**6.5**	**(1.1, 40.1)**	
**Pearce 2019**		fertility clinic	SCD	2	14	1	15	**2.3**	**(0.2, 29)**	
**Pearce 2019**		fertility clinic	SCD	1	14	0	15	**3.4**	**(0.1, 91.8)**	
**van Brakel 2017**	congenital undescended testes - fertile	follow-up on fertility + fertile controls	SCSA	9	50	0	22	**10.3**	**(0.6, 185.3)**	
**Chigrinets 2019**	drinker - abstainer	fertility clinic	SCD	11	16	12	16	**0.7**	**(0.2, 3.5)**	
**Savasi 2018**	HAART th - no HAART	HIV patients	SCD	36	53	9	24	**3.5**	**(1.3, 9.7)**	
**Savasi 2018**		HIV patients between ages 44-54	SCD	15	21	1	4	**7.5**	**(0.6, 87.2)**	

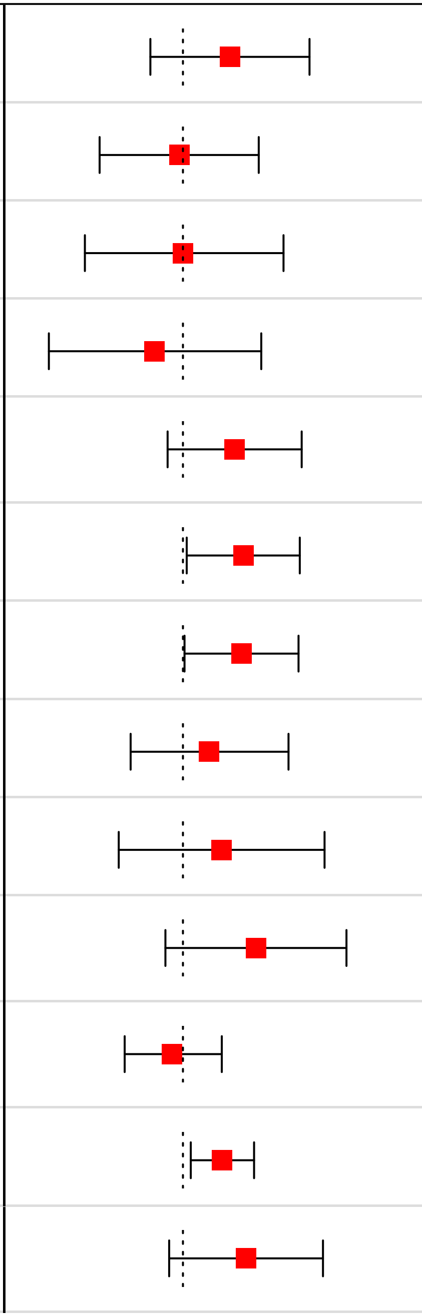

[1] Odds ratio [2] 95% Confidence Interval

				Group 1		Group 2				
**Studies**	**Comparison**	**Population**	**Assay**	**Event**	**N**	**Event**	**N**	**OR[1]**	**95%-CI[2]**	**Visualization**
**Savasi 2018**		HIV patients between ages 39-43	SCD	11	17	3	7	**2.4**	**(0.4, 14.7)**	
**Savasi 2018**		HIV patients between ages 25-38	SCD	10	15	5	13	**3.2**	**(0.7, 15.1)**	
**Humaidan 2021**	High sORP level: >1.36 mV/106 sperm/mL - Normal sORP level: 1.36 mV/106 sperm/mL	fertility clinic + general popul controls	SCSA	17	40	22	46	**0.8**	**(0.3, 1.9)**	
**Cortés-Gutiérrez 2017**	HPV high risk - HPV negative	high-risk HPV patients + HPV negative	SCD	1	5	6	27	**0.9**	**(0.1, 9.4)**	
**Elbardisi 2018**	MENA (Middle East/North Africa) - Non-MENA	fertility clinic	SCD	316	726	117	324	**1.4**	**(1, 1.8)**	
**Le 2020**	metabolic syndrome - no metabolic syndrome	fertility clinic	SCD	20	65	52	225	**1.5**	**(0.8, 2.7)**	
**Henkel 2003**	ROS positive spermatozoa - ROS negative spermatozoa	fertility clinic	TUNEL	1	24	0	20	**2.6**	**(0.1, 67.8)**	
**Gill 2019**	sedentary work - active work	general popul	SCD	110	152	56	102	**2.2**	**(1.3, 3.6)**	
**Gill 2019**		general popul	SCD	47	152	17	102	**2.2**	**(1.2, 4.2)**	
**Bojar 2013**	smoking - no smoking	fertility clinic	SCSA	29	51	74	135	**1.1**	**(0.6, 2.1)**	
**Bojar 2013**		fertility clinic	SCSA	19	51	45	135	**1.2**	**(0.6, 2.3)**	

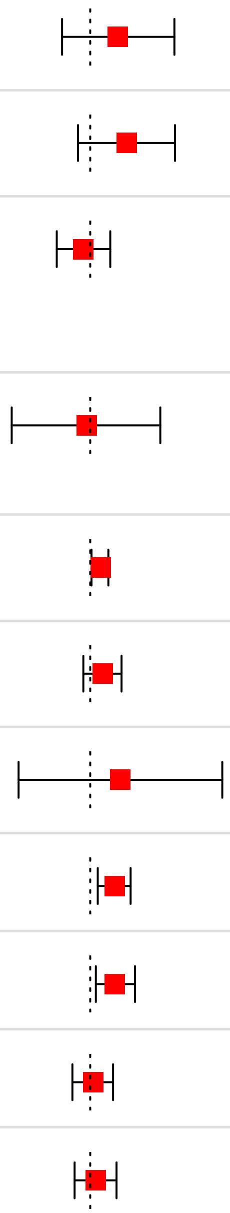

[1] Odds ratio [2] 95% Confidence Interval

				Group 1		Group 2				
**Studies**	**Comparison**	**Population**	**Assay**	**Event**	**N**	**Event**	**N**	**OR[1]**	**95%-CI[2]**	**Visualization**
**Bojar 2013**		fertility clinic	SCSA	8	51	18	135	**1.2**	**(0.5, 3)**	
**Chigrinets 2019**		fertility clinic	SCD	8	11	15	23	**1.4**	**(0.3, 6.9)**	
**Smit 2010**	testicular germ cell tumor - lymphomas	cancer patients	SCSA	11	52	14	46	**0.6**	**(0.2, 1.5)**	
**Marchlewska 2016**	testicular germ cell tumor - no tumor	fertility clinic + patients with germ cell testicular tumor	SCD	11	23	75	312	**2.9**	**(1.2, 6.8)**	
**van Brakel 2017**	undescended testes - fertile	follow-up on fertility + fertile controls	SCSA	16	99	0	22	**8.9**	**(0.5, 154)**	
**Kiwitt-Cárdenas 2021**	urinary BPA conc 1,05-1,98 mikrog/g - urinary BPA conc 0,07-0,96 mikrog/g	healthy university students	SCD	37	40	27	39	**5.5**	**(1.4, 21.3)**	
**Kiwitt-Cárdenas 2021**		healthy university students	SCD	15	40	14	39	**1.1**	**(0.4, 2.7)**	
**Kiwitt-Cárdenas 2021**	urinary BPA conc 2,08-3,43 mikrog/g - urinary BPA conc 0,07-0,96 mikrog/g	healthy university students	SCD	35	40	27	39	**3.1**	**(1, 9.9)**	
**Kiwitt-Cárdenas 2021**		healthy university students	SCD	15	40	14	39	**1.1**	**(0.4, 2.7)**	
**Kiwitt-Cárdenas 2021**	urinary BPA conc 3,85-32,5 mikrog/g - urinary BPA conc 0,07-0,96 mikrog/g	healthy university students	SCD	36	39	27	39	**5.3**	**(1.4, 20.8)**	

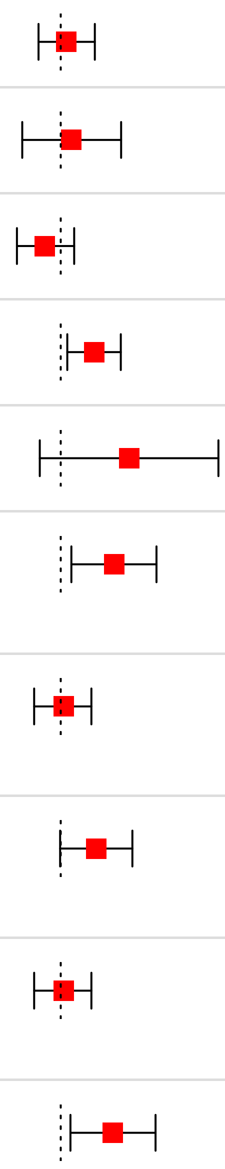

[1] Odds ratio [2] 95% Confidence Interval

				Group 1		Group 2				
**Studies**	**Comparison**	**Population**	**Assay**	**Event**	**N**	**Event**	**N**	**OR[1]**	**95%-CI[2]**	**Visualization**
**Kiwitt-Cárdenas 2021**		healthy university students	SCD	15	39	14	39	**1.1**	**(0.4, 2.8)**	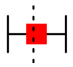
**Fernandez-Encinas 2020**	varicocele - no varicocele	fertility clinic + fertile donors	alkaline Comet	6	6	6	18	**25.0**	**(1.2, 516.7)**	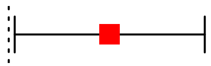
**Fernandez-Encinas 2020**		fertility clinic + fertile donors	neutral Comet	6	6	12	18	**6.8**	**(0.3, 139.7)**	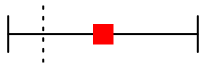
**Banks 2021**	vit D<20 ng/ml - vit D>=20 ng/ml	fertility clinic	SCSA	9	23	35	112	**1.4**	**(0.6, 3.6)**	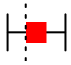

[1] Odds ratio [2] 95% Confidence Interval
